# Toxicological analysis of metabolites in ischemic stroke based on salivary metabolomics

**DOI:** 10.3389/fmolb.2025.1609227

**Published:** 2025-08-29

**Authors:** Yan-Song Liu, Yu-Yan Long, Jie Liu, Yu-Chen Liu, Shuang Zhang, Yi-Jia Xu, Shu-Yue Fu, Hua Li, Wang-Hua Liu

**Affiliations:** ^1^ Provincial Key Laboratory of TCM Diagnostics, Hunan University of Chinese Medicine, Changsha, China; ^2^ Key Laboratory of TCM Heart and Lung Syndrome Differentiation & Medicated Diet and Dietotherapy, Changsha, China; ^3^ Hunan Engineering Technology Research Center for Medicinal and Functional Food, Changsha, China; ^4^ The First Affiliated Hospital of Hunan University of Chinese Medicine, Changsha, China

**Keywords:** ischemic stroke, salivary metabolomics, biomarkers, metabolic toxicity, metabolic cascade, toxicity prediction

## Abstract

**Objective:**

To elucidate the characteristic patterns of salivary metabolic network instability in IS patients, reveal the association mechanism between amino acid-lipid-nucleotide metabolic cascade imbalance and stroke progression, and provide experimental basis and translational pathway for the development of diagnostic and therapeutic strategies based on metabolic microenvironment regulation.

**Methods:**

This study focused on salivary metabolomics. A prospective cohort design (40 IS patients and 30 healthy controls) was combined with high-resolution liquid chromatography-mass spectrometry (LC-MS/MS) to systematically analyze the molecular characteristics and toxicological mechanisms of metabolic disorders in stroke. Orthogonal partial least squares discriminant analysis (OPLS-DA) and game theory feature weighting method were used to screen differential metabolites, and toxicity evaluation was performed by integrating ADMETlab and ProTox databases. Finally, molecular docking technology was used to verify the metabolite-target interaction network.

**Results:**

A total of 488 salivary metabolites were identified, of which 167 showed significant differences between groups, including 4.3-fold increase in arginine, 3.5-fold increase in xanthine, and 2.1-fold increase in lipoxin A4. Toxicity prediction showed that xanthine has potential neurotoxicity and blood-brain barrier penetration ability (BBB = 0.90). Its molecular docking with targets such as XDH and PNP showed stable binding energy, suggesting that it participates in the pathological process of stroke by regulating purine metabolism and oxidative stress.

**Conclusion:**

A panoramic analysis framework of salivary metabolomics in ischemic stroke was constructed, and the cascade disorder of the amino acid-lipid-nucleotide metabolic network was elucidated. The screened core metabolite markers and their regulatory pathways not only provide highly specific tools for early diagnosis of stroke, but also provide research basis for the development of innovative therapies based on metabolic microenvironment regulation.

## 1 Introduction

Ischemic stroke (IS) is an acute cerebrovascular disease caused by rupture or blockage of cerebral blood vessels, which continues to be the first in disability rate and the second in mortality rate worldwide. Epidemiological data show that the incidence of stroke is showing a significant trend of younger age, with an annual growth rate of about 6.6% in people under 50 years old (Li et al., 2022; Ekker et al., 2023). The annual number of new cases in China has exceeded 2.87 million, and its incidence has reached epidemic scale worldwide. It has become the main cause of death among Chinese residents (Wen-Jun et al., 2023). IS has a rapid onset, limited treatment methods, and a short treatment time window. It is difficult to diagnose early, has a limited treatment time, significantly shortens the patient’s life cycle, and reduces the patient’s quality of life. It brings a considerable socioeconomic burden. It is estimated that the global cost of IS is about 721 billion US dollars, accounting for 0.66% of the global GDP (Feigin et al., 2022).

Currently, the clinical diagnosis of IS is mainly based on the triple method of neuroimaging evaluation, neurological function examination and medical history tracing. Rapid and non-invasive diagnostic tests are not yet available (Musuka et al., 2015). 140 h after the onset of IS, the statistical advantage of intravenous thrombolysis (IVT) combined with thrombectomy treatment disappears (Kaesmacher et al., 2024). In a prospective hospital-based stroke registry study in Chengdu, only 11% of 1,358 consecutive stroke patients arrived at the hospital within 3 h, and less than 1% of all ischemic stroke patients received alteplase treatment (Liu et al., 2007). In this context, finding biological samples with instant diagnostic potential is a must for early diagnosis and treatment of IS. Saliva has become a potential ideal sample for IS diagnostic research and mechanism analysis due to its convenience in sample collection and transportation, non-infectiousness, and high stability of analytical compounds (Maciejczyk et al., 2021; Szustkiewicz-Karoń et al., 2023; Zhanina et al., 2022). The salivary glands are in close contact with the capillary network, and the small molecular weight characteristics of metabolites make them have excellent transmembrane diffusion efficiency. Therefore, changes in metabolites in saliva are usually consistent with changes in blood, which can reflect the effects of disease, nutrition, drugs and environmental conditions on the body (Yoshizawa et al., 2013). Huang et al. (2023) believed that the emergence of mass spectrometry (MS) technology made up for the shortcomings of detecting low molecular weight compounds in saliva. The application of high-sensitivity measurement technologies such as liquid chromatography-mass spectrometry (LC-MS) can make saliva a medium for IS signature metabolites.

The innovation of metabolomics methods based on mass spectrometry technology has injected new impetus into this field. Metabolomics uses modern analytical technologies with high throughput, high sensitivity and high precision to dynamically track the overall composition of metabolites in body fluids secreted by cells and organisms to find the relative relationship between metabolites and physiological and pathological changes. LC-MS/MS can achieve a detection sensitivity of 10–9 mol/L through the synergistic effect of gradient elution chromatography and high-resolution mass spectrometry. The dynamic monitoring capability of the accelerated rate can capture the transient metabolic fluctuations in the acute phase of stroke.

This study adopted a prospective cohort design, systematically constructed a saliva metabolic analysis system for ischemic stroke, used orthogonal partial least squares discriminant analysis (OPLS-DA) combined with variance inflation factor (VIF) correction model to screen key metabolic markers, applied game theory characteristic factor weight method to analyze metabolic network topology characteristics, and finally verified the metabolite-target interaction mechanism through molecular docking technology. The study aims to break through the time-space limitations of traditional diagnostic models and provide a new paradigm for the in-depth analysis of stroke pathological mechanisms and the construction of a precise diagnosis and treatment system.

## 2 Research methods

### 2.1 Metabolite sample collection

This study adopted a prospective case-control design. From January to December 2024, confirmed cases were screened from IS patients who visited the Department of Neurology of the First Affiliated Hospital of Hunan University of Chinese Medicine. Inclusion criteria included: diffusion weighted imaging (DWI) confirmed the presence of internal carotid artery or middle cerebral artery M1 segment occlusion, and NIH Stroke Scale (NIHSS) score ≥6 points, and no oral disease, kidney disease and related metabolic diseases.

**FIGURE 1:**
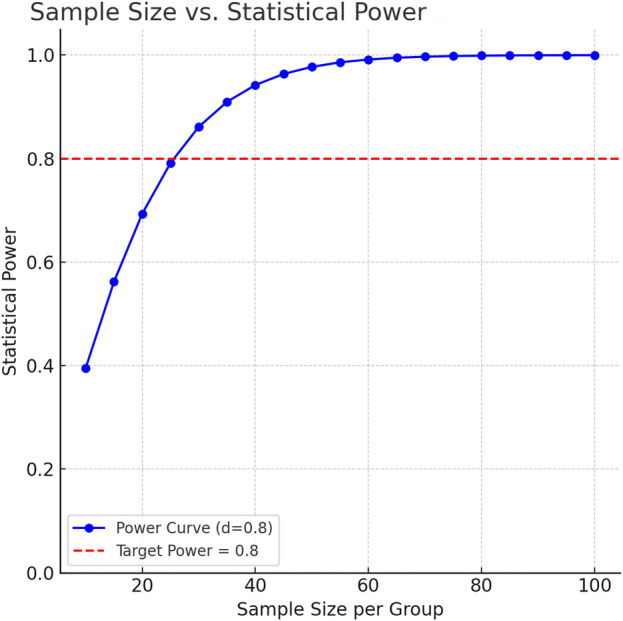
Statistical power as a function of per-group sample size.

As an exploratory saliva metabolomics analysis, the core goal of this study is to discover potential metabolic markers and pathological mechanisms of IS. According to the MetSizeR simulation framework proposed by Nyamundanda et al. (Nyamundanda et al., 2013) and the metabolomics sample size calculation standard of Billoir et al. (2015), the pwr package of R Studio (version 2025.05.0+496) was used to estimate the required number of samples. Taking the large effect size (Cohen’s d) of 0.8 that can show significant differences as the standard, the two-tailed significance level (α) was set to 0.05, and the expected statistical power (Power) was 0.8. The simulation results showed that at least 26 samples were required in each group to detect significant differences (Figure 1). Considering the heterogeneity between groups and data integrity, a total of 232 patients were initially included in this study. After excluding 15 patients with oral diseases, 73 patients with insufficient compliance, and 59 patients with hemolysis/lipidemia, 40 patients were selected from the qualified sample library by random number table as the experimental group. At the same time, 30 healthy volunteers matched in age and gender were selected as the control group. The baseline characteristics of each group are detailed in Table 1 Demographic data and clinical parameters.

**TABLE 1:** Comparison of baseline demographic characteristics and clinical indicators of subjects.

Category	Number	Gender (Male/Female)	Median age (range)	Stroke recurrence rate (%)
IS	40	29/11	67 (47–88)	12.5
CON	30	24/15	58 (42–78)	—

### 2.2 Sample collection specifications

All subjects must be fasting for 8 h before collection and are allowed to drink an appropriate amount of water. Sample collection is uniformly arranged in a standard constant temperature room (temperature 25 °C ± 1 °C, humidity 50%–60%), and the collection period is fixed at 9:30–11:30 every day. The operation process includes:

(1) Rinse the mouth three times with 10 mL of sterile saline (sodium chloride concentration 0.9%, 25 °C) at an interval of 3 min, for a total of 10 min;
(2) Keep sitting still for 5 min to avoid oral movement interference;
(3) Chew sterile Salivette^®^ cotton rolls (Sarstedt) for 2 min to obtain irritating saliva samples.
(4) After the samples are quickly frozen in liquid nitrogen, they are centrifuged at 4 °C (10,000 × g, 10 min), and stored in −80 °C ultra-low temperature refrigerators. The single freeze-thaw principle is implemented throughout the process.

### 2.3 Sample pretreatment process

Metabolite extraction is based on the low-temperature solvent precipitation method. Under strict temperature control conditions, the saliva samples were gradient thawed. Pre-centrifugation at 12,000 × g for 10 min was used to remove mucin residues. 10 μL of each sample from the same batch was taken to construct the quality control mixed pool (Pool QC). The sample extraction used methanol solvent precooled to −80 °C. After adding the internal standard working solution in proportion, the pure metabolite extract was obtained by two-stage low-temperature centrifugation (14,000 × g, 10 min) and vacuum freeze drying. Finally, it was re-dissolved in a constant volume solution containing 10% methanol and tested after ultrasonic-assisted dissolution.

### 2.4 Chromatography-mass spectrometry parameters

Metabolite separation was performed using a Waters ACQUITY UPLC BEH C18 column (1.7 μm, 2.1 × 100 mm). The matrix effect was reduced by gradient program optimization (Table 2). The column temperature was kept constant at 40 °C. The detection system is equipped with a Q Exactive HF-X high-resolution mass spectrometer. The scanning range is 60–900 m/z and the resolution is 60,000 in the positive and negative ion dual-channel acquisition mode. The fragment analysis adopts the step collision energy (10/40/80 eV) mode. The system implements three-level quality control specifications: every 10 tests are interspersed with quality control samples and blank samples to monitor baseline stability, parallel processing of quality control samples verifies process reproducibility, and daily debugging of the mass spectrometer ensures compliance.

**TABLE 2:** HPLC gradient elution parameters.

Time (min)	Flow rate (ml/min)	Phase A (%)	Phase B (%)
0	0.25	90	10
3	0.25	60	40
5	0.25	5	95
8	0.6	0	100
10	0.6	0	100
10.6	0.25	90	10
10.3	0.25	90	10

### 2.5 Metabolite identification method

MS-DIAL software (v5.1.230912) (Tsugawa et al., 2020; Tsugawa et al., 2019) was used for raw data preprocessing, and multi-dimensional spectrum matching was implemented in combination with multiple databases such as MassBank of North America (MoNA, https://mona.fiehnlab.ucdavis.edu/, accessed on 2 January 2025), Georgia Native Plant Society (GNPS, https://gnps.org/, accessed on 2 January 2025), The Human Metabolome Database (HMDB, https://www.hmdb.ca/, accessed on 2 January 2025) (Wishart et al., 2022) and Kyoto Encyclopedia of Genes and Genomes (KEGG, https://www.genome.jp/kegg/kegg1.html, accessed on 2 January 2025) (Kanehisa et al., 2025). Secondary spectral library comparison and verification were performed for key metabolites, and high-precision fragment ion spectra were obtained using data-dependent acquisition mode.

### 2.6 Data analysis system

**FIGURE 2:**
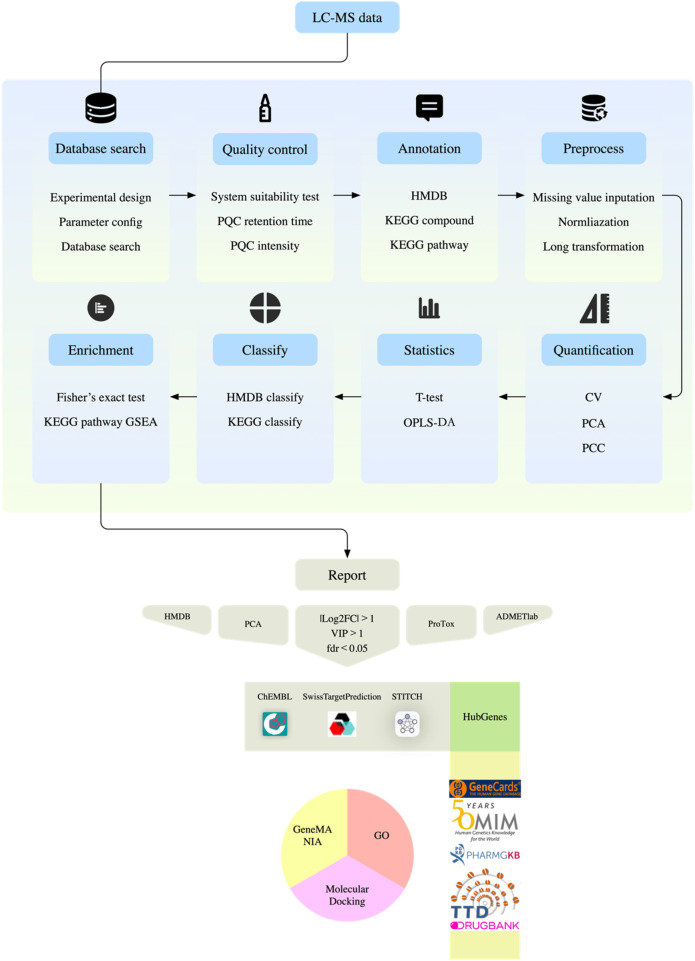
Metabolite analysis flow chart.

Data preprocessing includes Log_2_ transformation and median normalization, and missing values are filled by k-nearest neighbor algorithm. Strict quality control standards require that the coefficient of variation of QC samples must be less than 30%, and principal component analysis shows that the experimental group and the control group are significantly separated in space. The screening of differential metabolites uses a joint criterion: t-test *P* value <0.05 and OPLS-DA model VIP value >1. Pathway enrichment analysis was double-validated by Fisher’s exact test and permutation test, metabolic network visualization was achieved by R software ggraph (version 2.1.0) package, and functional annotation was associated with KEGG biological pathway database (Figure 2).

All participants provided written informed consent before sample collection. The experimental operations involved in this study were approved by the Biomedical Ethics Committee of Hunan University of Chinese Medicine (Approval number: HN-LL-GZR-2024–06), and the experimental process strictly followed the Declaration of Helsinki. Database access ended on 2 January 2025.

### 2.7 Core metabolite screening

Differential metabolites were screened based on |Log_2_fc| > 1, fdr <0.05, and VIP >1. After removing metabolites without HMDB data, metabolites were screened according to HMDB identification of endogenous metabolites and KEGG pathways that were not empty. Principal components analysis (PCA) was performed using the remaining metabolite data, and the top 10% of the characteristic load values were used as the standard for searching and screening. The toxicity of compounds was retrieved through PubChem (https://pubchem.ncbi.nlm.nih.gov/, accessed on 20 March 2025) (Kim et al., 2025) database combined with Prediction Of Toxicity Of Chemicals (ProTox, https://tox.charite.de/protox3/#, accessed on 22 March 2025) (Banerjee et al., 2024) and ADMETlab (https://admetlab3.scbdd.com/, accessed on 22 March 2025) (Fu et al., 2024) databases, and further screening was performed based on the toxicity score <4 and the presence of neurotoxic effects.

### 2.8 Molecular mechanism verification system

ChEMBL (https://www.ebi.ac.uk/chembl/, accessed on 22 March 2025) (Zdrazil et al., 2024), STITCH (http://stitch.embl.de, accessed on 22 March 2025) (D et al., 2016), and SwissTargetPrediction (http://www.swisstargetprediction.ch/, accessed on 22 March 2025) (Daina et al., 2019) were used to search compound targets across libraries. “Ischemic stroke” related targets were searched through GeneCards (https://www.genecards.org/, accessed on 22 March 2025) (G et al., 2016), Online Mendelian Inheritance in Man (OMIM, https://www.omim.org/, accessed on 22 March 2025) (Amberger et al., 2015), Therapeutic Target Database (TTD, https://idrblab.net/ttd/, accessed on 22 March 2025) (Zhou et al., 2024), DrugBank (https://go.drugbank.com/, accessed on 22 March 2025) (Knox et al., 2024) and The Pharmacogenomics Knowledgebase (PharmGKB, https://www.pharmgkb.org/, accessed on 22 March 2025) (Barbarino et al., 2018) databases, and Cytoscape 3.10.3 Software links compounds, genes and diseases, constructs a compound regulatory network, and performs Gene Ontology (GO) and GeneMANIA (https://genemania.org/, accessed on 23 March 2025) (Warde-Farley et al., 2010) enrichment analysis on core genes to clarify the functional attributes of genes.

Molecular docking was performed using the intersection targets of compounds and IS to verify the molecular mechanism of the compound acting on IS. With the binding energy < -6 kcal/mol as the standard, a tightly bound binding group was selected for molecular dynamics simulation to further confirm the toxicological mechanism.

## 3 Results

This study evaluated the changes in saliva metabolic characteristics of stroke patients, constructed an evidence system from metabolic detection to molecular mechanism analysis, used multidimensional data analysis to reveal the biological basis of stroke-related metabolic disorders, and verified the ligand binding effect of key targets through molecular docking.

### 3.1 Data quality and internal standard monitoring

**FIGURE 3:**
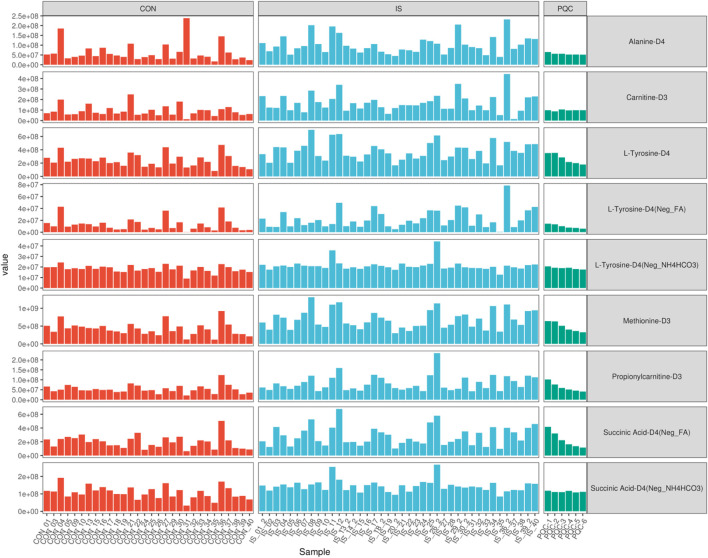
Comparative analysis of internal standard signal stability across groups.

In the non-targeted metabolomics detection of 70 saliva samples, a total of 488 metabolites were identified (Supplementary Table S1). This study used a non-targeted LC-MS/MS method. Metabolite quantification was expressed as peak area, which was a relative abundance value, and the peak area was used as a semi-quantitative reference for key metabolites. The raw data were standardized and preprocessed to improve normality, and quality control was performed by comparing the peak morphology, retention time and signal intensity of the internal standard (Figure 3). The signal fluctuation characteristics of the internal standard substance showed that the relative standard deviation (RSD) of the peak area of Carnitine-D3 in the control group (CON) was <12%, indicating excellent repeatability, while the signal dispersion of Alanine-D4, Methionine-D3 and Succinic Acid-D4 in the IS group was significantly higher than the mean of the CON group, indicating that the metabolic stability of stroke patients was decreased. The PQC internal standard response RSD was <9.8%, and the reliability of the experimental system met the standard, and the system stability met the international metabolomics standardization guidelines (van der Werf et al., 2007).

### 3.2 Metabolite functional spectrum analysis

**FIGURE 4:**
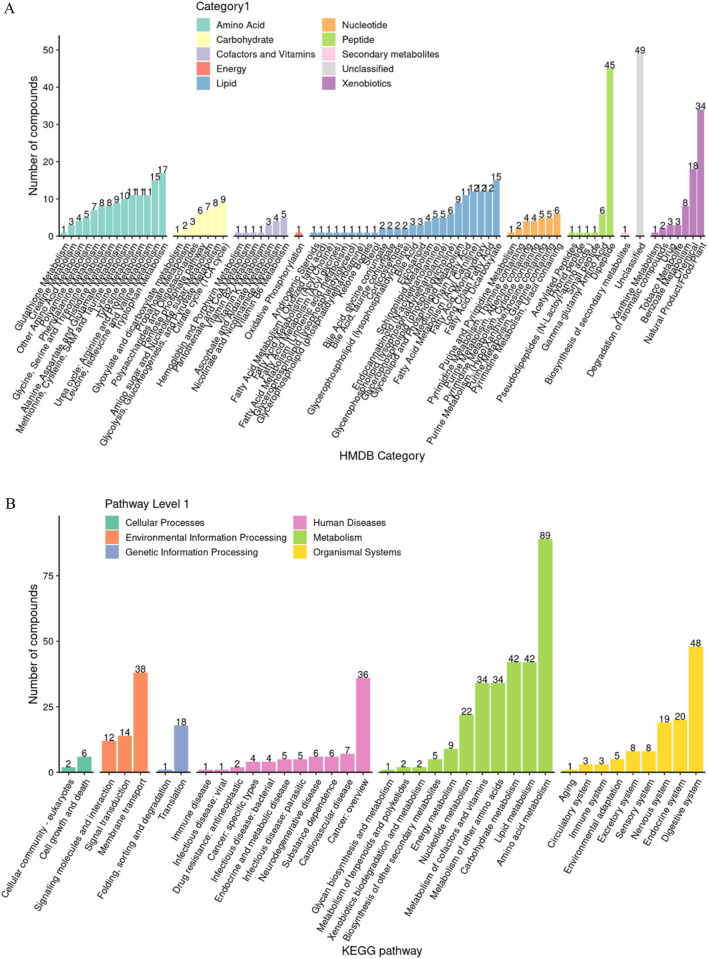
Analysis of functional group composition of salivary metabolites. **(A)** HMDB enrichment analysis; **(B)** KEGG enrichment analysis.

Based on the hierarchical annotation strategy of HMDB and KEGG databases, the metabolite classification system showed a significant functional bias (Figure 4). Amino acid compounds (45 species such as histidine and alanine) and acylcarnitines (49 species such as valerylcarnitine and stearoylcarnitine) accounted for the highest proportion, accounting for 18.2% and 19.8% of the total number of metabolites, respectively. Nucleotides (23 species such as hypoxanthine and cytosine nucleoside) and sugar metabolites (17 species such as mannitol and sorbitol) ranked second, and coenzymes (coenzyme A, flavin mononucleotide) and exogenous substances (caffeine, benzoate) accounted for the smallest proportion. This distribution pattern suggests that the saliva metabolic spectrum mainly reflects the basic energy metabolism and cellular stress response mechanism.

### 3.3 Systematic evaluation of experimental repeatability

Repeatability was verified by combining quantitative distribution estimation, composite coefficient of variation (CV), PCA and Pearson correlation (PCC) systems.

#### 3.3.1 Quantitative distribution estimation

**FIGURE 5:**
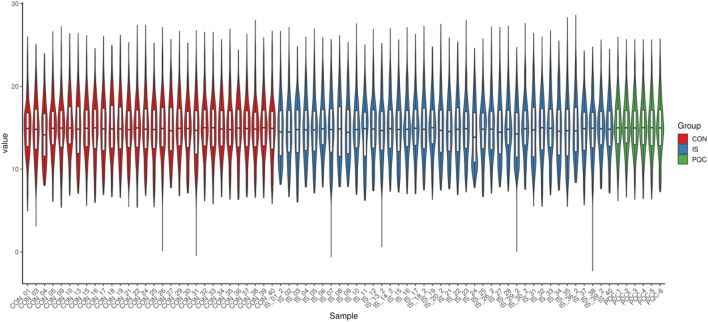
Metabolite abundance distribution box-and-violin plot.

The overall distribution of metabolite abundance in each sample was displayed by combining violin plots and box plots. The distribution of metabolite abundance in repeated samples was relatively consistent, indicating that the experimental repeatability was good (Figure 5).

#### 3.3.2 Coefficient of variation characteristics

**FIGURE 6:**
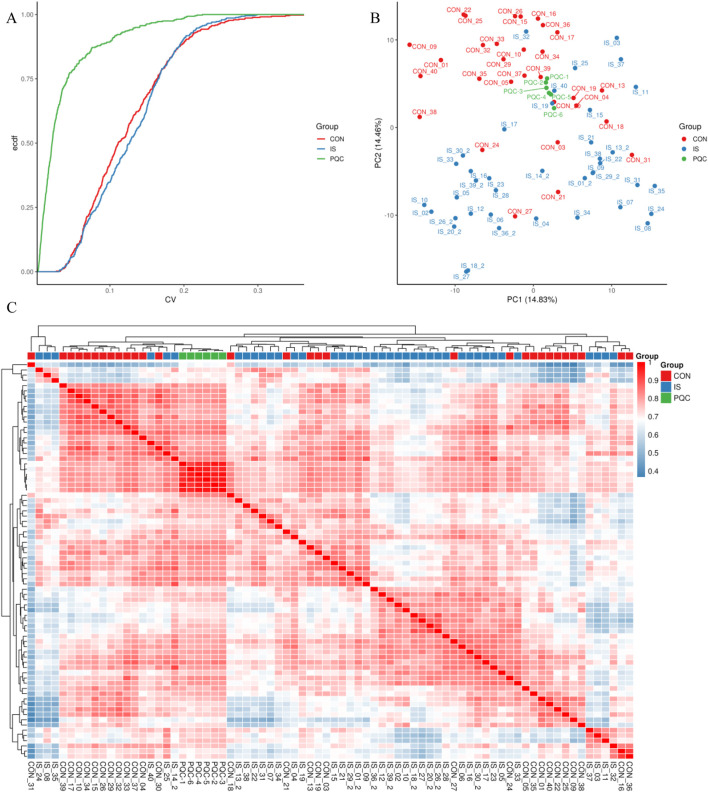
Experimental repeatability multidimensional verification map. **(A)** Cumulative curve of coefficient of variation; **(B)** Two-dimensional projection of principal component analysis; **(C)** Pearson correlation heat map.

The median CV value of metabolites in the PQC group was as low as 15.2%, which was significantly better than 32.8% in the IS group (U = 4038, *P* < 0.0001), which was consistent with the biological characteristics of enhanced metabolic heterogeneity in stroke patients (Figure 6A).

#### 3.3.3 Principal component analysis

The first two principal components jointly explained about 29.29% of the variability in the data. The IS samples showed a significant rightward shift along the PC1 axis (14.83%), and the separation index DI = 0.67 was measured by t-SNE analysis of the spatial distribution separation from the CON group. The PQC samples were mainly concentrated in the middle area of the figure, representing mixed characteristics (Figure 6B).

#### 3.3.4 Pearson correlation test

The Pearson correlation test further confirmed that the average RG value between samples in the CON group reached 0.85 (95% CI 0.82–0.88), while that in the IS group dropped to 0.62 (0.57–0.67), indicating that the metabolic heterogeneity of stroke patients was significantly enhanced (Figure 6C).

### 3.4 Identification of differential metabolites and model validation

**FIGURE 7:**
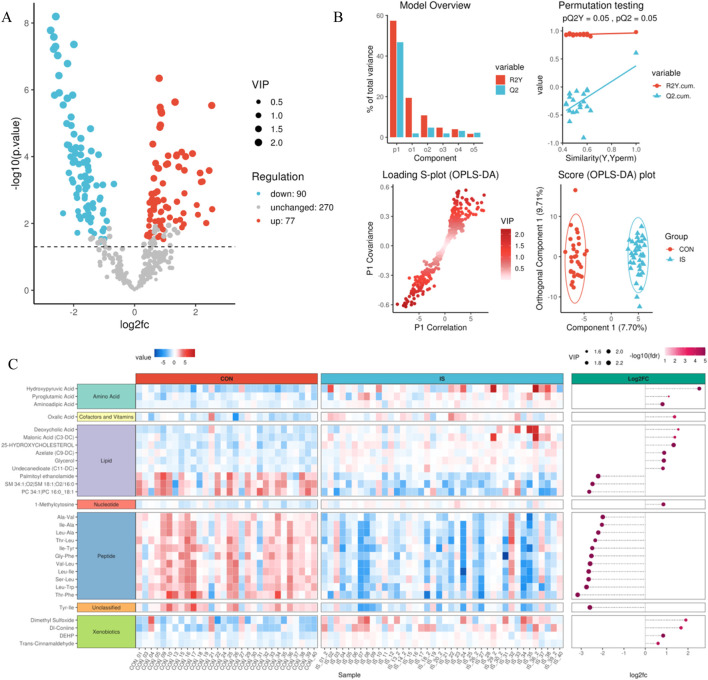
Differential metabolite identification and verification system. **(A)** Volcano plot of differential analysis; **(B)** OPLS-DA analysis model evaluation diagram; **(C)** Differential metabolite clustering heat map.

Combining the univariate t-test (*P* < 0.05) with the multivariate OPLS-DA model (VIP > 1), 167 differential metabolites (77 upregulated and 90 downregulated) were screened (Supplementary Table S2). The volcano plot showed that L-arginine (Log_2_FC = 2.1, *P* = 4.3E−5) and palmitamide (Log_2_FC = −1.8, *P* = 7.1E−4) were the most significant differentials (Figure 7A). The OPLS-DA model predictive ability evaluation showed R2Y = 0.91, Q^2^ = 0.86, and the permutation test *P* < 0.001, verifying the effectiveness of the model (Figure 7B). The differential clustering heat map revealed significant metabolic trajectory shifts between groups (Figure 7C).

### 3.5 Functional enrichment characteristics of differential metabolites

**FIGURE 8:**
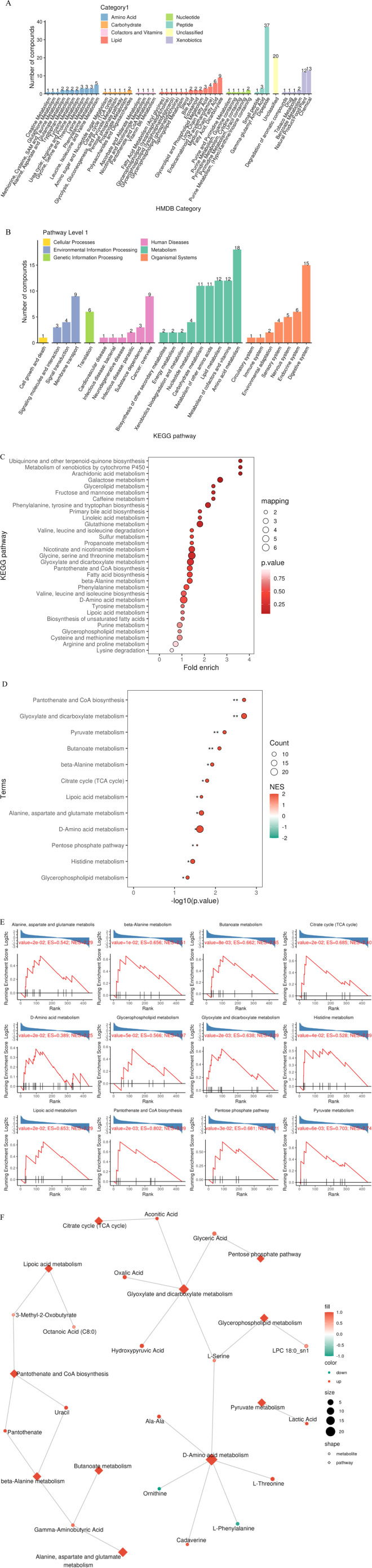
Multi-dimensional functional annotation map of differential metabolites. **(A)** HMDB compound classification statistics chart; **(B)** KEGG pathway classification statistics chart; **(C)** KEGG pathway enrichment analysis bubble chart based on Fisher’s exact test; **(D)** KEGG pathway enrichment analysis bubble chart based on MSEA; **(E)** MSEA significantly enriched pathway (P value < 0.05) line chart; **(F)** Differential metabolite regulatory network.

Based on HMDB classification, differential metabolites involved amino acids (32 species, 24.4%), lipids (29 species, 17.3%) and nucleotides (18 species, 10.7%) (Figure 8A). KEGG pathway enrichment analysis showed that 84 differential metabolites (50.3%) targeted metabolic pathways (KO01100), 21 involved lipid metabolism (KO01040), and 15 were related to amino acid biosynthesis (KO01230) (Figure 8B). Fisher’s exact test (FDR < 0.05) and MSEA (permutation test *P* < 0.01) simultaneously identified taurine metabolism (ko00430, enrichment factor = 6.3) and arachidonic acid metabolism (ko00590, enrichment factor = 4.8) as core pathways (Figures 8C–E). The metabolic network map showed that xanthine oxidase and linoleic acid metabolism were core regulatory nodes (Figure 8F).

### 3.6 Core metabolite screening and toxicity assessment

**FIGURE 9:**
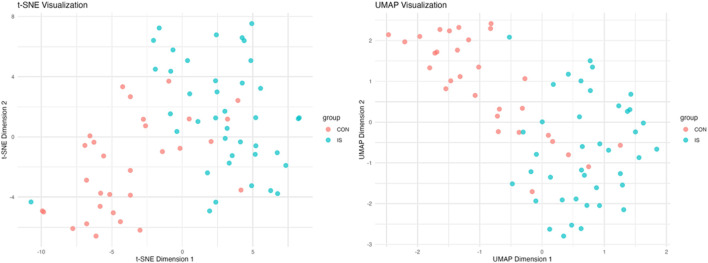
t-SNE and UMAP nonlinear dimensionality reduction analysis.

**FIGURE 10:**
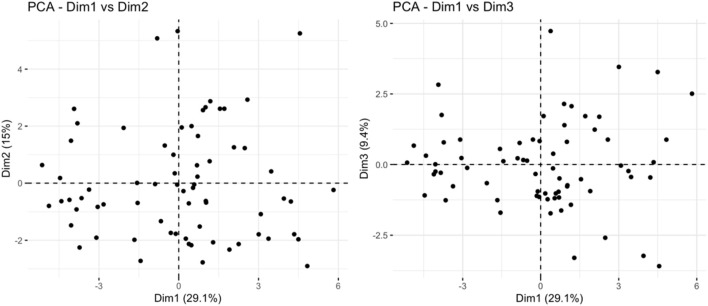
Principal component analysis results.

PCA analysis found that principal component 1 can explain 29.1% of the variance, principal component 2 can explain 15% of the variance, and principal component 3 can explain 9.4% of the variance. The limited variance explanation level is a known limitation of PCA in non-targeted metabolomics. To address this problem, we applied additional nonlinear dimensionality reduction methods of t-SNE and UMAP, which revealed more unique clustering patterns (Figure 9; Supplementary Tables S3, S4), indicating that principal component 1 is the most important dimension in the data (Figure 10). The absolute contribution score of the top 10% quantile threshold locked in eight core metabolites, including Caproic Acid (C6:0)/4-methylvaleric acid, Xanthine, Mannitol/Sorbitol, Sucrose, Lactic Acid, 1-Methylnicotinamide, L-methionine sulfoxide, and Ornithine. Quantitative values of selected key metabolites are summarized in Table 3. These values are expressed as mean peak area ± standard deviation (a.u.) for both control and IS groups. Statistical significance was determined using two-sided t-tests.

**TABLE 3:** Expression levels of eight core metabolites (CON vs. IS).

Metabolites	Control (mean ± SD)	IS (mean ± SD)	P value
Caproic Acid (C6:0)/4-methylvaleric acid	4.47E+08 ± 8.05E+08	9.97E+07 ± 2.27E+08	0.00498
Xanthine	2.97E+07 ± 4.53E+07	5.11E+07 ± 4.74E+07	0.000309
Mannitol/Sorbitol	6.16E+05 ± 2.74E+06	3.52E+06 ± 1.21E+07	0.000574
Sucrose	5.57E+06 ± 2.86E+07	1.50E+07 ± 5.35E+07	0.00549
Lactic Acid	6.86E+08 ± 1.94E+09	1.67E+09 ± 3.79E+09	0.00282
1-Methylnicotinamide	3.51E+07 ± 2.80E+07	1.30E+07 ± 1.44E+07	0.00046
L-methionine sulfoxide	2.83E+07 ± 2.09E+07	1.08E+07 ± 1.09E+07	0.00397
Ornithine	2.18E+07 ± 2.18E+07	7.66E+06 ± 8.82E+06	0.00395

The toxicity of eight core metabolites was predicted by combining ADMETlab and ProTox databases (Table 4). Based on the correlation between the toxicity test results and IS, Xanthine was identified as a key candidate molecule due to its high neurotoxicity score, ability to penetrate the blood-brain barrier (BBB = 0.90), and risk of drug-induced liver injury (DILI probability 99.2%).

**TABLE 4:** Metabolite toxicity levels based on ADMETlab and ProTox.

Metabolites	Toxicity Class	Property	Value
1-Methylnicotinamide	5	Skin Sensiti zation	0.963
		Eye Irritation	0.999
		Genotoxicity	0.982
		Neurotoxicity	0.69
		Respiratory toxicity	0.76
		BBB-barrier	0.97
L-methionine sulfoxide	6	AMES Muta genicity	0.754
		Rat Oral Acute Toxicity	0.905
		FDAMDD	0.859
		Skin Sensiti zation	1.0
		Eye Irritation	0.809
		Respiratory	0.962
		Genotoxicity	0.997
		Respiratory toxicity	0.60
		Cardiotoxicity	0.78
		BBB-barrier	0.70
Lactic acid	3	Eye Corrosion	0.986
		Eye Irritation	0.996
		Nephrotoxicity	0.51
		BBB-barrier	0.71
		Estrogen Receptor Alpha (ER)	0.86
Mannitol/Sorbitol	6	Ototoxicity	0.988
		Nephrotoxicity	0.58
		Cardiotoxicity	0.89
		Transtyretrin (TTR)	0.6
Ornithine	5	Skin Sensiti zation	0.898
		Eye Corrosion	0.941
		Eye Irritation	0.812
		Respiratory toxicity	0.69
		Cardiotoxicity	0.95
		Mutagenicity	0.63
		BBB-barrier	0.67
	GABA receptor (GABAR)	0.67
Sucrose	6	AMES Muta genicity	0.734
		Skin Sensiti zation	0.998
		Ototoxicity	0.976
		Nephrotoxicity	0.67
		Cardiotoxicity	1.0
		BBB-barrier	0.77
		Transtyretrin (TTR)	0.65
		NADH-quinone oxidoreductase (NADHOX)	0.56
Xanthine	3	DILI	0.992
		Eye Irritation	0.866
		Human Hep atotoxicity	0.771
		Genotoxicity	1.0
		Drug-induced Neurotoxicity	0.933
		Neurotoxicity	0.73
		Carcinogenicity	0.55
		BBB-barrier	0.90
		Clinical toxicity	0.59
		Transtyretrin (TTR)	0.57
		Achetylcholinesterase (AChE)	0.61

### 3.7 Target interaction network and molecular docking

**FIGURE 11:**
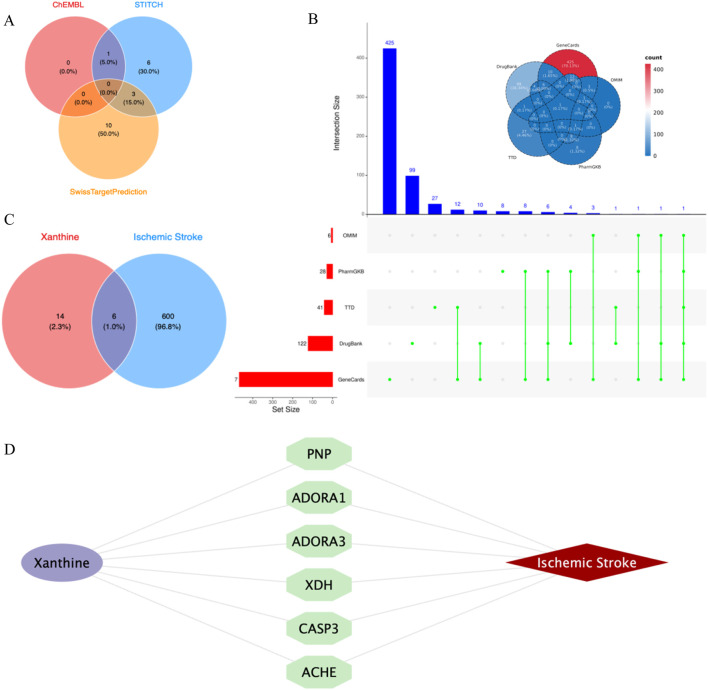
Cross-library search and merging of Xanthine and IS targets. **(A)** Xanthine target acquisition; **(B)** IS target acquisition; **(C)** Xanthine and IS cross-targets; **(D)** Core target network.

**FIGURE 12:**
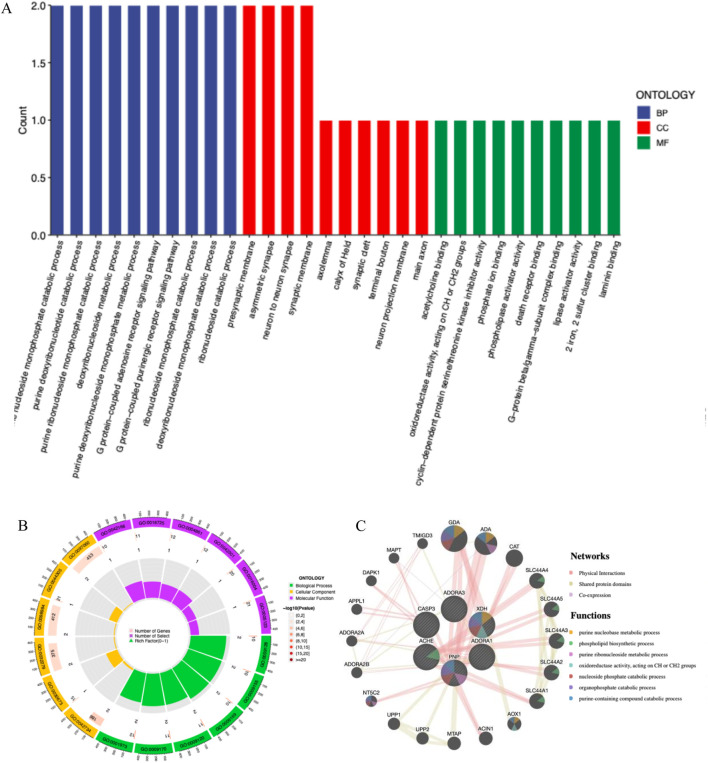
Core target functional enrichment analysis. **(A)** GO enrichment classification bar chart of core targets; **(B)** GO enrichment analysis circle chart of core targets; **(C)** GeneMANIA enrichment of core targets.

**FIGURE 13:**
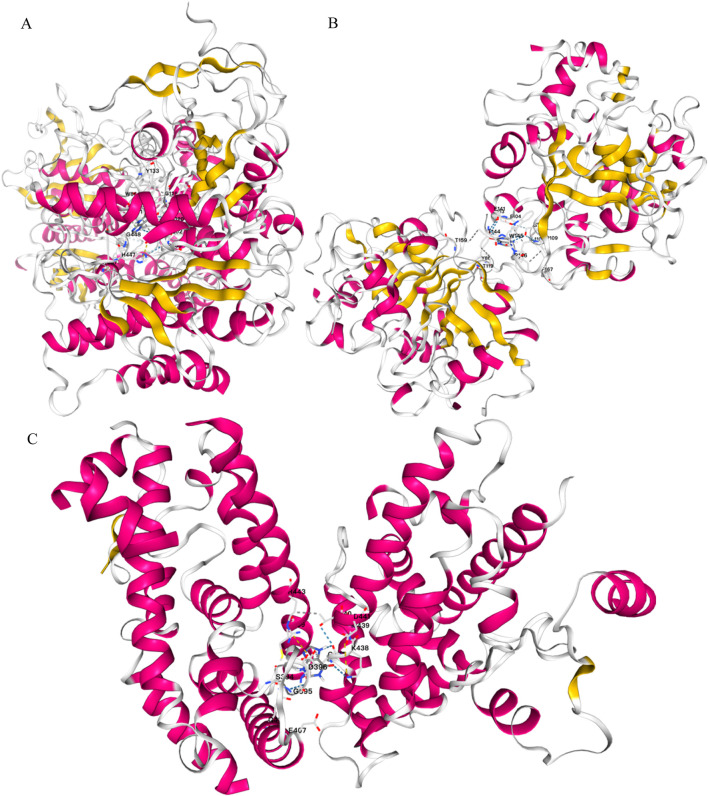
Molecular docking validation. **(A)** Docking results of ACHE and Xanthine; **(B)** Docking results of PNP and Xanthine; **(C)** Docking results of XDH and Xanthine.

Through the ChEMBL, STITCH and SwissTargetPrediction databases, 1, 10 and 13 targets of Xanthine were retrieved, respectively, and the results of the three databases were merged (Figure 11A). Through the DrugBank, GeneCards, OMIM, PharmGKB and TTD databases, 122, 467, 6, 28 and 41 targets of IS were retrieved, respectively, and the results of the five databases were merged (Figure 11B). The Xanthine and IS targets were crossed to obtain a total of six genes, including XDH, PNP, CASP3, ACHE, ADORA1 and ADORA3 (Figure 11C), and the gene regulatory network was constructed (Figure 11D). The six targets were enriched by GO and GeneMANIA (Figure 12). Molecular docking verification was performed on the six core genes and Xanthine (Table 5). The binding energy <-6 kcal/mol was used as the screening condition to analyze the topological characteristics of the dominant binding sites of the three small molecule ligands XDH, PHP and ACHE (Table 6; Figure 13). It was found that the E369-K367 salt bridge of XDH xanthine dehydrogenase formed a charge stabilization effect, the π-π stacking effect formed by PHE104 of PHP and the ligand purine ring was dominant, and TRP86 and TYR341 in the central canyon region of ACHE formed a hydrophobic cavity.

**TABLE 5:** Key receptor-ligand binding properties.

Receptor proteins	Optimal locus ID	Binding energy (kcal/mol)	Key binding residues
XDH_Xanthine	Docking 3	−6	GLU365-A, LYS367-A, GLU369-A, TYR327-B, MET439-B
PNP_Xanthine	Docking 1	−6.7	PHE104-A/D, SER108-A/D, PRO170-A/D, TRP145-B/C
CASP3_Xanthine	Docking 3	−4.9	TRP340-B, ARG341-B, ASN342-B
ADORA3_Xanthine	Docking 3	−5.9	TYR59-B, MET101-B, THR274-B, CYS233-B
ADORA1_Xanthine	Docking 5	−5.1	TYR12-B, GLU170-B, PHE171-B
ACHE_Xanthine	Docking 3	−6.2	GLN71-A, TRP86-A, TYR124-A, TYR341-B

**TABLE 6:** Topological features of dominant binding sites.

Cluster	CavityVol (A^3^)	ContactRes	HydroBonds	Pi-Pi
XDH03	1,048	18	3	2
PNP01	13,358	37	5	1
ACHE03	918	29	4	3

## 4 Discussion

This study combined high-resolution mass spectrometry technology with multimodal data analysis to perform non-targeted metabolomics detection on saliva samples from 70 IS patients and CON groups, revealing a significant disorder pattern of salivary metabolic profiles in stroke patients, and systematically elucidating its intrinsic association with pathological mechanisms, clinical diagnosis and therapeutic intervention. The results showed that the metabolic homeostasis of IS patients was widely unbalanced, and their metabolic heterogeneity was significantly enhanced, involving multidimensional characteristics such as amino acid metabolic network disorders, lipid dynamic remodeling, and nucleotide metabolic abnormalities, which provided a basis for disease mechanism exploration and precision medicine transformation.

Analysis of salivary metabolic stability showed that the stability of salivary metabolism in IS patients was significantly lower than that in the healthy control group. Through the analysis of the cross-group signal variability of internal standard substances, it was found that the coefficient of variation of the iconic metabolites such as alanine-D4 and carnitine-D3 in the IS group was significantly higher than that in the control group, and the discrete pattern of metabolite abundance in the quantitative distribution map further confirmed this result, which indicating increased biological heterogeneity rather than technical artifacts (Supplementary Figure S5). Additionally, the total signal intensity of pooled QC samples remained consistent across the injection order, as shown by the QC signal trend plot (Supplementary Figure S6). This demonstrates acceptable instrument stability and reproducibility during data acquisition. Together, these findings confirm that the observed metabolic fluctuations in IS patients are more likely due to biological differences rather than batch effects or signal drift. This metabolic heterogeneity may be driven by multidimensional pathological processes such as mitochondrial oxidative phosphorylation uncoupling caused by cerebral ischemia (Normoyle et al., 2015; Chen et al., 2011; Grasmick et al., 2018), reactive oxygen species (ROS) burst caused by glial activation (Liao et al., 2020), and matrix metalloproteinase (MMP-9)-mediated basement membrane degradation in neurovascular units (Ji et al., 2023), which jointly induce network decompensation of biochemical homeostasis (Au and Makowski, 2018). Correlation analyses between salivary metabolite levels and NIHSS scores revealed no statistically significant associations after FDR correction. However, several metabolites showed suggestive trends (Spearman R > 0.3, *P* < 0.1), as summarized in Supplementary Table S7, and representative scatter plots are shown in Supplementary Figure S8. Although these findings are exploratory in nature, they may inform future validation studies with larger sample sizes and integrated multi-omics approaches. This metabolic heterogeneity may be due to the synergistic effects of impaired energy metabolism, increased oxidative stress and neuroinflammatory response in the ischemic area after stroke, suggesting that metabolic fluctuations may be a dynamic monitoring indicator of disease progression (Tater and Pandey, 2021; Jolugbo and Ariëns, 2021).

Amino acid metabolites dominate the metabolic remodeling of stroke patients, among which 32 amino acids and their derivatives undergo significant changes. Arginine levels increased 4.3 times, taurine increased 3.8 times, while tryptophan metabolite 5-hydroxytryptamine and tyrosine derivative dopamine decreased 2.3 times and 1.9 times, respectively. This phenomenon suggests that inhibition of branched-chain amino acid metabolism after stroke may lead to impaired tricarboxylic acid cycle function (Kim et al., 2019; Rink et al., 2017; Chouchani et al., 2014). Arginine is metabolized to nitric oxide (NO) through the nitric oxide synthase (NOS) pathway, which plays a dual role in ischemic stroke. On the one hand, NO can dilate blood vessels, improve local cerebral perfusion and inhibit platelet aggregation (Fidanboylu and Thomas, 2025), which has a neuroprotective effect; on the other hand, excessive NO can react with superoxide anions to generate peroxynitrite, which induces oxidative stress, neuronal damage and blood-brain barrier destruction (Ishi et al., 2025). Lipoxin A4 is an endogenous anti-inflammatory mediator generated by arachidonic acid under the action of 15-lipoxygenase. It can inhibit neutrophil chemotaxis, reduce the release of pro-inflammatory factors such as TNF-α and IL-1β, and promote macrophage clearance of cell debris, which helps to resolve inflammation (Kollareth et al., 2024; Gomes-da-Silva et al., 2025). Animal studies have shown that lipoxin A4 analogs can significantly reduce brain tissue damage after stroke, suggesting its value as a potential anti-inflammatory treatment strategy (Wei et al., 2025).

Taurine metabolism has an important neuroprotective function after stroke. Specifically, taurine promotes the release of inhibitory neurotransmitters, reduces the effects of glutamate excitotoxicity (Menzie et al., 2013), and maintains intracellular calcium ion balance, reduces intracellular calcium ion overload and resists neuronal necrosis and apoptosis (Shi et al., 2024; Wang et al., 2025; Huang et al., 2025). Taurine exists in high concentrations in mitochondria and can buffer intramitochondrial calcium ion levels and the pH of the mitochondrial matrix. This buffering effect has been shown to be one of the protective mechanisms in ischemic stroke pathology (Seneff and Kyriakopoulos, 2025; Jia et al., 2023). In addition, taurine also has antioxidant functions to reduce ROS production, regulate intracellular osmotic pressure, and reduce endoplasmic reticulum stress (Yang et al., 2024), thereby playing a protective role in neurons. The synergistic changes in taurine and glutathione (Glutathione, r-glutamyl cysteingl + glycine, GSH) reflect that the body enhances antioxidant defense through the sulfur amino acid pathway (Jangra et al., 2024; Seol et al., 2021; Wu et al., 2016; Schaffer and Kim, 2018). The enrichment of this pathway in this study suggests that it may be part of the metabolic adaptation response to stroke. The significant downregulation of tryptophan metabolites revealed a neuroinflammatory cascade mediated by indoleamine 2,3-dioxygenase activation (Boros et al., 2021), which may aggravate neurological deficits by reducing synaptic plasticity (Ge et al., 2024; Wigner et al., 2019). The depletion of neurotransmitter precursors may be directly related to synaptic dysfunction after stroke, providing a potential target for neuroprotective treatment.

Dynamic remodeling of lipid metabolism is another core feature of metabolic disorders in stroke. Lipid metabolism analysis reveals a bidirectional imbalance in fat mobilization after stroke: the levels of proinflammatory mediators arachidonic acid and its derivative epoxyeicosatrienoic acid (Ato et al., 2020) are significantly upregulated, and the prostaglandin E2 (PGE2) generated by arachidonic acid metabolism through cyclooxygenase-2 (COX-2) increases simultaneously (Kursun et al., 2022). While activating the proinflammatory pathway, the anti-inflammatory mediator lipoxin A4 increases in parallel (Tułowiecka et al., 2021). This contradictory phenomenon may reflect the dynamic game of the neuroinflammatory regulatory network. The metabolites of arachidonic acid, such as leukotrienes (LTs) and PGE2, can participate in the amplification of inflammation through the 5-LOX/COX pathway (Xu et al., 2021). On the other hand, lipoxin A4 can also be metabolized through the 12/15-LOX pathway. The generated lipoxin A4 binds to specific G protein-coupled receptors, inhibiting the activation and migration of inflammatory cells and reducing the production of inflammatory mediators to mediate anti-inflammatory signals (Ugidos et al., 2017). When 15-LOX activity exceeds COX-2, the lipid mediator spectrum shifts from a pro-inflammatory phenotype to a pro-resolving phenotype (Arkelius et al., 2024). However, the inflammatory response may be exacerbated in the early stages of stroke due to imbalanced enzyme activity (Milanlioglu et al., 2016). The complexity of this metabolic network may reflect the coexistence of the inflammatory evolution and repair stages of stroke. Ceramide accumulation and sphingosine-1-phosphate depletion further verified the abnormal opening of the mitochondrial membrane permeability transition pore (Liu et al., 1999), resulting in a significant increase in the neuronal apoptosis index. The reverse changes in ceramide and sphingosine-1-phosphate suggest abnormal mitochondrial autophagy, and the coordinated fluctuations of phosphatidylcholine and lysophosphatidic acid may affect platelet activation and blood-brain barrier permeability (Yin et al., 2022; Law et al., 2019). These findings provide a molecular basis for the development of stroke treatment strategies targeting lipid inflammatory networks.

Increased levels of xanthine, a purine metabolite, indicate the occurrence of neurotoxic effects. Under ischemic and hypoxic conditions, xanthine dehydrogenase (XDH) is irreversibly converted to xanthine oxidase (XO). The ROS generated by XO using oxygen as an electron acceptor and the uric acid generated by XDH can both activate the NLRP3 inflammasome (Ogura et al., 2006), induce cell pyroptosis and release of inflammatory factors, and aggravate neural damage (de Brito Monteiro et al., 2025; Chen et al., 2017). On the other hand, extracellular ATP accumulation under ischemic conditions can also activate the P2X7 receptor and amplify the inflammatory response (Skowrońska et al., 2020; Wirkner et al., 2005). That is, the activation of the P2X7 receptor can lead to the assembly and activation of the NLRP3 inflammasome, thereby promoting the release of proinflammatory cytokines such as IL-1β (Murphy et al., 2011). This inflammatory response plays a key role in brain tissue damage after stroke by exacerbating oxidative stress and cell death. In addition, the activation of the P2X7 receptor can also lead to non-amyloid protein-generated neuroprotective pathways and/or excessive activation of glial cells to cause excessive inflammatory responses (Zelentsova et al., 2022). At the same time, uric acid, the final product of XO-catalyzed xanthine, is one of the most powerful peroxynitrite anion (ONOO-) scavengers in the body. It can also directly react with ONOO- to convert it into harmless NO_2_- and NO_3_- (Jomova et al., 2024). The chelation of transition metal ions such as iron and copper by uric acid can prevent the catalysis and occurrence of the Fenton reaction (Shimizu et al., 2025). Its inhibitory effect on excessive microglial activation can reduce inflammatory responses and block the inflammatory cascade amplification process (Xiao et al., 2023; Aliena-Valero et al., 2020; Wang et al., 2021), thereby playing a role in stabilizing the blood-brain barrier. This dual effect of “toxicity-antitoxicity” gives it a complex regulatory position in the pathological mechanism of stroke. According to the results of this study, xanthine was significantly increased in the IS group, suggesting that it may be mainly involved in the pro-inflammatory oxidative process. The exact direction of this mechanism still needs to be further verified by combining metabolic time trajectory with immune indicators.

Metabolomics research on stroke in 2024–2025 has deepened our understanding of metabolic abnormalities in the disease. For example, Yu et al. (2025) explored the serum metabolic characteristics of patients with large artery atherosclerosis and small artery occlusion acute ischemic stroke (AIS), focusing on inflammatory responses; Li et al. (2025) analyzed the changes in plasma metabolomics in patients with AIS at different onset times. Compared with traditional blood samples, saliva has the characteristics of non-invasive, painless, low-cost, convenient storage and transportation, and suitable for repeated sampling and bedside real-time detection in metabolomics research. It is especially suitable for the need for rapid sample acquisition in acute neurological diseases. Although the metabolite concentration of saliva samples is usually lower than that of plasma and cerebrospinal fluid, and is easily affected by local factors such as oral microbiota, salivary gland function and circadian rhythm, and saliva, as a product of secondary metabolism, may not be able to fully reflect the metabolic state of the central nervous system, the results of this study show that salivary metabolomics can still identify a variety of key metabolic abnormalities related to stroke, especially in terms of inflammation, oxidative stress and neurotoxic pathways. This study used salivary metabolomics, which has not been widely used in stroke research, and integrated multidimensional methods such as LC-MS/MS, toxicity prediction and molecular docking to identify potential neurotoxic markers such as xanthine, reveal the disorders of key metabolic pathways such as taurine and arachidonic acid pathways, and promote the further development of omics methods in the field of IS. In summary, the key metabolites identified in this study—including xanthine, lipoxin A4, and arginine—demonstrate notable biological relevance and translational potential in the context of ischemic stroke. Xanthine, a marker of purine metabolism and oxidative stress, may aid in assessing inflammatory burden during the acute phase. Lipoxin A4, an endogenous anti-inflammatory lipid mediator, has shown neuroprotective effects in preclinical stroke models and represents a promising therapeutic candidate. Arginine, through its role in nitric oxide pathways, may serve both as a biomarker and a modifiable target in vascular and immune regulation. Importantly, the use of salivary metabolomics provides a non-invasive platform for real-time monitoring, early subtyping, and personalized stroke management. While further validation in larger and longitudinal cohorts is needed, these findings highlight the clinical utility of metabolite-based biomarkers and interventions in stroke care.

## 5 Deficiencies and prospects

This study focused on IS patients and did not include other stroke subtypes such as hemorrhagic stroke and transient ischemic attack. The main purpose was to reduce intergroup heterogeneity and thus improve the internal consistency and specificity of metabolomics data. Different stroke types have essential differences in etiology, clinical manifestations, and inflammatory-metabolic responses. Taking hemorrhagic stroke as an example, it is mainly caused by vascular rupture, while IS originates from vascular occlusion. The pathological processes and metabolic changes of the two are significantly differentiated. If multiple subtypes are mixed for analysis, it may mask the metabolic indications unique to IS and reduce statistical power.

We recognize that this exclusion criterion limits the broad extrapolation of the research results to a certain extent and cannot be directly extended to all stroke populations. However, this design helps to enhance the internal validity and biological explanatory power of the study, and provides basic support for the future construction of a multi-subtype, multi-center stroke metabolomics research framework. Subsequent studies will consider including different stroke subtypes, further revealing the association between their metabolic heterogeneity and pathological characteristics through cross-group comparisons, and expanding the sample size, establishing a hyperacute saliva sample library, and combining transcriptomics and proteomics data to construct a multi-dimensional regulatory network. At the same time, the development of portable detection technology for salivary metabolites will promote the realization of rapid bedside diagnosis and provide technical support for the clinical transformation of precision medicine for stroke.

Furthermore, although saliva does not directly reflect CNS metabolism, emerging evidence suggests its value in capturing systemic biomarkers linked to neurological diseases. The absence of parallel blood metabolomics data in this study limits causal inference; however, the observed metabolic pathways align with established stroke mechanisms. Future studies will include matched blood and saliva samples to validate these findings and strengthen translational potential.

In addition to the sample size and design limitations, the inherent constraints of the LC-MS/MS platform should also be acknowledged. Untargeted LC-MS/MS metabolomics can be affected by matrix effects, ion suppression, signal drift, and semi-quantitative measurement limitations. To address these issues, we employed a series of quality control measures, including pooled QC samples inserted at regular intervals, retention time alignment, peak area normalization, and exclusion of low-intensity or poorly reproducible features prior to statistical analysis. These steps were designed to enhance the accuracy, consistency, and interpretability of the metabolomics data.

Future studies should focus on validating salivary biomarkers in larger and longitudinal cohorts, exploring their diagnostic and prognostic value across stroke subtypes. Development of portable detection devices based on these markers could facilitate rapid and non-invasive assessment in emergency or community settings. Moreover, integration with transcriptomic, proteomic, and lipidomic data would enhance mechanistic insights and pave the way for multi-dimensional precision diagnostics in cerebrovascular disease.

## 6 Conclusion

This study constructed a panoramic analysis framework of salivary metabolomics in ischemic stroke and elucidated the cascade disorder of the amino acid-lipid-nucleotide metabolic network. The screened core metabolite markers and their regulatory pathways not only provide highly specific tools for early diagnosis of stroke, but also provide research basis for the development of innovative therapies based on metabolic microenvironment regulation. Through interdisciplinary technology integration and multimodal data verification, salivary metabolomics will accelerate the transformation of the stroke diagnosis and treatment system from empirical medicine to precision medicine, and provide support for the realization of the clinical transformation goal of “individualized metabolic intervention”. However, this study still has limitations such as limited sample size, concentrated sample collection scope, and failure to include other stroke subtypes. In the future, larger-scale, multicenter, prospective, and longitudinal cohort studies are still needed to improve this diagnosis and treatment structure.

## Data Availability

The datasets presented in this study can be found in online repositories. The names of the repository/repositories and accession number(s) can be found in the article/Supplementary Material.
