# Supplementary figures and images for: Toxicological analysis of metabolites in ischemic stroke based on salivary metabolomics
Source: Front Mol Biosci. 2025 Aug 29;12:1609227. doi: 10.3389/fmolb.2025.1609227 (PMC12425714; doi:10.3389/fmolb.2025.1609227)

QC TIC stability


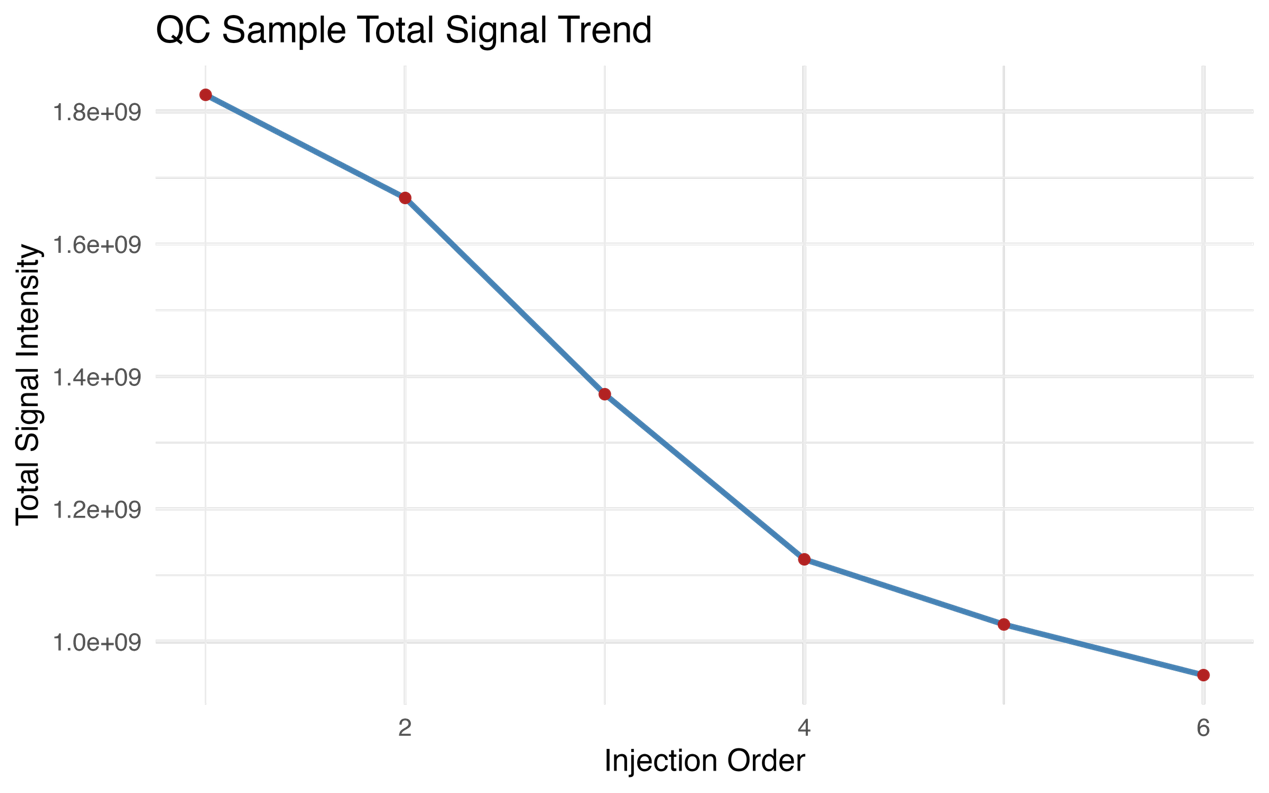

Supplement: Supplementary file 1 [file Supplementaryfile6.docx]

CV internal standards boxplot


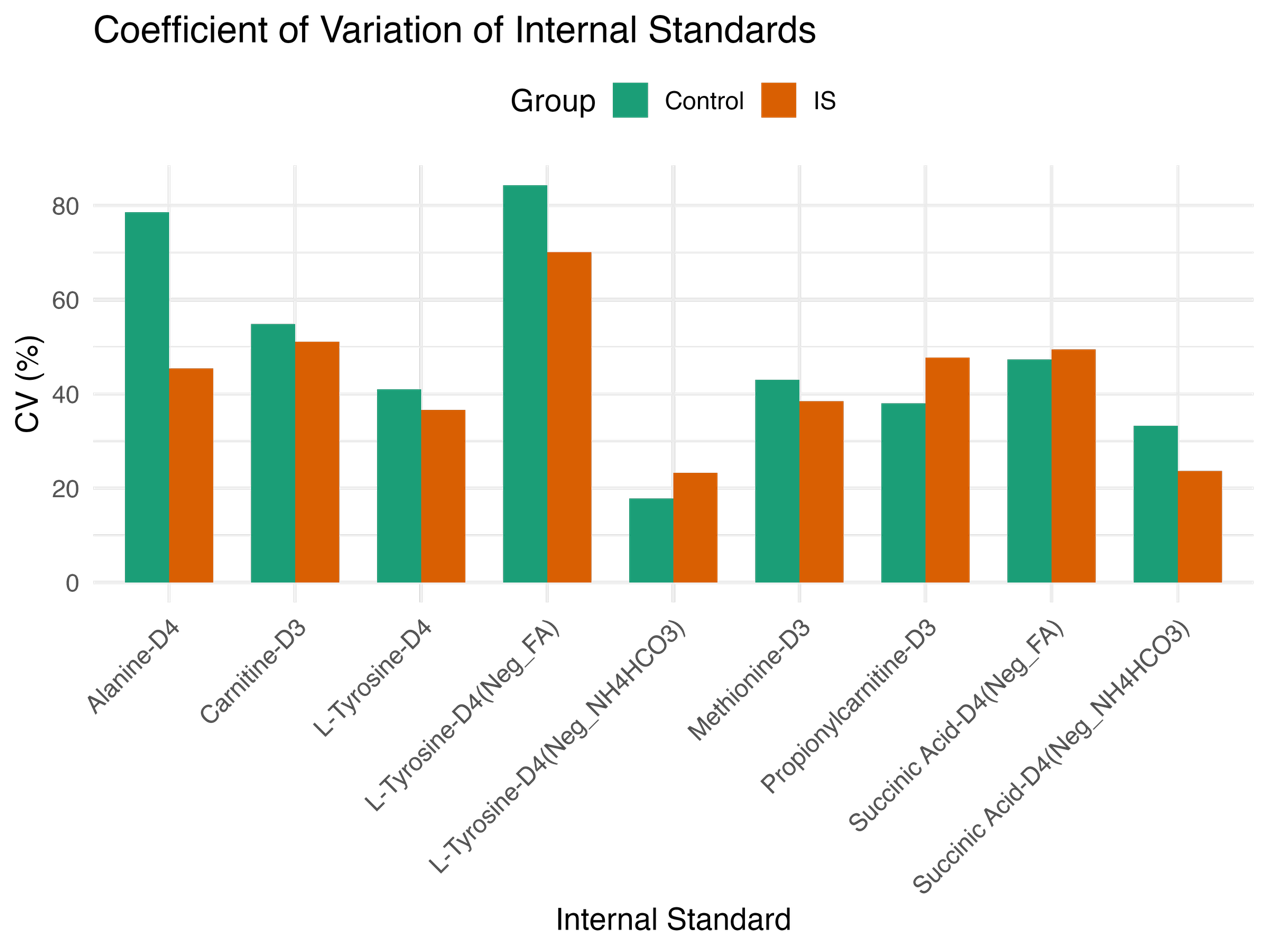

Supplement: Supplementary file 3 [file Supplementaryfile5.docx]

Scatter plots of top 5 metabolites vs. NIHSS


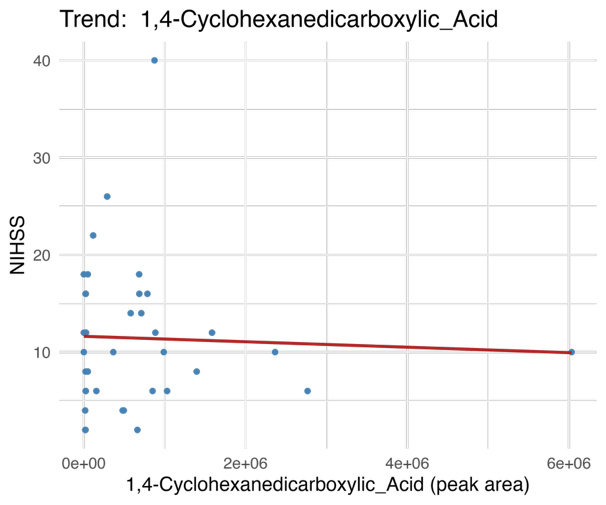

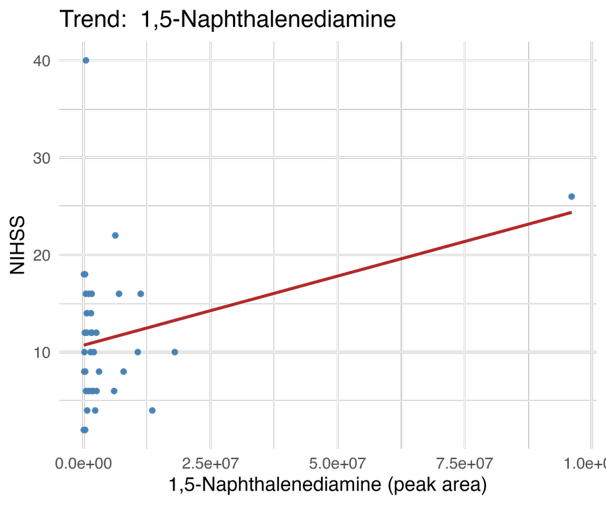


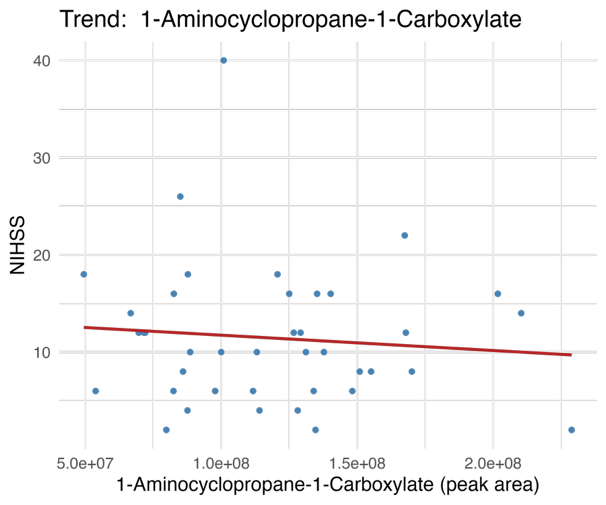

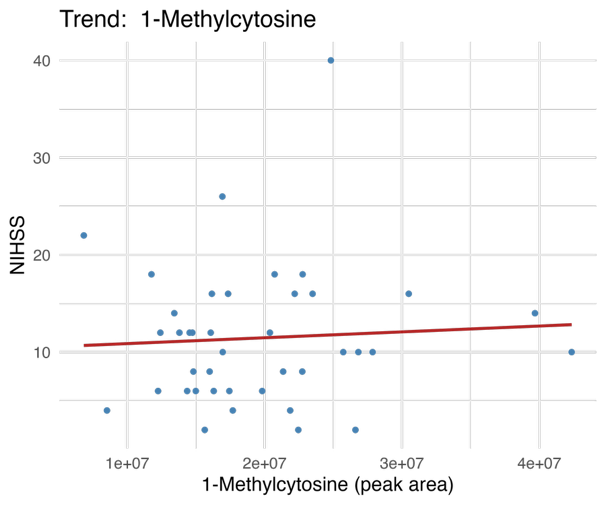


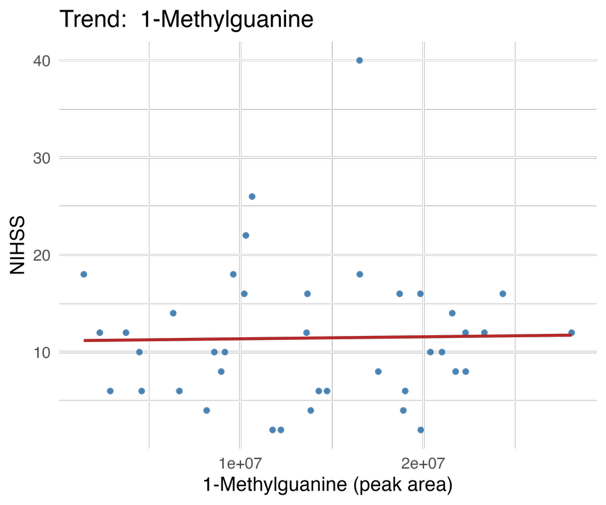

Supplement: Supplementary file 7 [file Supplementaryfile8.docx]
