# Supplementary material for: Toxicological analysis of metabolites in ischemic stroke based on salivary metabolomics
Source: Front Mol Biosci. 2025 Aug 29;12:1609227. doi: 10.3389/fmolb.2025.1609227 (PMC12425714; doi:10.3389/fmolb.2025.1609227)
Supplement: Supplementary file 4 [file Supplementaryfile3.docx]

t-SNE Results

| V1 | V2 | group |
| --- | --- | --- |
| -4.8923077 | -5.0387399 | CON |
| -0.1637152 | -0.7587581 | CON |
| 3.93299337 | 2.41775602 | CON |
| -3.6823194 | -3.8692831 | CON |
| -7.7599409 | -6.0847436 | CON |
| -6.9022643 | -0.5663892 | CON |
| -2.7602805 | 1.17065783 | CON |
| -5.788586 | -3.7378643 | CON |
| -9.913687 | -4.9138697 | CON |
| -6.545618 | 0.06993692 | CON |
| -3.683857 | 2.66904167 | CON |
| -2.6088742 | 0.73488271 | CON |
| 2.03547603 | -0.3068511 | CON |
| -6.1152447 | -6.5846569 | CON |
| 4.14964485 | -3.5302522 | CON |
| -6.776811 | -5.7678668 | CON |
| -6.7865091 | -2.8740301 | CON |
| 3.19806009 | 1.1824104 | CON |
| -5.5764565 | -1.265552 | CON |
| 0.48257789 | 1.19313092 | CON |
| -4.2282836 | 3.3314573 | CON |
| -5.1417351 | -3.8216107 | CON |
| -5.8252335 | -4.610675 | CON |
| -6.2638292 | -0.3546835 | CON |
| -3.686012 | -2.2260516 | CON |
| -9.826011 | -4.9854081 | CON |
| -0.9604584 | 3.70092543 | CON |
| -2.9980964 | -6.1920552 | CON |
| -1.4148545 | -0.9750979 | CON |
| -4.3889154 | -5.631186 | CON |
| 3.00947759 | 1.70190871 | IS |
| 1.92960971 | -4.917765 | IS |
| -1.6399743 | 7.23226703 | IS |
| 5.20292254 | -0.5919079 | IS |
| 8.2120305 | 1.2069763 | IS |
| 5.15814267 | -1.1586608 | IS |
| 2.40583636 | 6.77637437 | IS |
| 4.37576059 | 6.39036527 | IS |
| 2.35625603 | 1.25955565 | IS |
| 2.36634663 | -4.3315965 | IS |
| -2.045044 | 6.40710429 | IS |
| 7.32862183 | -1.8941171 | IS |
| 4.9150635 | 7.52454531 | IS |
| 3.16762989 | 0.56780318 | IS |
| -0.3868703 | 0.36031303 | IS |
| 3.52904395 | -1.2420332 | IS |
| 5.19402978 | 1.27806715 | IS |
| 6.25326815 | -3.5653407 | IS |
| -1.9133296 | 4.49643181 | IS |
| 1.77780959 | -2.3872742 | IS |
| 1.10228008 | 1.02198138 | IS |
| -0.847038 | 1.53203536 | IS |
| 3.03211948 | -0.0989883 | IS |
| 5.5372329 | 3.22414176 | IS |
| -0.8237781 | 4.35874838 | IS |
| 6.74344085 | -3.7646076 | IS |
| 4.90437656 | -3.2400945 | IS |
| 3.31639984 | -1.7396497 | IS |
| 2.45302024 | 2.98853874 | IS |
| 4.11035718 | -0.332742 | IS |
| 4.23957088 | 3.57611183 | IS |
| -10.72022 | -4.318657 | IS |
| 8.27464645 | 1.27097188 | IS |
| 4.23247437 | 6.58873318 | IS |
| 4.85127634 | 5.06607696 | IS |
| 2.34951577 | 3.72579018 | IS |
| -0.6636767 | 5.77614281 | IS |
| 0.36540529 | 5.06778567 | IS |
| 6.71708456 | -1.0500682 | IS |
| 0.52002903 | 2.8601581 | IS |
