# Supplementary material for: Toxicological analysis of metabolites in ischemic stroke based on salivary metabolomics
Source: Front Mol Biosci. 2025 Aug 29;12:1609227. doi: 10.3389/fmolb.2025.1609227 (PMC12425714; doi:10.3389/fmolb.2025.1609227)
Supplement: Supplementary file 5 [file Supplementaryfile9.docx]

R scripts used in the analysis

# 1. t-SNE and UMAP analysis code

1. # 加载必要的库

2. if (!requireNamespace("Rtsne", quietly = TRUE)) install.packages("Rtsne")

3. if (!requireNamespace("umap", quietly = TRUE)) install.packages("umap")

4. if (!requireNamespace("ggplot2", quietly = TRUE)) install.packages("ggplot2")

5. if (!requireNamespace("dplyr", quietly = TRUE)) install.packages("dplyr")

6.

7. library(Rtsne)

8. library(umap)

9. library(ggplot2)

10. library(dplyr)

11.

12. # 设置工作目录

13. setwd("/Users/liuyansong/Documents/论文/论文练笔/Metabolomics_CyberToxicology/1.差异代谢物筛选")

14.

15. # 读取数据

16. data <- read.delim("差异代谢物表达量.txt", header = TRUE, sep = "\t", check.names = FALSE)

17.

18. # 查看数据结构

19. print(head(data))

20.

21. # 提取代谢物名称和样本数据

22. metabolite_names <- data[, 1]

23. data <- data[, -1] # 去掉第一列（代谢物名称）

24.

25. # 数据标准化（Z-score）

26. data_scaled <- scale(t(data)) # 转置数据，以便对样本进行标准化

27.

28. # 检查样本数量

29. n_samples <- nrow(data_scaled)

30. print(n_samples)

31.

32. # t-SNE分析

33. set.seed(123) # 设置随机种子以确保结果可重复

34. tsne_result <- Rtsne(data_scaled, dims = 2, perplexity = 10, theta = 0.5, check_duplicates = FALSE)

35.

36. # 绘制t-SNE结果

37. tsne_df <- as.data.frame(tsne_result$Y)

38. tsne_df$group <- c(rep("CON", 30), rep("IS", 40)) # 假设前30个为CON组，后40个为IS组

39.

40. tsne_plot <- ggplot(tsne_df, aes(x = V1, y = V2, color = group)) +

41. geom_point(size = 3, alpha = 0.7) +

42. labs(title = "t-SNE Visualization", x = "t-SNE Dimension 1", y = "t-SNE Dimension 2") +

43. theme_minimal()

44.

45. # 保存t-SNE可视化结果为图片

46. ggsave("t-SNE_visualization.png", plot = tsne_plot, width = 8, height = 6, dpi = 300)

47.

48. # UMAP分析

49. umap_result <- umap(data_scaled, n_neighbors = 15, min_dist = 0.1, n_components = 2)

50.

51. # 绘制UMAP结果

52. umap_df <- as.data.frame(umap_result$layout)

53. umap_df$group <- c(rep("CON", 30), rep("IS", 40)) # 假设前30个为CON组，后40个为IS组

54.

55. umap_plot <- ggplot(umap_df, aes(x = V1, y = V2, color = group)) +

56. geom_point(size = 3, alpha = 0.7) +

57. labs(title = "UMAP Visualization", x = "UMAP Dimension 1", y = "UMAP Dimension 2") +

58. theme_minimal()

59.

60. # 保存UMAP可视化结果为图片

61. ggsave("UMAP_visualization.png", plot = umap_plot, width = 8, height = 6, dpi = 300)

62.

63. # 保存结果到文件

64. write.csv(tsne_df, "t-SNE_results.csv", row.names = FALSE)

65. write.csv(umap_df, "UMAP_results.csv", row.names = FALSE)

# 2. Prediction sample size code

1. # 安装并加载pwr包

2. install.packages("pwr")

3. library(pwr)

4.

5. # 假设中等效应（d=0.8）、显著性水平α=0.05、效能power=0.8

6. result <- pwr.t.test(d = 0.8, sig.level = 0.05, power = 0.8,

7. type = "two.sample", alternative = "two.sided")

8.

9. # 查看每组所需样本量

10. print(result)

# 3. PCA analysis code

1. # 环境设置 ---------------------------------------------------------------

2. rm(list = ls())

3. setwd("/Users/liuyansong/Desktop/Metabolomics_CyberToxicology/")

4.

5. # 加载必要的包 ------------------------------------------------------------

6. if (!require("tidyverse")) install.packages("tidyverse")

7. if (!require("FactoMineR")) install.packages("FactoMineR")

8. if (!require("factoextra")) install.packages("factoextra")

9.

10. library(tidyverse)

11. library(FactoMineR)

12. library(factoextra)

13.

14. # 数据读取与预处理 ---------------------------------------------------------

15. pca_data <- read.delim("PCA.txt", check.names = FALSE, na.strings = c("NA", "NaN", ""))

16.

17. expression_matrix <- pca_data %>%

18. select(matches("^(CON_|IS_)")) %>%

19. t() %>%

20. as.data.frame() %>%

21. `colnames<-`(pca_data$`Metabolite name`)

22.

23. # 数据清洗（移除零方差及异常列） --------------------------------------------

24. col_vars <- apply(expression_matrix, 2, var, na.rm = TRUE)

25. nonzero_var_cols <- which(col_vars > 1e-10)

26. expression_matrix_clean <- expression_matrix[, nonzero_var_cols]

27.

28. # 标准化处理 ---------------------------------------------------------------

29. scaled_matrix <- scale(expression_matrix_clean)

30.

31. # 处理潜在的NA/Inf --------------------------------------------------------

32. scaled_matrix[is.na(scaled_matrix) | is.infinite(scaled_matrix)] <- 0

33.

34. # 维度验证 -----------------------------------------------------------------

35. if (ncol(scaled_matrix) == 0 || nrow(scaled_matrix) < 2) {

36. stop("数据维度不足，无法进行PCA分析")

37. }

38.

39. # PCA计算 ------------------------------------------------------------------

40. pca_result <- prcomp(scaled_matrix, center = FALSE, scale. = FALSE)

41.

42. # 可视化前3个主成分 --------------------------------------------------------

43. fviz_pca_ind(pca_result, axes = c(1,2), geom = "point", title = "PCA - Dim1 vs Dim2")

44. fviz_pca_ind(pca_result, axes = c(1,3), geom = "point", title = "PCA - Dim1 vs Dim3")

45.

46. # 代谢物贡献度分析 ----------------------------------------------------------

47. contributions <- pca_result$rotation[, 1:3] %>% # 取前3个主成分

48. as.data.frame() %>%

49. mutate(

50. PC1_abs = abs(PC1), # 载荷绝对值为贡献度指标

51. PC2_abs = abs(PC2),

52. PC3_abs = abs(PC3),

53. Metabolite = rownames(.)

54. ) %>%

55. arrange(desc(PC1_abs))

56.

57. # 保存分析结果 -------------------------------------------------------------

58. write_csv(contributions, "PCA_Metabolite_Contributions.csv")

59.

60. # 推荐保留的高载荷代谢物 ----------------------------------------------------

61. high_contrib_metabolites <- contributions %>%

62. filter(PC1_abs > quantile(PC1_abs, 0.90) | # 筛选前10%

63. PC2_abs > quantile(PC2_abs, 0.90) |

64. PC3_abs > quantile(PC3_abs, 0.90))

65.

66. write_csv(high_contrib_metabolites %>% select(Metabolite),

67. "High_Contribution_Metabolites.csv")

# 4. Compound target integration code

1. #install.packages("ggvenn")

2.

3.

4. #引用包

5. library(ggvenn)

6.

7. setwd("C:\\Users\\lexb\\Desktop\\Toxicity\\09.compound") #设置工作目录

8. files=list.files(pattern="*.txt$") #获取目录下txt结尾的文件

9. geneList=list()

10.

11. #读取所有txt文件中的基因信息，保存到geneList

12. for(inputFile in files){

13. if(inputFile=="compound.txt"){next}

14. rt=read.table(inputFile, header=F, sep="\t", check.names=F) #读取输入文件

15. geneNames=unlist(strsplit(as.vector(rt[,1]), " ")) #提取基因名称

16. geneNames=gsub("^ | $","",geneNames) #去掉基因首尾的空格

17. uniqGene=unique(geneNames) #基因取unique

18. header=unlist(strsplit(inputFile,"\\.|\\-"))

19. geneList[[header[1]]]=uniqGene

20. }

21. #绘制venn图

22. pdf(file="venn.pdf", width=6, height=6)

23. ggvenn(geneList,show_percentage = T,

24. stroke_color = "white", stroke_size = 0.5,

25. fill_color = c("#E41A1C","#1E90FF","#FF8C00"),

26. set_name_color =c("#E41A1C","#1E90FF","#FF8C00"),

27. set_name_size=6, text_size=4.5)

28. dev.off()

29.

30. #输出三个数据库的并集基因

31. unionGenes=Reduce(union, geneList)

32. write.table(file="Compound.txt", unionGenes, sep="\t", quote=F, col.names=F, row.names=F)

# 5. Disease target integration code

1. #install.packages("ggvenn")

2.

3.

4. #引用包

5. library(ggvenn)

6.

7. setwd("C:\\Users\\lexb\\Desktop\\Toxicity\\13.Disease") #设置工作目录

8. files=list.files(pattern="*.txt$") #获取目录下txt结尾的文件

9. geneList=list()

10.

11. #读取所有txt文件中的基因信息，保存到geneList

12. for(inputFile in files){

13. if(inputFile=="Disease.txt"){next}

14. rt=read.table(inputFile, header=F, sep="\t", check.names=F) #读取输入文件

15. geneNames=unlist(strsplit(as.vector(rt[,1]), " ")) #提取基因名称

16. geneNames=gsub("^ | $","",geneNames) #去掉基因首尾的空格

17. uniqGene=unique(geneNames) #基因取unique

18. header=unlist(strsplit(inputFile,"\\.|\\-"))

19. geneList[[header[1]]]=uniqGene

20. }

21. #绘制venn图

22. pdf(file="venn.pdf", width=6, height=6)

23. ggvenn(geneList,show_percentage = T,

24. stroke_color = "white", stroke_size = 0.5,

25. fill_color = c("#E41A1C","#1E90FF","#FF8C00"),

26. set_name_color =c("#E41A1C","#1E90FF","#FF8C00"),

27. set_name_size=6, text_size=4.5)

28. dev.off()

29.

30. #输出三个数据库的并集基因

31. unionGenes=Reduce(union, geneList)

32. write.table(file="Disease.txt", unionGenes, sep="\t", quote=F, col.names=F, row.names=F)

# 6. Compound and disease target intersection code

1. #install.packages("ggvenn")

2.

3.

4. #引用包

5. library(ggvenn)

6.

7. compoundName="DEP" #化合物名称

8. diseaseName="Brain injury" #疾病名称

9. setwd("C:\\Users\\lexb\\Desktop\\Toxicity\\14.venn") #设置工作目录

10. geneList=list()

11.

12. #读取化合物的基因列表文件

13. rt=read.table("Compound.txt", header=F, sep="\t", check.names=F)

14. geneNames=as.vector(rt[,1]) #提取基因名称

15. geneList[[compoundName]]=geneNames

16.

17. #读取疾病的基因列表文件

18. rt=read.table("Disease.txt", header=F, sep="\t", check.names=F)

19. geneNames=as.vector(rt[,1]) #提取基因名称

20. geneList[[diseaseName]]=geneNames

21.

22. #绘制venn图

23. pdf(file="venn.pdf", width=6, height=6)

24. ggvenn(geneList,show_percentage = T,

25. stroke_color = "white", stroke_size = 0.5,

26. fill_color = c("#E41A1C","#1E90FF"),

27. set_name_color =c("#E41A1C","#1E90FF"),

28. set_name_size=6, text_size=4.5)

29. dev.off()

30.

31. #输出交集基因

32. interGenes=Reduce(intersect, geneList)

33. write.table(file="interGenes.txt", interGenes, sep="\t", quote=F, col.names=F, row.names=F)

34.

35. #输出网络关系文件

36. networkTab=rbind(cbind(compoundName,interGenes, "Compound"), cbind(diseaseName,interGenes, "Disease"))

37. colnames(networkTab)=c("Node1", "Node2", "Type")

38. write.table(file="net.network.txt", networkTab, sep="\t", quote=F, row.names=F)

39. #输出节点属性文件

40. nodeTab=rbind(cbind(compoundName,"Compound"), cbind(diseaseName,"Disease"), cbind(interGenes,"Gene"))

41. colnames(nodeTab)=c("Node", "Type")

42. write.table(file="net.node.txt", nodeTab, sep="\t", quote=F, row.names=F)

# 7. GO analysis code

1. #install.packages("colorspace")

2. #install.packages("stringi")

3. #install.packages("ggplot2")

4. #install.packages("circlize")

5. #install.packages("RColorBrewer")

6. #install.packages("ggpubr")

7.

8. #if (!requireNamespace("BiocManager", quietly = TRUE))

9. # install.packages("BiocManager")

10. #BiocManager::install("org.Hs.eg.db")

11. #BiocManager::install("DOSE")

12. #BiocManager::install("clusterProfiler")

13. #BiocManager::install("enrichplot")

14. #BiocManager::install("ComplexHeatmap")

15.

16.

17. #引用包

18. library(clusterProfiler)

19. library(org.Hs.eg.db)

20. library(enrichplot)

21. library(ggplot2)

22. library(circlize)

23. library(RColorBrewer)

24. library(dplyr)

25. library("ggpubr")

26. library(ComplexHeatmap)

27.

28. pvalueFilter=0.05 #p值过滤条件

29. p.adjustFilter=0.05 #矫正后的p值过滤条件

30.

31. #定义图形的颜色

32. colorSel="p.adjust"

33. if(p.adjustFilter>0.05){

34. colorSel="pvalue"

35. }

36.

37. setwd("C:\\Users\\lexb\\Desktop\\Toxicity\\16.GO") #设置工作目录

38. rt=read.table("interGenes.txt", header=F, sep="\t", check.names=F) #读取输入文件

39.

40. #提取交集基因的名称, 将基因名称转换为基因id

41. genes=unique(as.vector(rt[,1]))

42. entrezIDs=mget(genes, org.Hs.egSYMBOL2EG, ifnotfound=NA)

43. entrezIDs=as.character(entrezIDs)

44. gene=entrezIDs[entrezIDs!="NA"] #去除基因id为NA的基因

45. #gene=gsub("c\\(\"(\\d+)\".*", "\\1", gene)

46.

47. #GO富集分析

48. kk=enrichGO(gene=gene, OrgDb=org.Hs.eg.db, pvalueCutoff=1, qvalueCutoff=1, ont="all", readable=T)

49. GO=as.data.frame(kk)

50. GO=GO[(GO$pvalue<pvalueFilter & GO$p.adjust<p.adjustFilter),]

51. #输出显著富集的结果

52. write.table(GO, file="GO.txt", sep="\t", quote=F, row.names = F)

53.

54. #柱状图

55. pdf(file="barplot.pdf", width=8, height=7)

56. bar=barplot(kk, drop=TRUE, showCategory=10, label_format=100, split="ONTOLOGY", color=colorSel) + facet_grid(ONTOLOGY~., scale='free')

57. print(bar)

58. dev.off()

59.

60. #气泡图

61. pdf(file="bubble.pdf", width=8, height=7)

62. bub=dotplot(kk, showCategory=10, orderBy="GeneRatio", label_format=100, split="ONTOLOGY", color=colorSel) + facet_grid(ONTOLOGY~., scale='free')

63. print(bub)

64. dev.off()

65.

66. #绘制分类柱状图

67. data=GO %>% group_by(ONTOLOGY) %>% slice_head(n=10)

68. pdf(file="barplot.color.pdf", width=11, height=8)

69. ggbarplot(data, x="Description", y="Count", fill = "ONTOLOGY", color = "white", xlab="",

70. palette = "aaas", #设置图形的颜色方案

71. legend = "right", #图例位置

72. sort.val = "desc", #GO排序方式

73. sort.by.groups=TRUE)+ #根据GO的分类进行排序

74. rotate_x_text(75)+ #x轴字体倾斜的角度

75. theme(panel.background = element_blank(), axis.text.x=element_text(size=10, color="black"))+

76. scale_y_continuous(expand=c(0, 0)) + scale_x_discrete(expand=c(0,0))

77. dev.off()

78.

79.

80. ###########绘制GO圈图###########

81. ontology.col=c("#00CC33FF", "#FFC20AFF", "#CC33FFFF")

82. data=GO[order(GO$p.adjust),]

83. datasig=data[data$pvalue<0.05,,drop=F]

84. BP = datasig[datasig$ONTOLOGY=="BP",,drop=F]

85. CC = datasig[datasig$ONTOLOGY=="CC",,drop=F]

86. MF = datasig[datasig$ONTOLOGY=="MF",,drop=F]

87. BP = head(BP,6)

88. CC = head(CC,6)

89. MF = head(MF,6)

90. data = rbind(BP,CC,MF)

91. main.col = ontology.col[as.numeric(as.factor(data$ONTOLOGY))]

92.

93. #整理圈图数据

94. BgGene = as.numeric(sapply(strsplit(data$BgRatio,"/"),'[',1))

95. Gene = as.numeric(sapply(strsplit(data$GeneRatio,'/'),'[',1))

96. ratio = Gene/BgGene

97. logpvalue = -log(data$pvalue,10)

98. logpvalue.col = brewer.pal(n = 8, name = "Reds")

99. f = colorRamp2(breaks = c(0,2,4,6,8,10,15,20), colors = logpvalue.col)

100. BgGene.col = f(logpvalue)

101. df = data.frame(GO=data$ID,start=1,end=max(BgGene))

102. rownames(df) = df$GO

103. bed2 = data.frame(GO=data$ID,start=1,end=BgGene,BgGene=BgGene,BgGene.col=BgGene.col)

104. bed3 = data.frame(GO=data$ID,start=1,end=Gene,BgGene=Gene)

105. bed4 = data.frame(GO=data$ID,start=1,end=max(BgGene),ratio=ratio,col=main.col)

106. bed4$ratio = bed4$ratio/max(bed4$ratio)*9.5

107.

108. #绘制圈图的主体部分

109. pdf(file="GO.circlize.pdf", width=10, height=10)

110. par(omi=c(0.1,0.1,0.1,1.5))

111. circos.par(track.margin=c(0.01,0.01))

112. circos.genomicInitialize(df,plotType="none")

113. circos.trackPlotRegion(ylim = c(0, 1), panel.fun = function(x, y) {

114. sector.index = get.cell.meta.data("sector.index")

115. xlim = get.cell.meta.data("xlim")

116. ylim = get.cell.meta.data("ylim")

117. circos.text(mean(xlim), mean(ylim), sector.index, cex = 0.8, facing = "bending.inside", niceFacing = TRUE)

118. }, track.height = 0.08, bg.border = NA,bg.col = main.col)

119.

120. for(si in get.all.sector.index()) {

121. circos.axis(h = "top", labels.cex = 0.6, sector.index = si,track.index = 1,

122. major.at=seq(0,max(BgGene),by=100),labels.facing = "clockwise")

123. }

124. f = colorRamp2(breaks = c(-1, 0, 1), colors = c("green", "black", "red"))

125. circos.genomicTrack(bed2, ylim = c(0, 1),track.height = 0.1,bg.border="white",

126. panel.fun = function(region, value, ...) {

127. i = getI(...)

128. circos.genomicRect(region, value, ytop = 0, ybottom = 1, col = value[,2],

129. border = NA, ...)

130. circos.genomicText(region, value, y = 0.4, labels = value[,1], adj=0,cex=0.8,...)

131. })

132. circos.genomicTrack(bed3, ylim = c(0, 1),track.height = 0.1,bg.border="white",

133. panel.fun = function(region, value, ...) {

134. i = getI(...)

135. circos.genomicRect(region, value, ytop = 0, ybottom = 1, col = '#BA55D3',

136. border = NA, ...)

137. circos.genomicText(region, value, y = 0.4, labels = value[,1], cex=0.9,adj=0,...)

138. })

139. circos.genomicTrack(bed4, ylim = c(0, 10),track.height = 0.35,bg.border="white",bg.col="grey90",

140. panel.fun = function(region, value, ...) {

141. cell.xlim = get.cell.meta.data("cell.xlim")

142. cell.ylim = get.cell.meta.data("cell.ylim")

143. for(j in 1:9) {

144. y = cell.ylim[1] + (cell.ylim[2]-cell.ylim[1])/10*j

145. circos.lines(cell.xlim, c(y, y), col = "#FFFFFF", lwd = 0.3)

146. }

147. circos.genomicRect(region, value, ytop = 0, ybottom = value[,1], col = value[,2],

148. border = NA, ...)

149. #circos.genomicText(region, value, y = 0.3, labels = value[,1], ...)

150. })

151. circos.clear()

152. #绘制圈图中间的图例

153. middle.legend = Legend(

154. labels = c('Number of Genes','Number of Select','Rich Factor(0-1)'),

155. type="points",pch=c(15,15,17),legend_gp = gpar(col=c('pink','#BA55D3',ontology.col[1])),

156. title="",nrow=3,size= unit(3, "mm")

157. )

158. circle_size = unit(1, "snpc")

159. draw(middle.legend,x=circle_size*0.42)

160. #绘制GO分类的图例

161. main.legend = Legend(

162. labels = c("Biological Process","Cellular Component", "Molecular Function"), type="points",pch=15,

163. legend_gp = gpar(col=ontology.col), title_position = "topcenter",

164. title = "ONTOLOGY", nrow = 3,size = unit(3, "mm"),grid_height = unit(5, "mm"),

165. grid_width = unit(5, "mm")

166. )

167. #绘制富集显著性pvalue的图例

168. logp.legend = Legend(

169. labels=c('(0,2]','(2,4]','(4,6]','(6,8]','(8,10]','(10,15]','(15,20]','>=20'),

170. type="points",pch=16,legend_gp=gpar(col=logpvalue.col),title="-log10(Pvalue)",

171. title_position = "topcenter",grid_height = unit(5, "mm"),grid_width = unit(5, "mm"),

172. size = unit(3, "mm")

173. )

174. lgd = packLegend(main.legend,logp.legend)

175. circle_size = unit(1, "snpc")

176. print(circle_size)

177. draw(lgd, x = circle_size*0.85, y=circle_size*0.55,just = "left")

178. dev.off()

# 8. KEGG analysis code

1. #install.packages("colorspace")

2. #install.packages("stringi")

3. #install.packages("ggplot2")

4. #install.packages("circlize")

5. #install.packages("RColorBrewer")

6. #install.packages("ggpubr")

7.

8. #if (!requireNamespace("BiocManager", quietly = TRUE))

9. # install.packages("BiocManager")

10. #BiocManager::install("org.Hs.eg.db")

11. #BiocManager::install("DOSE")

12. #BiocManager::install("clusterProfiler")

13. #BiocManager::install("enrichplot")

14. #BiocManager::install("ComplexHeatmap")

15.

16.

17. #引用包

18. library(clusterProfiler)

19. library(org.Hs.eg.db)

20. library(enrichplot)

21. library(ggplot2)

22. library(circlize)

23. library(RColorBrewer)

24. library(dplyr)

25. library(ggpubr)

26.

27. pvalueFilter=0.05 #p值过滤条件

28. p.adjustFilter=0.05 #矫正后的p值过滤条件

29.

30. #定义图形的颜色

31. colorSel="p.adjust"

32. if(p.adjustFilter>0.05){

33. colorSel="pvalue"

34. }

35.

36. setwd("C:\\Users\\lexb\\Desktop\\Toxicity\\17.KEGG") #设置工作目录

37. rt=read.table("interGenes.txt", header=F, sep="\t", check.names=F) #读取输入文件

38.

39. #提取交集基因的名称,将基因名字转换为基因id

40. genes=unique(as.vector(rt[,1]))

41. entrezIDs=mget(genes, org.Hs.egSYMBOL2EG, ifnotfound=NA)

42. entrezIDs=as.character(entrezIDs)

43. rt=data.frame(genes, entrezID=entrezIDs)

44. gene=entrezIDs[entrezIDs!="NA"] #去除基因id为NA的基因

45. #gene=gsub("c\\(\"(\\d+)\".*", "\\1", gene)

46.

47. #KEGG富集分析

48. kk <- enrichKEGG(gene=gene, organism="hsa", pvalueCutoff=1, qvalueCutoff=1)

49. KEGG=as.data.frame(kk)

50. KEGG$geneID=as.character(sapply(KEGG$geneID,function(x)paste(rt$genes[match(strsplit(x,"/")[[1]],as.character(rt$entrezID))],collapse="/")))

51. KEGG=KEGG[(KEGG$pvalue<pvalueFilter & KEGG$p.adjust<p.adjustFilter),]

52. #保存显著富集的结果

53. write.table(KEGG, file="KEGG.txt", sep="\t", quote=F, row.names = F)

54.

55. #定义展示通路的数目

56. showNum=30 #显示富集最显著的前30个通路

57. if(nrow(KEGG)<showNum){

58. showNum=nrow(KEGG)

59. }

60.

61. #柱状图

62. pdf(file="barplot.pdf", width=8, height=7)

63. barplot(kk, drop=TRUE, showCategory=showNum, label_format=130, color=colorSel)

64. dev.off()

65.

66. #气泡图

67. pdf(file="bubble.pdf", width=8, height=7)

68. dotplot(kk, showCategory=showNum, orderBy="GeneRatio", label_format=130, color=colorSel)

69. dev.off()

70.

71. #绘制棒棒糖图

72. pdf(file="Lollipop.pdf", width=9.5, height=6)

73. ggdotchart(KEGG, x="Description", y="Count", color = "category",group = "category", xlab="",

74. palette = "aaas", #配色方案

75. legend = "right", #图例位置

76. sorting = "descending", #图形排序方式(上升排序,区别于desc)

77. add = "segments", #增加线段

78. rotate = TRUE, #图形以横向的形式展示

79. dot.size = 7, #圆圈大小

80. label = round(KEGG$Count), #圆圈内数值

81. font.label = list(color="white",size=12, vjust=0.5), #圆圈内数值的字体

82. ggtheme = theme_pubr())

83. dev.off()

# 9. NIHSS and metabolite correlation analysis code

1. # 加载必要R包

2. if (!require("tidyverse")) install.packages("tidyverse")

3. if (!require("pheatmap")) install.packages("pheatmap")

4. if (!require("ggplot2")) install.packages("ggplot2")

5.

6. library(tidyverse)

7. library(pheatmap)

8. library(ggplot2)

9.

10. # 设置工作目录

11. setwd("/Users/liuyansong/Documents/论文/论文练笔/Metabolomics_CyberToxicology/1.差异代谢物筛选")

12.

13. # 读取代谢物数据

14. met_data <- read.delim("488.txt", check.names = FALSE)

15. rownames(met_data) <- met_data$Metabolite_name

16. met_data <- met_data[ , grep("^IS_", colnames(met_data))] # 提取 IS 样本

17.

18. # 转置：行=样本，列=代谢物

19. met_data_t <- t(met_data)

20. met_df <- as.data.frame(met_data_t)

21. met_df$Sample <- rownames(met_df)

22.

23. # 读取NIHSS评分表

24. nihss <- read.delim("NIHSS.txt") # 或读取新文件名

25. colnames(nihss) <- c("Sample", "NIHSS")

26.

27. # 检查样本匹配

28. cat("IS样本数（代谢物）：", nrow(met_df), "\n")

29. cat("IS样本数（NIHSS）：", nrow(nihss), "\n")

30. intersected <- intersect(nihss$Sample, met_df$Sample)

31. cat("匹配样本数：", length(intersected), "\n")

32.

33. # 合并数据

34. merged <- inner_join(nihss, met_df, by = "Sample")

35.

36. # Spearman相关分析

37. cor_results <- lapply(colnames(merged)[3:ncol(merged)], function(met) {

38. x <- merged[[met]]

39. y <- merged$NIHSS

40. cor_test <- cor.test(x, y, method = "spearman")

41. data.frame(

42. Metabolite = met,

43. SpearmanR = cor_test$estimate,

44. P.value = cor_test$p.value

45. )

46. })

47. cor_df <- do.call(rbind, cor_results)

48.

49. # FDR校正

50. cor_df$FDR <- p.adjust(cor_df$P.value, method = "fdr")

51.

52. # 保存所有结果

53. write.csv(cor_df, "Spearman_correlation_NIHSS_all.csv", row.names = FALSE)

54.

55. # 显著结果（FDR < 0.05）

56. sig_df <- cor_df %>% filter(FDR < 0.05)

57. write.csv(sig_df, "Spearman_correlation_NIHSS_significant.csv", row.names = FALSE)

58.

59. # 前20名（无论显著与否）

60. top_df <- cor_df %>% arrange(FDR) %>% head(20)

61. write.csv(top_df, "Spearman_correlation_NIHSS_top20.csv", row.names = FALSE)

62.

63. # 绘制散点图：前5名趋势图

64. top_to_plot <- head(top_df$Metabolite, 5)

65.

66. for (met in top_to_plot) {

67. p <- ggplot(merged, aes(x = .data[[met]], y = NIHSS)) +

68. geom_point(color = "steelblue") +

69. geom_smooth(method = "lm", se = FALSE, color = "firebrick") +

70. labs(

71. title = paste("Trend: ", met),

72. x = paste0(met, " (peak area)"),

73. y = "NIHSS"

74. ) +

75. theme_minimal(base_size = 14)

76.

77. ggsave(

78. paste0("NIHSS_", gsub("[^A-Za-z0-9]", "_", met), "_scatter_trend.png"),

79. plot = p, dpi = 600, width = 6, height = 5

80. )

81. }

82.

83. # 可选：绘制热图（若有显著结果）

84. if (nrow(sig_df) > 1) {

85. mat <- merged %>%

86. select(Sample, all_of(sig_df$Metabolite)) %>%

87. column_to_rownames("Sample") %>%

88. as.matrix()

89. png("NIHSS_Metabolite_Heatmap.png", width = 2500, height = 1600, res = 300)

90. pheatmap::pheatmap(mat, cluster_rows = TRUE, cluster_cols = TRUE, scale = "row",

91. main = "Significant Metabolites vs NIHSS")

92. dev.off()

93. }

# 10. Comparison code of CV of internal standard in each group

1. # 加载必要包

2. library(dplyr)

3. library(tidyr)

4. library(ggplot2)

5.

6. # 设定文件路径

7. file_path <- "/Users/liuyansong/Desktop/Internal_Standard.txt"

8. save_dir <- "/Users/liuyansong/Documents/论文/论文练笔/Metabolomics_CyberToxicology/1.差异代谢物筛选"

9.

10. # 读取数据

11. df <- read.table(file_path, header = TRUE, sep = "\t", check.names = FALSE)

12.

13. # 查看列名，确认样本列格式

14. head(colnames(df))

15.

16. # 转为长格式

17. df_long <- df %>%

18. pivot_longer(cols = -1, names_to = "Sample", values_to = "Intensity") %>%

19. rename(Metabolite = 1)

20.

21. # 加入分组信息（IS vs Control）

22. df_long <- df_long %>%

23. mutate(Group = case_when(

24. grepl("^IS", Sample, ignore.case = TRUE) ~ "IS",

25. grepl("^CON", Sample, ignore.case = TRUE) ~ "Control",

26. TRUE ~ "Other"

27. )) %>%

28. filter(Group != "Other")

29.

30. # 计算每个内标在不同组的CV

31. cv_df <- df_long %>%

32. group_by(Metabolite, Group) %>%

33. summarise(CV = sd(Intensity, na.rm = TRUE) / mean(Intensity, na.rm = TRUE) * 100,

34. .groups = "drop")

35.

36. # 绘图

37. p <- ggplot(cv_df, aes(x = Metabolite, y = CV, fill = Group)) +

38. geom_bar(stat = "identity", position = "dodge", width = 0.7) +

39. labs(title = "Coefficient of Variation of Internal Standards",

40. y = "CV (%)", x = "Internal Standard") +

41. theme_minimal(base_size = 14) +

42. theme(axis.text.x = element_text(angle = 45, hjust = 1),

43. legend.position = "top") +

44. scale_fill_manual(values = c("IS" = "#D95F02", "Control" = "#1B9E77"))

45.

46. # 保存图像

47. ggsave(filename = file.path(save_dir, "Internal_Standards_CV_Comparison.png"),

48. plot = p, dpi = 600, width = 8, height = 6)

49.

50. # 保存CV数据为CSV

51. write.csv(cv_df,

52. file = file.path(save_dir, "Internal_Standards_CV_Values.csv"),

53. row.names = FALSE)

54.

55. # 筛选出 QC 样本

56. qc_data <- df %>%

57. select(starts_with("PQC") | starts_with("QC"))

58.

59. # 确保有QC样本存在

60. if (ncol(qc_data) > 0) {

61.

62. # 计算每个QC样本的总峰面积

63. qc_signal <- colSums(qc_data, na.rm = TRUE)

64. qc_df <- data.frame(Sample = names(qc_signal), TotalSignal = qc_signal)

65.

66. # 添加顺序（采样顺序）

67. qc_df$Order <- seq_along(qc_df$Sample)

68.

69. # 绘图

70. p_qc <- ggplot(qc_df, aes(x = Order, y = TotalSignal)) +

71. geom_line(color = "steelblue", size = 1.2) +

72. geom_point(color = "firebrick", size = 2) +

73. labs(title = "QC Sample Total Signal Trend",

74. x = "Injection Order", y = "Total Signal Intensity") +

75. theme_minimal(base_size = 14)

76.

77. # 保存图像

78. ggsave(filename = file.path(save_dir, "QC_Total_Signal_Trend.png"),

79. plot = p_qc, dpi = 600, width = 8, height = 5)

80.

81. } else {

82. message("❗未检测到以 'PQC' 或 'QC' 开头的样本列，请确认QC样本名称。")

83. }

84.
