# Supplementary material for: Toxicological analysis of metabolites in ischemic stroke based on salivary metabolomics
Source: Front Mol Biosci. 2025 Aug 29;12:1609227. doi: 10.3389/fmolb.2025.1609227 (PMC12425714; doi:10.3389/fmolb.2025.1609227)
Supplement: Supplementary file 6 [file Supplementaryfile7.docx]

Top 20 metabolite-NIHSS correlation

| Metabolite | SpearmanR | P.value | FDR |
| --- | --- | --- | --- |
| 1,4-Cyclohexanedicarboxylic_Acid | 0.0083087 | 0.95941847 | 0.99815497 |
| 1,5-Naphthalenediamine | 0.03086859 | 0.85002674 | 0.99815497 |
| 1-Aminocyclopropane-1-Carboxylate | -0.063814 | 0.69565225 | 0.99815497 |
| 1-Methylcytosine | 0.03860934 | 0.81301999 | 0.99815497 |
| 1-Methylguanine | 0.01831348 | 0.91069568 | 0.99815497 |
| 1-Methylhistidine | 0.14207105 | 0.38185069 | 0.99815497 |
| 1-Methylnicotinamide | -0.0625868 | 0.7012318 | 0.99815497 |
| 10-HYDROXYDECANOATE | -0.1602237 | 0.32334367 | 0.99815497 |
| 10-Hydroxydecanoic_acid | 0.04974847 | 0.76048146 | 0.99815497 |
| 11-HETE | -0.175478 | 0.27878238 | 0.99815497 |
| 12-HEPE | -0.0788249 | 0.62875692 | 0.99815497 |
| 12-HETE | -0.1762436 | 0.27665702 | 0.99815497 |
| 13-HODE_+_9-HODE | -0.1341415 | 0.40924541 | 0.99815497 |
| 13-HOTrE | -0.3097243 | 0.0517899 | 0.99815497 |
| 15-HEPE | -0.2824429 | 0.07742396 | 0.99815497 |
| 2'-Deoxyuridine | 0.01205648 | 0.94114048 | 0.99815497 |
| 2,3-Dihydroxybenzoic_acid | -0.1061049 | 0.51463979 | 0.99815497 |
| 2,4-DIHYDROXYBUTANOIC_ACID | 0.09062339 | 0.5781258 | 0.99815497 |
| 2-Aminocaprylic_acid | -0.1930467 | 0.23267947 | 0.99815497 |
| 2-Aminopyridine | 0.0006608 | 0.9967712 | 0.99815497 |
