# Supplementary material for: Toxicological analysis of metabolites in ischemic stroke based on salivary metabolomics
Source: Front Mol Biosci. 2025 Aug 29;12:1609227. doi: 10.3389/fmolb.2025.1609227 (PMC12425714; doi:10.3389/fmolb.2025.1609227)
Supplement: Supplementary file 8 [file Supplementaryfile2.docx]

List of differential metabolites

| **Metabolite name** | **Category1** | **Category2** | **HMDB ID** | **Average Rt(min)** | **Average Mz** | **Adduct type** | **Mode** | **S/N average** |
| --- | --- | --- | --- | --- | --- | --- | --- | --- |
| 1,4-Cyclohexanedicarboxylic Acid | Xenobiotics | Chemical | HMDB0250649 | 1.07 | 171.06557 | [M-H]- | Negative_NH4HCO3 | 36.75 |
| 1,5-Naphthalenediamine | Xenobiotics | Chemical | HMDB0244231 | 3.443 | 159.09146 | [M+ACN+H]+ | Positive | 28.21 |
| 1-Aminocyclopropane-1-Carboxylate | Xenobiotics | Natural Product/Food/Plant | HMDB0036458 | 1.233 | 102.06346 | [M+H]+ | Positive | 26.7 |
| 1-Methylcytosine | Nucleotide | Pyrimidine Metabolism, Cytidine containing | HMDB0243939 | 1.424 | 126.05508 | [M+H]+ | Positive | 12.86 |
| 1-Methylguanine | Nucleotide | Purine Metabolism, Guanine containing | HMDB0003282 | 1.321 | 166.07245 | [M+H]+ | Positive | 75.92 |
| 1-Methylhistidine | Amino Acid | Histidine Metabolism | HMDB0000001 | 1.068 | 170.09221 | [M+H]+ | Positive | 19.49 |
| 1-Methylnicotinamide | Cofactors and Vitamins | Nicotinate and Nicotinamide Metabolism | HMDB0000699 | 1.472 | 137.0791 | [M]+ | Positive | 61.37 |
| 10-HYDROXYDECANOATE | Lipid | Fatty Acid, Monohydroxy | HMDB0244272 | 5.595 | 211.12975 | [M+H]+ | Positive | 30.46 |
| 10-Hydroxydecanoic acid | Lipid | Fatty Acid, Monohydroxy | HMDB0094656 | 6.274 | 187.13345 | [M-H]- | Negative_FA | 19801.2 |
| 11-HETE | Unclassified | Unclassified |  | 7.896 | 319.22815 | [M-H]- | Negative_NH4HCO3 | 156440.5 |
| 12-HEPE | Lipid | Eicosanoid | HMDB0010202 | 7.818 | 317.21231 | [M-H]- | Negative_NH4HCO3 | 21343.71 |
| 12-HETE | Lipid | Eicosanoid | HMDB0006111 | 6.913 | 303.23157 | [M+H-H2O]+ | Positive | 95688.69 |
| 13-HODE + 9-HODE | Lipid | Fatty Acid, Monohydroxy | HMDB0004667 | 7.81 | 295.22766 | [M-H]- | Negative_NH4HCO3 | 207.68 |
| 13-HOTrE | Unclassified | Unclassified |  | 6.875 | 293.21252 | [M-H]1- | Negative_FA | 102034.4 |
| 15-HEPE | Lipid | Eicosanoid | HMDB0010209 | 6.877 | 317.20856 | [M-H]- | Positive | 407035.6 |
| 2'-Deoxyuridine | Nucleotide | Pyrimidine Metabolism, Uracil containing | HMDB0000012 | 1.459 | 227.0668 | [M-H]- | Negative_NH4HCO3 | 48.9 |
| 2,3-Dihydroxybenzoic acid | Xenobiotics | Benzoate Metabolism | HMDB0000397 | 4.562 | 153.02991 | [M-H]- | Negative_FA | 105.56 |
| 2,4-DIHYDROXYBUTANOIC ACID | Xenobiotics | Natural Product/Food/Plant | HMDB0000360 | 1.238 | 119.03107 | [M-H]- | Negative_FA | 92.99 |
| 2-Aminocaprylic acid | Xenobiotics | Natural Product/Food/Plant | HMDB0000991 | 4.921 | 160.13324 | [M+H]+ | Positive | 2663.82 |
| 2-Aminopyridine | Xenobiotics | Chemical | HMDB0245026 | 1.173 | 95.06091 | [M+H]+ | Positive | 28.11 |
| 2-Chloro-4-phenylphenol | Unclassified | Unclassified |  | 4 | 203.03207 | [M-H]- | Negative_FA | 28.09 |
| 2-Ethylhexylparaben | Xenobiotics | Natural Product/Food/Plant |  | 6.98 | 249.14896 | [M-H]- | Negative_NH4HCO3 | 37.84 |
| 2-Hydroxy-2-methylbutyric acid | Lipid | Fatty Acid, Monohydroxy | HMDB0001987 | 1.286 | 117.05441 | [M-H]- | Negative_NH4HCO3 | 158.04 |
| 2-Hydroxyacetophenone sulfate | Xenobiotics | Chemical |  | 3.4 | 215.00948 | [M-H]- | Negative_NH4HCO3 | 2.25 |
| 2-Hydroxybenzonitrile | Xenobiotics | Chemical |  | 2.145 | 120.04452 | [M+H]+ | Positive | 14.21 |
| 2-Hydroxybutyric Acid | Carbohydrate | Propanoate Metabolism | HMDB0000008 | 1.683 | 103.03902 | [M-H]- | Negative_FA | 49.42 |
| 2-Hydroxycaproic Acid | Lipid | Fatty Acid, Monohydroxy | HMDB0001624 | 2.15 | 131.07027 | [M-H]- | Negative_NH4HCO3 | 405.41 |
| 2-Hydroxyglutaric Acid | Carbohydrate | Citrate cycle (TCA cycle) | HMDB0059655 | 0.882 | 147.0289 | [M-H]- | Negative_NH4HCO3 | 1157.94 |
| 2-Hydroxyhippuric Acid (Salicylurate) | Xenobiotics | Benzoate Metabolism | HMDB0000840 | 4.951 | 194.04575 | [M-H]- | Negative_FA | 23.68 |
| 2-Hydroxyisovaleric acid | Amino Acid | Leucine, Isoleucine and Valine Metabolism | HMDB0000407 | 3.402 | 117.0548 | [M-H]- | Negative_FA | 792.94 |
| 2-Hydroxymyristate | Lipid | Fatty Acid, Monohydroxy | HMDB0002261 | 7.675 | 243.19629 | [M-H]- | Negative_NH4HCO3 | 208.55 |
| 2-Hydroxyoleate | Lipid | Fatty Acid, Monohydroxy |  | 7.985 | 297.24347 | [M-H]- | Negative_NH4HCO3 | 243.43 |
| 2-Hydroxystearate | Lipid | Fatty Acid, Monohydroxy | HMDB0062549 | 7.116 | 299.25885 | [M-H]- | Negative_FA | 1701193 |
| 2-Hydroxyvaleric acid | Amino Acid | Leucine, Isoleucine and Valine Metabolism | HMDB0001863 | 1.427 | 117.05475 | [M-H]- | Positive | 27.99 |
| 2-Isopropylmalic acid | Amino Acid | Leucine, Isoleucine and Valine Metabolism | HMDB0000402 | 4.096 | 175.06029 | [M-H]- | Negative_FA | 205.2 |
| 2-Methylbutyrylcarnitine (C5) | Amino Acid | Leucine, Isoleucine and Valine Metabolism | HMDB0000378 | 4.187 | 246.16965 | [M+H]+ | Positive | 268853.7 |
| 2-Methylcitrate/Homocitrate | Carbohydrate | Citrate cycle (TCA cycle) | HMDB0003518 | 0.839 | 205.03465 | [M-H]- | Negative_NH4HCO3 | 62.84 |
| 2-Methylcytidine | Nucleotide | Pyrimidine Metabolism, Cytidine containing | HMDB0242132 | 1.231 | 258.11935 | [M+H]+ | Positive | 41157.02 |
| 2-Naphthylamine | Xenobiotics | Natural Product/Food/Plant | HMDB0243964 | 3.392 | 144.08058 | [M+H]+ | Positive | 29.07 |
| 2-Phosphoglyceric Acid | Carbohydrate | Glycolysis, Gluconeogenesis, and Pyruvate Metabolism | HMDB0000362 | 0.856 | 184.98468 | [M-H]- | Negative_NH4HCO3 | 18.76 |
| 2-Piperidinone | Xenobiotics | Natural Product/Food/Plant | HMDB0011749 | 2.2 | 100.07606 | [M+H]+ | Positive | 6034.76 |
| 2-Pyrrolidinone | Xenobiotics | Chemical | HMDB0002039 | 1.595 | 86.06062 | [M+H]+ | Positive | 54.21 |
| 25-HYDROXYCHOLESTEROL | Lipid | Sterol | HMDB0006247 | 1.352 | 202.17999 | [M+H]+ | Positive | 60075.74 |
| 3-(3-Hydroxyphenyl)propionic acid sulfate | Unclassified | Unclassified |  | 3.657 | 245.01274 | [M-H]- | Negative_FA | 16.43 |
| 3-AMINO-4-HYDROXYBENZOIC ACID | Secondary metabolites | Biosynthesis of secondary metabolites | HMDB0304941 | 2.107 | 152.03447 | [M-H]- | Negative_FA | 24.43 |
| 3-Carboxy-4-Methyl-5-Propyl-2-Furanpropanoate (CMPF) | Lipid | Fatty Acid, Dicarboxylate | HMDB0061112 | 6.272 | 239.08974 | [M-H]- | Negative_FA | 36.46 |
| 3-Formylindole | Xenobiotics | Natural Product/Food/Plant | HMDB0029737 | 3.43 | 146.05986 | [M+H]+ | Positive | 42.43 |
| 3-Hydroxy-3-Methylglutaric Acid | Amino Acid | Leucine, Isoleucine and Valine Metabolism | HMDB0029169 | 1.481 | 161.04468 | [M-H]- | Negative_FA | 63.94 |
| 3-Hydroxy-l-kynurenine | Amino Acid | Tryptophan Metabolism | HMDB0011631 | 1.26 | 225.08432 | [M+H]+ | Positive | 15.21 |
| 3-Hydroxybutyric Acid | Lipid | Ketone Bodies | HMDB0000011 | 1.381 | 103.03918 | [M-H]- | Negative_FA | 42.92 |
| 3-Hydroxybutyrylcarnitine | Lipid | Fatty Acid Metabolism (Acyl Carnitine) | HMDB0013127 | 1.468 | 248.14969 | [M+H]+ | Positive | 40.19 |
| 3-Hydroxycotinine | Xenobiotics | Tobaco Metabolite | HMDB0001390 | 1.298 | 193.09712 | [M+H]+ | Positive | 58.05 |
| 3-Hydroxydecanoate | Lipid | Fatty Acid, Monohydroxy | HMDB0002203 | 7.351 | 187.13327 | [M-H]- | Negative_NH4HCO3 | 21.11 |
| 3-Hydroxylaurate | Lipid | Fatty Acid, Monohydroxy | HMDB0000387 | 7.554 | 215.16454 | [M-H]- | Negative_NH4HCO3 | 40.35 |
| 3-Hydroxyphenylalanine | Amino Acid | Phenylalanine Metabolism | HMDB0059720 | 2.585 | 180.06557 | [M-H]- | Negative_NH4HCO3 | 13.89 |
| 3-Indolepropionic Acid | Amino Acid | Tryptophan Metabolism | HMDB0002302 | 5.997 | 190.08589 | [M+H]+ | Positive | 2361.7 |
| 3-Indoxyl Sulfate | Amino Acid | Tryptophan Metabolism | HMDB0000682 | 3.785 | 212.00212 | [M-H]- | Negative_FA | 13.37 |
| 3-METHYL-2-OXINDOLE | Xenobiotics | Chemical | HMDB0304943 | 5.784 | 148.07553 | [M+H]+ | Positive | 33.47 |
| 3-METHYLCATECHOL | Xenobiotics | Benzoate Metabolism | HMDB0301753 | 3.131 | 123.04423 | [M-H]- | Negative_FA | 23.88 |
| 3-METHYLSALICYLIC ACID | Unclassified | Unclassified |  | 4.113 | 151.03909 | [M-H]- | Negative_FA | 289.61 |
| 3-Methyl-2-Oxobutyrate | Amino Acid | Leucine, Isoleucine and Valine Metabolism | HMDB0000019 | 2.263 | 115.03926 | [M-H]- | Negative_FA | 121.32 |
| 3-Methylbenzoic acid | Unclassified | Unclassified |  | 4.913 | 135.04407 | [M-H]- | Negative_FA | 36.69 |
| 3-Methylindole | Xenobiotics | Natural Product/Food/Plant | HMDB0000466 | 3.318 | 132.08072 | [M+H]+ | Positive | 11.11 |
| 3-O-Feruloylquinic acid | Unclassified | Unclassified |  | 4.538 | 367.10428 | [M-H]- | Negative_FA | 129046.6 |
| 3-Phenylpropionic Acid | Xenobiotics | Benzoate Metabolism | HMDB0000764 | 4.689 | 149.0596 | [M-H]- | Negative_NH4HCO3 | 3025.65 |
| 3-Sulfo-L-Alanine | Amino Acid | Methionine, Cysteine, SAM and Taurine Metabolism | HMDB0002757 | 0.91 | 167.99637 | [M-H]- | Negative_NH4HCO3 | 54.04 |
| 3-hydroxybenzaldehyde | Xenobiotics | Chemical |  | 1.313 | 121.02814 | [M-H]- | Negative_NH4HCO3 | 58.44 |
| 3_24_dihydroxyphenyl_propionic_acid | Unclassified | Unclassified |  | 4.912 | 181.04977 | [M-H]- | Negative_FA | 23.67 |
| 4-Aminohippuric acid | Xenobiotics | Benzoate Metabolism | HMDB0001867 | 3.053 | 195.07373 | [M+H]+ | Positive | 14.72 |
| 4-Guanidinobutanoate | Amino Acid | Urea cycle; Arginine and Proline Metabolism | HMDB0003464 | 1.239 | 146.09229 | [M+H]+ | Positive | 68.46 |
| 4-Hydroxyquinoline | Xenobiotics | Natural Product/Food/Plant | HMDB0246466 | 4.743 | 146.05995 | [M+H]+ | Positive | 27.25 |
| 4-Octylphenol | Xenobiotics | Chemical | HMDB0246557 | 7.967 | 205.15878 | [M-H]- | Negative_NH4HCO3 | 5.75 |
| 4-methyl-2-oxopentanoate | Amino Acid | Leucine, Isoleucine and Valine Metabolism | HMDB0000695 | 4.36 | 129.05466 | [M-H]- | Negative_FA | 369.38 |
| 5,6-Dihydrouracil | Nucleotide | Pyrimidine Metabolism, Uracil containing | HMDB0000076 | 1.282 | 115.03925 | [M+H]+ | Positive | 53.33 |
| 5-Carboxy-cytosine | Unclassified | Unclassified |  | 1.147 | 156.04196 | [M+H]+ | Positive | 382.44 |
| 5-Dodecenoic Acid (C12:1n7) | Lipid | Medium Chain Fatty Acid | HMDB0000529 | 7.729 | 197.15393 | [M-H]- | Negative_NH4HCO3 | 19.44 |
| 5-Hydroxyindole | Xenobiotics | Natural Product/Food/Plant | HMDB0059805 | 5.157 | 134.05995 | [M+H]+ | Positive | 16.39 |
| 5-Hydroxyindoleacetate | Amino Acid | Tryptophan Metabolism | HMDB0000763 | 4.821 | 192.06538 | [M+H]+ | Positive | 19.5 |
| 5-Methyluridine | Nucleotide | Pyrimidine Metabolism, Uracil containing | HMDB0000884 | 1.397 | 259.09235 | [M+H]+ | Positive | 9.88 |
| 5-Valerolactone | Xenobiotics | Chemical |  | 1.225 | 101.06009 | [M+H]+ | Positive | 497.19 |
| 6-Gingerol | Xenobiotics | Natural Product/Food/Plant | HMDB0005783 | 7.424 | 293.17581 | [M-H]- | Negative_NH4HCO3 | 380.93 |
| 6-Phosphonoglucono-D-lactone | Carbohydrate | Pentose phosphate pathway | HMDB0001127 | 0.901 | 257.00452 | [M-H]- | Negative_NH4HCO3 | 22.42 |
| 6-methoxypurine | Nucleotide | Purine and Pyrimidine Metabolism |  | 1.454 | 151.06923 | [M+H]+ | Positive | 32.79 |
| 7-Methylguanosine | Nucleotide | Purine Metabolism, Guanine containing | HMDB0001107 | 1.559 | 298.11438 | [M+K]+ | Positive | 38261.35 |
| 8-HETE | Unclassified | Unclassified |  | 6.918 | 319.22794 | [M-H]1- | Negative_FA | 2196302 |
| 9-HODE | Lipid | Fatty Acid Metabolism (Linolenic acid metabolism) | HMDB0004670 | 6.876 | 295.22763 | [M-H]1- | Negative_FA | 104532.8 |
| ALLOTHREONINE | Amino Acid | Glycine, Serine and Threonine Metabolism | HMDB00004041 | 0.995 | 118.0579 | [M-H]- | Negative_NH4HCO3 | 38.39 |
| ARABITOL | Unclassified | Unclassified |  | 1.279 | 175.06047 | [M+Na]+ | Negative_FA | 51.11 |
| Acesulfame | Xenobiotics | Natural Product/Food/Plant | HMDB0033585 | 1.901 | 161.98593 | [M-H]- | Negative_FA | 1553.47 |
| Aconitic Acid | Carbohydrate | Citrate cycle (TCA cycle) | HMDB0000072 | 0.836 | 172.99687 | [M-H]- | Negative_NH4HCO3 | 109.36 |
| Adenosine | Nucleotide | Purine Metabolism, Adenine containing | HMDB0000050 | 1.444 | 268.10336 | [M+H]+ | Positive | 157879.1 |
| Adipic acid | Xenobiotics | Degradation of aromatic compounds | HMDB0000448 | 3.054 | 145.04968 | [M-H]- | Negative_FA | 16.49 |
| Adrenic Acid (C22:4n6) | Lipid | Long Chain Fatty Acid | HMDB0002226 | 10.549 | 331.26755 | [M-H]- | Negative_NH4HCO3 | 20.84 |
| Ala-Ala | Peptide | Dipeptide | HMDB0003459 | 1.209 | 161.09196 | [M+H]+ | Positive | 55.68 |
| Ala-Ile | Peptide | Dipeptide | HMDB0028690 | 3.02 | 201.1236 | [M-H]- | Negative_FA | 39.69 |
| Ala-Leu | Peptide | Dipeptide | HMDB0028691 | 3.025 | 203.13895 | [M+H]+ | Positive | 115.68 |
| Ala-Phe | Peptide | Dipeptide | HMDB0028694 | 3.627 | 237.12311 | [M+H]+ | Positive | 38.86 |
| Ala-Val | Peptide | Dipeptide | HMDB0028700 | 1.603 | 189.12323 | [M+H]+ | Positive | 10264.74 |
| Allantoin | Nucleotide | Purine Metabolism, (Hypo)Xanthine/Inosine containing | HMDB0000462 | 0.928 | 157.03593 | [M-H]- | Negative_NH4HCO3 | 91.94 |
| Alpha-Aminobutyric Acid | Amino Acid | Methionine, Cysteine, SAM and Taurine Metabolism | HMDB0000452 | 1.013 | 102.05487 | [M-H]- | Negative_NH4HCO3 | 33.53 |
| Alpha-Hydroxyisocaproate | Amino Acid | Leucine, Isoleucine and Valine Metabolism | HMDB0000746 | 5.045 | 131.07027 | [M-H]- | Negative_FA | 1415.51 |
| Alpha-Ketobutyrate | Amino Acid | Methionine, Cysteine, SAM and Taurine Metabolism | HMDB0000005 | 1.06 | 101.02329 | [M-H]- | Negative_NH4HCO3 | 72.33 |
| Alpha/Gamma-Linolenic Acid (C18:3n3 or n6) | Lipid | Long Chain Fatty Acid | HMDB0001388 | 8.218 | 277.2179 | [M-H]- | Negative_NH4HCO3 | 19012.29 |
| Aminoadipic Acid | Amino Acid | Lysine Metabolism | HMDB0000510 | 1.361 | 160.06061 | [M-H]- | Negative_FA | 30.74 |
| Aminomalonic Acid | Amino Acid | Other Amino Acid Metabolism | HMDB0001147 | 1.616 | 118.022 | [M-H]- | Negative_FA | 114.61 |
| Aminovaleric acid | Unclassified | Unclassified |  | 2.761 | 118.08636 | [M+H]+ | Positive | 44.31 |
| Anthranilate | Amino Acid | Tryptophan Metabolism | HMDB0001123 | 5.038 | 120.04465 | [M+H-H2O]+ | Positive | 8.01 |
| Arachidonic Acid (C20:4n6) | Lipid | Long Chain Fatty Acid | HMDB0001043 | 8.409 | 303.23309 | [M-H]- | Negative_NH4HCO3 | 57.6 |
| Arg-Leu | Peptide | Dipeptide | HMDB0028713 | 1.65 | 288.20297 | [M+H]+ | Positive | 125719.2 |
| Argininosuccinic acid | Amino Acid | Urea cycle; Arginine and Proline Metabolism | HMDB0000052 | 0.83 | 291.12997 | [M+H]+ | Positive | 9.67 |
| Ascorbic Acid | Cofactors and Vitamins | Ascorbate and Aldarate Metabolism | HMDB0000044 | 0.824 | 175.02391 | [M-H]- | Negative_NH4HCO3 | 175.62 |
| Asp-Glu | Peptide | Dipeptide | HMDB0028752 | 1.199 | 263.08521 | [M+H]+ | Positive | 68482.01 |
| Asp-Leu | Unclassified | Unclassified |  | 3.151 | 247.12874 | [M+H]+ | Positive | 34.85 |
| Asp-Phe | Peptide | Dipeptide | HMDB0000706 | 3.635 | 281.11316 | [M+H]+ | Positive | 41.99 |
| Azacyclotridecan-2-one | Unclassified | Unclassified |  | 6.285 | 198.18475 | [M+H]+ | Positive | 1166.51 |
| Azelaic acid | Lipid | Medium Chain Fatty Acid | HMDB0000784 | 1.169 | 187.09657 | [M-H]- | Negative_NH4HCO3 | 36.61 |
| Azelate (C9-DC) | Lipid | Fatty Acid, Dicarboxylate | HMDB0000784 | 5.838 | 187.09698 | [M-H]- | Negative_FA | 263.86 |
| BENZYL ALCOHOL | Xenobiotics | Degradation of aromatic compounds | HMDB0003119 | 2.229 | 109.0651 | [M+H]+ | Positive | 8.33 |
| Benzoic acid | Xenobiotics | Benzoate Metabolism | HMDB0001870 | 1.48 | 123.0442 | [M+H]+ | Positive | 155.03 |
| Betaine | Amino Acid | Glycine, Serine and Threonine Metabolism | HMDB0000043 | 1.183 | 140.06802 | [M+Na]+ | Positive | 174.22 |
| Biliverdin | Cofactors and Vitamins | Hemoglobin and Porphyrin Metabolism | HMDB0001008 | 6.48 | 583.25513 | [M+H]+ | Positive | 516446.1 |
| Butyrylcarnitine | Lipid | Fatty Acid Metabolism (Acyl Carnitine) | HMDB0002013 | 2.861 | 232.15437 | [M+H]+ | Positive | 20.01 |
| C-Glycosyltryptophan | Amino Acid | Tryptophan Metabolism | HMDB0240296 | 1.975 | 367.14923 | [M+H]+ | Positive | 27914.4 |
| C12-AS (TENTATIVE) | Unclassified | Unclassified |  | 6.773 | 265.14679 | [M-H]- | Negative_FA | 11318.13 |
| Cadaverine | Amino Acid | Lysine Metabolism | HMDB0002322 | 1.057 | 103.13151 | [M+H]+ | Positive | 108.55 |
| Caffeic Acid | Xenobiotics | Natural Product/Food/Plant | HMDB0001964 | 4.433 | 179.03424 | [M-H]- | Negative_FA | 38.14 |
| Caffeine | Xenobiotics | Xanthine Metabolism | HMDB0001847 | 3.932 | 195.08745 | [M+H]+ | Positive | 1080.86 |
| Caproic Acid (C6:0)/4-methylvaleric acid | Lipid | Medium Chain Fatty Acid | HMDB0000535 | 3.961 | 115.07525 | [M-H]- | Negative_NH4HCO3 | 900.22 |
| Carboxyethyl-GABA | Amino Acid | Alanine, Aspartate and Glutamate Metabolism | HMDB0002201 | 1.358 | 176.09157 | [M+H]+ | Positive | 100.66 |
| Carnitine | Lipid | Fatty Acid Metabolism (Acyl Carnitine) | HMDB0000062 | 1.179 | 162.11256 | [M+H]+ | Positive | 33499.45 |
| Carnosine | Peptide | Hybrid peptide | HMDB0000033 | 1.138 | 227.11357 | [M+H]+ | Positive | 21.86 |
| Catechol Sulfate | Xenobiotics | Benzoate Metabolism | HMDB0059724 | 2.363 | 188.98526 | [M-H]- | Negative_NH4HCO3 | 21.43 |
| Cholic Acid | Lipid | Bile Acid | HMDB0000619 | 6.653 | 407.27963 | [M-H]- | Negative_FA | 133876.4 |
| Choline | Lipid | Glycerolipid and Phospholipid Metabolism | HMDB0000097 | 1.138 | 104.10737 | [M]+ | Positive | 1304.88 |
| Choline Phosphate | Lipid | Glycerolipid and Phospholipid Metabolism | HMDB0001565 | 1.14 | 184.07332 | [M]+ | Positive | 26.22 |
| Cinnamic Acid | Amino Acid | Phenylalanine Metabolism | HMDB0000567 | 2.289 | 149.05952 | [M+H]+ | Positive | 28.16 |
| Citraconic Acid | Lipid | Fatty Acid, Dicarboxylate | HMDB0000634 | 0.894 | 129.01817 | [M-H]- | Negative_NH4HCO3 | 153.88 |
| Citric Acid/Isocitric Acid | Carbohydrate | Citrate cycle (TCA cycle) | HMDB0000193 | 0.847 | 191.01854 | [M-H]- | Negative_NH4HCO3 | 106.17 |
| Citrulline | Amino Acid | Urea cycle; Arginine and Proline Metabolism | HMDB0000904 | 1.23 | 159.07619 | [M+H-NH3]+ | Positive | 26.66 |
| Cortisol | Lipid | Corticosteroids | HMDB0000063 | 5.647 | 363.19455 | [M+H]+ | Positive | 16.15 |
| Cortisone | Lipid | Corticosteroids | HMDB0002802 | 6.048 | 361.20004 | [M+H]+ | Positive | 11.73 |
| Cotinine | Xenobiotics | Tobaco Metabolite | HMDB0001046 | 1.435 | 177.10211 | [M+H]+ | Positive | 489.29 |
| Creatine | Amino Acid | Creatine Metabolism | HMDB0000064 | 1.244 | 132.08072 | [M+H]+ | Positive | 338.9 |
| Creatinine | Amino Acid | Creatine Metabolism | HMDB0000562 | 1.191 | 114.06644 | [M+H]+ | Positive | 99.2 |
| Cyclo(L-Val-L-Pro) | Peptide | Dipeptide | HMDB0240493 | 3.932 | 197.12804 | [M+H]+ | Positive | 28 |
| Cyclo(Leu-Pro) | Peptide | Dipeptide | HMDB0034276 | 5.012 | 211.14375 | [M+H]+ | Positive | 27.58 |
| Cysteine S-sulfate | Amino Acid | Methionine, Cysteine, SAM and Taurine Metabolism | HMDB0000731 | 0.909 | 199.96869 | [M-H]- | Negative_NH4HCO3 | 18.22 |
| Cystine | Amino Acid | Methionine, Cysteine, SAM and Taurine Metabolism | HMDB0000192 | 0.944 | 239.01114 | [M-H]- | Negative_NH4HCO3 | 86.93 |
| Cytosine | Nucleotide | Pyrimidine Metabolism, Cytidine containing | HMDB0000630 | 1.24 | 112.05093 | [M+H]+ | Positive | 8.63 |
| D-Gluconic Acid | Carbohydrate | Pentose phosphate pathway | HMDB0000625 | 0.927 | 195.05 | [M-H]- | Negative_NH4HCO3 | 163.19 |
| D-Ribulose 1,5-Bisphosphate | Carbohydrate | Pentose phosphate pathway | HMDB0304322 | 0.94 | 308.97015 | [M-H]- | Negative_NH4HCO3 | 85.13 |
| D-Ribulose 5-Phosphate | Carbohydrate | Pentose phosphate pathway | HMDB0000618 | 0.9 | 229.01166 | [M-H]- | Negative_NH4HCO3 | 30.94 |
| DEHP | Xenobiotics | Chemical | HMDB0249243 | 7.95 | 413.2662 | [M+H]+ | Positive | 379178.5 |
| Daidzein | Xenobiotics | Natural Product/Food/Plant | HMDB0003312 | 5.934 | 255.06369 | [M+H]+ | Positive | 65.76 |
| Decanoic Acid (C10:0) | Lipid | Medium Chain Fatty Acid | HMDB0000511 | 7.589 | 171.13844 | [M-H]- | Negative_NH4HCO3 | 79.63 |
| Decenoate (10:1) | Lipid | Medium Chain Fatty Acid | HMDB0041012 | 7.499 | 169.12234 | [M-H]- | Negative_NH4HCO3 | 16.71 |
| Decenoylcarnitine | Lipid | Fatty Acid Metabolism (Acyl Carnitine) | HMDB0241072 | 6.556 | 314.24033 | [M+H]+ | Positive | 108698.1 |
| Dehydrocorydaline | Unclassified | Unclassified |  | 5.9 | 366.16913 | [M]+ | Positive | 75287.04 |
| Dehydroepiandrosterone Sulfate (DHEA-S) | Lipid | Androgenic Steroids | HMDB0001032 | 7.356 | 367.15851 | [M-H]- | Negative_NH4HCO3 | 22372.6 |
| Deoxyadenosine | Nucleotide | Purine Metabolism, Adenine containing | HMDB0000101 | 1.44 | 252.10875 | [M+H]+ | Positive | 246518.1 |
| Deoxycarnitine | Lipid | Fatty Acid Metabolism (Acyl Carnitine) | HMDB0001161 | 1.188 | 146.11742 | [M+H]+ | Positive | 655.09 |
| Deoxycholic Acid | Lipid | Bile Acid | HMDB0000626 | 6.913 | 391.28925 | [M-H]- | Negative_FA | 2168491 |
| Deoxycytidine | Nucleotide | Pyrimidine Metabolism, Cytidine containing | HMDB0000014 | 1.248 | 228.09755 | [M+H]+ | Positive | 21.88 |
| Deoxyguanosine | Nucleotide | Purine Metabolism, Guanine containing | HMDB0000085 | 0.923 | 266.00412 | [M-H]- | Negative_NH4HCO3 | 58.69 |
| Diaveridine | Unclassified | Unclassified |  | 3.182 | 261.13031 | [M+H]+ | Positive | 93.12 |
| Diethyl-phthalate | Unclassified | Unclassified |  | 6.318 | 221.08147 | [M-H]- | Negative_FA | 139.75 |
| Dimethyl Sulfoxide | Xenobiotics | Chemical | HMDB0002151 | 0.97 | 79.01849 | [M+H]+ | Positive | 295.76 |
| Dimethylarginine (SDMA + ADMA) | Amino Acid | Urea cycle; Arginine and Proline Metabolism | HMDB0001539 | 1.163 | 203.15009 | [M+H]+ | Positive | 4957.87 |
| Dl-Coniine | Xenobiotics | Natural Product/Food/Plant | HMDB0030285 | 2.354 | 128.14339 | [M+H]+ | Positive | 4.46 |
| Dodecanedioate (C12-DC) | Lipid | Fatty Acid, Dicarboxylate | HMDB0000623 | 6.382 | 229.14404 | [M-H]- | Negative_FA | 70573.15 |
| Dodecanoic Acid (C12:0) | Lipid | Medium Chain Fatty Acid | HMDB0000638 | 7.875 | 199.16933 | [M-H]- | Negative_NH4HCO3 | 144.42 |
| Dodecylbenzenesulfonic acid | Xenobiotics | Chemical | HMDB0031031 | 7.051 | 325.18329 | [M-H]- | Negative_FA | 154.46 |
| Ectoine | Xenobiotics | Natural Product/Food/Plant | METPA0797 | 1.262 | 143.08163 | [M+H]+ | Positive | 41.16 |
| Eicosanedioate (C20-DC) | Lipid | Fatty Acid, Dicarboxylate | HMDB0242141 | 7.406 | 341.2695 | [M-H]- | Negative_FA | 118573.9 |
| Eicosapentaenoic Acid (C20:5n3 EPA) | Lipid | Long Chain Fatty Acid | HMDB0001999 | 7.897 | 301.21732 | [M-H]- | Negative_NH4HCO3 | 14.47 |
| Ethanolamine | Lipid | Glycerolipid and Phospholipid Metabolism | HMDB0000149 | 1.143 | 62.06081 | [M+H]+ | Positive | 93.43 |
| Ethylmalonic acid | Lipid | Medium Chain Fatty Acid | HMDB0000622 | 2.544 | 131.03394 | [M-H]- | Negative_FA | 41.39 |
| FA 18:2+2O | Lipid | Fatty Acid Metabolism ( Oxidized fatty acid) |  | 7.587 | 311.22214 | [M-H]- | Negative_NH4HCO3 | 79.12 |
| Formylkynurenine | Amino Acid | Tryptophan Metabolism | HMDB0001200 | 1.998 | 237.08707 | [M+H]+ | Positive | 17.41 |
| Fructose 1,6-Bisphosphate | Carbohydrate | Glycolysis, Gluconeogenesis, and Pyruvate Metabolism | HMDB0001058 | 0.897 | 338.98819 | [M-H]- | Negative_NH4HCO3 | 14260.25 |
| Fumaric Acid | Carbohydrate | Citrate cycle (TCA cycle) | HMDB0000134 | 0.903 | 115.00015 | [M-H]- | Negative_NH4HCO3 | 123.95 |
| Gamma-Aminobutyric Acid | Amino Acid | Alanine, Aspartate and Glutamate Metabolism | HMDB0000112 | 1.196 | 104.071 | [M+H]+ | Positive | 49.9 |
| Gamma-Glutamyl-Cysteine | Peptide | Gamma-glutamyl Amino Acid | HMDB0001049 | 1.442 | 251.05406 | [M+H]+ | Positive | 28.23 |
| Gamma-Glutamylalanine | Peptide | Gamma-glutamyl Amino Acid | HMDB0006248 | 1.139 | 219.09747 | [M+H]+ | Positive | 19.4 |
| Gamma-Glutamylglutamate | Peptide | Gamma-glutamyl Amino Acid | HMDB0011737 | 0.723 | 277.10422 | [M+H]+ | Positive | 13.81 |
| Gamma-Glutamylleucine | Peptide | Gamma-glutamyl Amino Acid | HMDB0011171 | 2.767 | 261.14407 | [M+H]+ | Positive | 15.19 |
| Gamma-Glutamylphenylalanine | Peptide | Gamma-glutamyl Amino Acid | HMDB0000594 | 3.878 | 295.12817 | [M+H]+ | Positive | 31.64 |
| Gamma-Glutamyltyrosine | Peptide | Gamma-glutamyl Amino Acid | HMDB0011741 | 1.728 | 311.1236 | [M+H]+ | Positive | 32.98 |
| Genistein | Xenobiotics | Natural Product/Food/Plant | HMDB0003217 | 6.139 | 269.05676 | [M-H]- | Negative_FA | 562311.3 |
| Gentisate | Amino Acid | Tyrosine Metabolism | HMDB0000152 | 1.447 | 153.01807 | [M-H]- | Negative_NH4HCO3 | 70.86 |
| Gln-Leu | Unclassified | Unclassified |  | 2.884 | 260.16034 | [M+H]+ | Positive | 36.67 |
| Glu-Pro | Unclassified | Unclassified |  | 1.291 | 227.09999 | [M-H2O+H]+ | Positive | 17.95 |
| Glucosamine 6-Phosphate | Carbohydrate | Amino sugar and Nucleotide sugar Metabolism | HMDB0001254 | 0.909 | 258.0289 | [M-H]- | Negative_NH4HCO3 | 48.56 |
| Glucose | Carbohydrate | Glycolysis, Gluconeogenesis, and Pyruvate Metabolism | HMDB0000122 | 0.965 | 179.05515 | [M-H]- | Negative_NH4HCO3 | 708.64 |
| Glucose 6-Phosphate/Fructose 6-Phosphate | Carbohydrate | Glycolysis, Gluconeogenesis, and Pyruvate Metabolism | HMDB0001401 | 0.893 | 259.02213 | [M-H]- | Negative_NH4HCO3 | 83.47 |
| Glucuronate | Carbohydrate | Amino sugar and Nucleotide sugar Metabolism | HMDB0000127 | 0.921 | 193.03458 | [M-H]- | Negative_NH4HCO3 | 50.65 |
| Glutarate (C5-DC) | Lipid | Fatty Acid, Dicarboxylate | HMDB0000661 | 1.949 | 131.03406 | [M-H]- | Negative_FA | 89.99 |
| Gly-Leu | Peptide | Dipeptide | HMDB0000759 | 2.89 | 189.12338 | [M+H]+ | Positive | 7232.39 |
| Gly-Phe | Peptide | Dipeptide | HMDB0028848 | 3.534 | 223.10725 | [M+H]+ | Positive | 55.42 |
| Gly-Pro | Unclassified | Unclassified |  | 1.272 | 173.09203 | [M+H]+ | Positive | 99.08 |
| Gly-Trp | Peptide | Dipeptide | HMDB0028852 | 4.079 | 262.11853 | [M+H]+ | Positive | 10.42 |
| Gly-Tyr | Peptide | Dipeptide | HMDB0028853 | 1.726 | 239.10245 | [M+H]+ | Positive | 92.35 |
| Gly-Val | Peptide | Dipeptide | HMDB0028854 | 1.574 | 175.10773 | [M+H]+ | Positive | 57.52 |
| Glyceraldehyde 3-Phosphate | Carbohydrate | Glycolysis, Gluconeogenesis, and Pyruvate Metabolism | HMDB0001112 | 0.884 | 168.98042 | [M-H]- | Negative_NH4HCO3 | 49.89 |
| Glyceric Acid | Carbohydrate | Pentose phosphate pathway | HMDB0000139 | 1.119 | 105.01825 | [M-H]- | Negative_FA | 41.57 |
| Glycerol | Lipid | Glycerolipid and Phospholipid Metabolism | HMDB0000131 | 2.057 | 91.02092 | [M-H]- | Negative_NH4HCO3 | 25.84 |
| Glycerol 3-Phosphate | Lipid | Glycerolipid and Phospholipid Metabolism | HMDB0000126 | 1.14 | 171.00563 | [M-H]- | Negative_FA | 22.3 |
| Glycerophosphoethanolamine | Lipid | Glycerolipid and Phospholipid Metabolism | HMDB0000114 | 0.969 | 214.04762 | [M-H]- | Negative_NH4HCO3 | 25.9 |
| Glycerophosphoglycerol | Lipid | Glycerolipid and Phospholipid Metabolism | HMDB0240316 | 0.946 | 245.05389 | [M-H]- | Negative_NH4HCO3 | 73.76 |
| Glycerophosphoinositol | Lipid | Glycerolipid and Phospholipid Metabolism | HMDB0011649 | 0.912 | 333.06317 | [M-H]- | Negative_NH4HCO3 | 115983.6 |
| Glycine | Amino Acid | Glycine, Serine and Threonine Metabolism | HMDB0000123 | 1.184 | 76.03999 | [M+H]+ | Positive | 12.05 |
| Glycocholic Acid | Lipid | Bile Acid, glycine conjungate | HMDB0000138 | 6.511 | 464.30215 | [M-H]- | Negative_FA | 213571 |
| Glycodeoxycholic Acid | Lipid | Bile Acid, glycine conjungate | HMDB0000631 | 6.677 | 448.30676 | [M-H]- | Negative_FA | 295025.7 |
| Glycolic Acid | Carbohydrate | Glyoxylate and dicarboxylate metabolism | HMDB0000115 | 0.955 | 74.96084 | [M-H]- | Negative_NH4HCO3 | 7.08 |
| Glycyrrhizin | Unclassified | Unclassified |  | 6.421 | 823.4101 | [M+H]+ | Positive | 283922.6 |
| Guanidinoacetic acid | Amino Acid | Creatine Metabolism | HMDB0000128 | 2.282 | 118.06542 | [M+H]+ | Positive | 12.07 |
| Guanosine | Nucleotide | Purine Metabolism, Guanine containing | HMDB0000133 | 1.457 | 284.09995 | [M+H]+ | Positive | 195363.1 |
| Heptadecanoic Acid (C17:0) | Lipid | Long Chain Fatty Acid | HMDB0002259 | 8.869 | 269.24844 | [M-H]- | Negative_NH4HCO3 | 42.8 |
| Heptanoic Acid (C7:0) | Lipid | Medium Chain Fatty Acid | HMDB0000666 | 6.634 | 129.09106 | [M-H]- | Negative_NH4HCO3 | 11.64 |
| Hesperetin | Xenobiotics | Natural Product/Food/Plant | HMDB0005782 | 6.75 | 301.07129 | [M-H]- | Negative_NH4HCO3 | 111.57 |
| Hexaconazole | Unclassified | Unclassified |  | 1.27 | 314.08438 | [M+H]+ | Positive | 651888.9 |
| Hexadecanedioate (C16-DC) | Lipid | Fatty Acid, Dicarboxylate | HMDB0000672 | 6.616 | 285.207 | [M-H]- | Negative_FA | 174088.7 |
| Hexanoylcarnitine | Lipid | Fatty Acid Metabolism (Acyl Carnitine) | HMDB0000756 | 5.427 | 260.18564 | [M+H]+ | Positive | 23034.18 |
| His-Leu | Peptide | Dipeptide | HMDB0028889 | 1.551 | 269.16071 | [M+H]+ | Positive | 51.78 |
| His-Ser | Unclassified | Unclassified |  | 1.026 | 243.10855 | [M+H]+ | Positive | 20.52 |
| Histamine | Amino Acid | Histidine Metabolism | HMDB0000870 | 1.065 | 112.0872 | [M+H]+ | Positive | 65.32 |
| Homovanillic Acid | Amino Acid | Tyrosine Metabolism | HMDB0000118 | 3.317 | 181.0502 | [M-H]- | Negative_FA | 88.97 |
| Hydroxy-N6,N6,N6-Trimethyllysine | Amino Acid | Lysine Metabolism | HMDB0001422 | 1.062 | 205.15451 | [M+H]+ | Positive | 19.6 |
| Hydroxyhexanoycarnitine | Lipid | Fatty Acid Metabolism (Acyl Carnitine) | HMDB0013131 | 1.674 | 276.14383 | [M+H]+ | Positive | 25311.1 |
| Hydroxyphenyllactic Acid | Amino Acid | Tyrosine Metabolism | HMDB0000755 | 1.304 | 181.04976 | [M-H]- | Negative_NH4HCO3 | 1109.38 |
| Hydroxypyruvic Acid | Amino Acid | Glycine, Serine and Threonine Metabolism | HMDB0001352 | 0.874 | 103.00246 | [M-H]- | Negative_NH4HCO3 | 228.84 |
| Hydroxysuberic acid | Lipid | Fatty Acid, Monohydroxy | HMDB0000325 | 5.324 | 189.07622 | [M-H]- | Negative_FA | 59.25 |
| Hypotaurine | Amino Acid | Methionine, Cysteine, SAM and Taurine Metabolism | HMDB0000965 | 1.312 | 108.02039 | [M-H]- | Negative_NH4HCO3 | 26.79 |
| Hypoxanthine | Nucleotide | Purine Metabolism, (Hypo)Xanthine/Inosine containing | HMDB0000157 | 1.418 | 137.0457 | [M+H]+ | Positive | 129.39 |
| Ile-Ala | Peptide | Dipeptide | HMDB0028900 | 1.73 | 203.13875 | [M+H]+ | Positive | 25346.02 |
| Ile-Asp | Unclassified | Unclassified |  | 1.539 | 247.12866 | [M+H]+ | Positive | 68.91 |
| Ile-Gln | Unclassified | Unclassified |  | 1.494 | 260.16028 | [M+H]+ | Positive | 18548.64 |
| Ile-Tyr | Peptide | Dipeptide | HMDB0028919 | 3.557 | 295.16528 | [M+H]+ | Positive | 47.8 |
| Indole-3-acetaldehyde | Amino Acid | Tryptophan Metabolism | HMDB0001190 | 5.421 | 160.07538 | [M+H]+ | Positive | 10.31 |
| Indole-3-carboxyaldehyde | Amino Acid | Tryptophan Metabolism |  | 5.558 | 146.06003 | [M+H]+ | Positive | 34.96 |
| Indole-3-carboxylic Acid | Amino Acid | Tryptophan Metabolism | HMDB0003320 | 5.535 | 162.05469 | [M+H]+ | Positive | 10.57 |
| Indole-3-ethanol | Xenobiotics | Natural Product/Food/Plant | HMDB0003447 | 5.754 | 162.09111 | [M+H]+ | Positive | 18.36 |
| Indoleacetic Acid | Amino Acid | Tryptophan Metabolism | HMDB0000197 | 5.749 | 176.0704 | [M+H]+ | Positive | 800.08 |
| Indolelactate | Amino Acid | Tryptophan Metabolism | HMDB0000671 | 5.453 | 206.08067 | [M+H]+ | Positive | 69.26 |
| Isovaleric Acid | Amino Acid | Leucine, Isoleucine and Valine Metabolism | HMDB0000718 | 1.752 | 101.0596 | [M-H]- | Negative_NH4HCO3 | 1408.96 |
| Ketoisovaleric Acid | Amino Acid | Leucine, Isoleucine and Valine Metabolism | HMDB0000019 | 1.5 | 115.03899 | [M-H]- | Negative_NH4HCO3 | 16.54 |
| Kynurenate | Amino Acid | Tryptophan Metabolism | HMDB0000715 | 3.669 | 190.04977 | [M+H]+ | Positive | 9.52 |
| L-Acetylcarnitine | Lipid | Fatty Acid Metabolism (Acyl Carnitine) | HMDB0000201 | 1.273 | 204.12297 | [M+H]+ | Positive | 614769.3 |
| L-Alanine | Amino Acid | Alanine, Aspartate and Glutamate Metabolism | HMDB0000161 | 1.214 | 90.04562 | [M+H]+ | Positive | 27.93 |
| L-Allothreonine | Amino Acid | Other Amino Acid Metabolism | HMDB0004041 | 1.049 | 164.02916 | [M+H]+ | Positive | 10.53 |
| L-Arginine | Amino Acid | Urea cycle; Arginine and Proline Metabolism | HMDB0000517 | 1.131 | 175.1075 | [M+H]+ | Positive | 110.71 |
| L-Aspartic Acid | Amino Acid | Alanine, Aspartate and Glutamate Metabolism | HMDB0000191 | 1.184 | 132.02936 | [M-H]- | Negative_FA | 35.06 |
| L-Glutamic Acid | Amino Acid | Alanine, Aspartate and Glutamate Metabolism | HMDB0000148 | 1.226 | 148.04726 | [M+H]+ | Positive | 218.52 |
| L-Glutamine | Amino Acid | Alanine, Aspartate and Glutamate Metabolism | HMDB0000641 | 1.094 | 147.07138 | [M+H]+ | Positive | 115.18 |
| L-Histidine | Amino Acid | Histidine Metabolism | HMDB0000177 | 1.086 | 156.07664 | [M+H]+ | Positive | 291.31 |
| L-Homoserine | Amino Acid | Other Amino Acid Metabolism | HMDB0000719 | 1.287 | 118.05793 | [M-H]- | Negative_NH4HCO3 | 30.51 |
| L-Kynurenine | Amino Acid | Tryptophan Metabolism | HMDB0000684 | 2.085 | 209.09198 | [M+H]+ | Positive | 14.23 |
| L-Leucine | Amino Acid | Leucine, Isoleucine and Valine Metabolism | HMDB0000687 | 1.736 | 132.10193 | [M+H]+ | Positive | 2529.41 |
| L-Lysine | Amino Acid | Lysine Metabolism | HMDB0000182 | 1.044 | 147.11267 | [M+H]+ | Positive | 489.28 |
| L-Methionine | Amino Acid | Methionine, Cysteine, SAM and Taurine Metabolism | HMDB0000696 | 1.453 | 150.06277 | [M+H]+ | Positive | 88.22 |
| L-Norvaline | Amino Acid | Other Amino Acid Metabolism | HMDB0013716 | 1.003 | 116.0706 | [M-H]- | Negative_NH4HCO3 | 1238.16 |
| L-Phenylalanine | Amino Acid | Phenylalanine Metabolism | HMDB0000159 | 2.292 | 166.08617 | [M+H]+ | Positive | 8889.02 |
| L-Pipecolic Acid | Amino Acid | Lysine Metabolism | HMDB0000716 | 1.216 | 130.08655 | [M+H]+ | Positive | 124.02 |
| L-Proline | Amino Acid | Urea cycle; Arginine and Proline Metabolism | HMDB0000162 | 1.211 | 116.07075 | [M+H]+ | Positive | 1427.75 |
| L-Prolyl-L-isoleucine | Peptide | Dipeptide |  | 1.347 | 229.15448 | [M+Li]+ | Positive | 28.23 |
| L-Serine | Amino Acid | Glycine, Serine and Threonine Metabolism | HMDB0000187 | 1.308 | 106.05022 | [M+H]+ | Positive | 20.34 |
| L-Threonine | Amino Acid | Glycine, Serine and Threonine Metabolism | HMDB0000167 | 1.053 | 120.06558 | [M+H]+ | Positive | 12.9 |
| L-Tryptophan | Amino Acid | Tryptophan Metabolism | HMDB0000929 | 3.434 | 205.09688 | [M+H]+ | Positive | 436.15 |
| L-Tyrosine | Amino Acid | Tyrosine Metabolism | HMDB0000158 | 1.465 | 182.08113 | [M+H]+ | Positive | 1545.4 |
| L-Valine | Amino Acid | Leucine, Isoleucine and Valine Metabolism | HMDB0000883 | 1.22 | 118.08632 | [M+H]+ | Positive | 3513.49 |
| L-methionine sulfoxide | Amino Acid | Methionine, Cysteine, SAM and Taurine Metabolism | HMDB0002005 | 1.441 | 166.05309 | [M+H]+ | Positive | 18.24 |
| LPC 18:0_sn1 | Lipid | Glycerophospholipid (lysophosphatidylcholine) | HMDB0010384 | 7.588 | 524.37085 | [M+H]+ | Positive | 144093.7 |
| LPC 18:1_sn1 | Lipid | Glycerophospholipid (lysophosphatidylcholine) | HMDB0002815 | 7.333 | 522.35474 | [M+H]+ | Positive | 247325.7 |
| LPC O-18:0 | Unclassified | Unclassified |  | 7.888 | 510.39148 | [M+H]+ | Positive | 18 |
| LPE 16:1_sn1 | Lipid | Glycerophospholipid (lysophosphatidylethanolamine) | HMDB0011504 | 7.118 | 452.27692 | [M+H]+ | Positive | 137769.7 |
| LPE 18:1_sn1 | Lipid | Glycerophospholipid (lysophosphatidylethanolamine) | HMDB0011505 | 7.362 | 480.30847 | [M+H]+ | Positive | 261789.4 |
| LPE P-16:0 | Lipid | Glycerophospholipid (lysophosphatidylethanolamine) | HMDB0011152 | 7.578 | 438.29868 | [M+H]+ | Positive | 8.26 |
| LPE P-18:0 | Lipid | Glycerophospholipid (lysophosphatidylethanolamine) | HMDB0240598 | 7.834 | 466.32834 | [M+H]+ | Positive | 221887.8 |
| LPE P-18:1 | Lipid | Glycerophospholipid (lysophosphatidylethanolamine) | HMDB0240599 | 7.415 | 464.3136 | [M+H]+ | Positive | 146988.4 |
| LPG 18:1 | Lipid | Glycerophospholipid (lysophosphatidylglycerol) | HMDB0240602 | 8.358 | 509.29752 | [M-H]- | Negative_NH4HCO3 | 270536.4 |
| Lacosamide | Xenobiotics | Drug | HMDB0253940 | 6.451 | 249.13382 | [M-H]- | Negative_NH4HCO3 | 4.85 |
| Lactic Acid | Carbohydrate | Glycolysis, Gluconeogenesis, and Pyruvate Metabolism | HMDB0000190 | 0.963 | 89.02321 | [M-H]- | Negative_NH4HCO3 | 1198.12 |
| Lactoylphenylalanine | Peptide | Pseudodipeptides (N-Lactoyl-amino acids ) | HMDB0062175 | 5.549 | 238.10745 | [M+H]+ | Positive | 8.46 |
| Lauryl Sulfate | Xenobiotics | Natural Product/Food/Plant |  | 7.972 | 265.14731 | [M-H]- | Negative_NH4HCO3 | 3621.93 |
| Lauryl diethanolamide | Lipid | Endocannabinoid (N-acylethanolamine) | HMDB0032358 | 6.746 | 288.25305 | [M+H]+ | Positive | 116457.1 |
| Leu-Ala | Peptide | Dipeptide | HMDB0028922 | 1.75 | 203.13898 | [M+H]+ | Positive | 210.19 |
| Leu-Glu | Peptide | Dipeptide | HMDB0028928 | 1.711 | 261.13885 | [M+H]+ | Positive | 19.29 |
| Leu-Gly-Gly | Peptide | Small peptide |  | 1.451 | 246.1413 | [M+H]+ | Positive | 206795.3 |
| Leu-Ile | Peptide | Dipeptide | HMDB0028932 | 4.853 | 245.18613 | [M+H]+ | Positive | 1979022 |
| Leu-Trp | Peptide | Dipeptide | HMDB0028940 | 5.42 | 318.18097 | [M+H]+ | Positive | 27.17 |
| Linoleic Acid (C18:2n6) | Lipid | Long Chain Fatty Acid | HMDB0000673 | 8.407 | 279.2319 | [M-H]- | Negative_NH4HCO3 | 167.14 |
| Linoleoyl ethanolamide | Lipid | Endocannabinoid (N-acylethanolamine) | HMDB0012252 | 7.122 | 324.28915 | [M+H]+ | Positive | 190989.6 |
| Liquiritigenin | Unclassified | Unclassified |  | 5.876 | 255.06604 | [M-H]- | Negative_FA | 107380.4 |
| Lumichrome | Xenobiotics | Natural Product/Food/Plant | HMDB0254199 | 5.783 | 243.08746 | [M+H]+ | Positive | 28.57 |
| Lys-Ile | Peptide | Dipeptide | HMDB0028954 | 1.625 | 260.1965 | [M+H]+ | Positive | 178851.8 |
| Malic Acid | Carbohydrate | Citrate cycle (TCA cycle) | HMDB0000156 | 0.875 | 133.01308 | [M-H]- | Negative_NH4HCO3 | 709.91 |
| Malonic Acid (C3-DC) | Lipid | Fatty Acid, Dicarboxylate | HMDB0000691 | 1.27 | 103.00266 | [M-H]- | Negative_FA | 115.46 |
| Malonylcarnitine | Lipid | Fatty Acid Metabolism (Acyl Carnitine) | HMDB0002095 | 1.337 | 248.11284 | [M+H]+ | Positive | 87.49 |
| Mannitol/Sorbitol | Carbohydrate | Polysaccharides and Oligosaccharides | HMDB0000247 | 1.002 | 181.07074 | [M-H]- | Negative_NH4HCO3 | 87.99 |
| Mannose | Carbohydrate | Polysaccharides and Oligosaccharides | HMDB0000169 | 1.212 | 179.05545 | [M-H]- | Negative_FA | 376.96 |
| Met-Ile | Unclassified | Unclassified |  | 3.701 | 263.13876 | [M+H]+ | Positive | 17.69 |
| Met-Leu | Peptide | Dipeptide | HMDB0028977 | 4.453 | 263.13873 | [M+H]+ | Positive | 40.24 |
| Met-Val | Peptide | Dipeptide | HMDB0028986 | 2.821 | 249.12651 | [M+H]+ | Positive | 10.72 |
| Methylergonovine | Unclassified | Unclassified |  | 5.647 | 340.20462 | [M+H]+ | Positive | 100145.6 |
| Methylhistamine | Amino Acid | Histidine Metabolism | HMDB0000898 | 1.065 | 126.10821 | [M+H]+ | Positive | 24.39 |
| Methylimidazoleacetic acid | Amino Acid | Histidine Metabolism | HMDB0002820 | 1.237 | 141.06569 | [M+H]+ | Positive | 396.34 |
| Methylsuccinic Acid | Amino Acid | Leucine, Isoleucine and Valine Metabolism | HMDB0001844 | 0.891 | 131.03403 | [M-H]- | Negative_NH4HCO3 | 131.52 |
| Metoprolol acid | Xenobiotics | Drug | HMDB0254682 | 5.359 | 268.19034 | [M+H]+ | Positive | 602.7 |
| Myristic Acid (C14:0) | Lipid | Long Chain Fatty Acid | HMDB0000806 | 8.184 | 227.20097 | [M-H]- | Negative_NH4HCO3 | 241.3 |
| Myristoylglycine | Lipid | Fatty Acid Metabolism (Acyl glycines) | HMDB0013250 | 7.045 | 284.22253 | [M-H]- | Negative_FA | 131702.3 |
| N,N,N-Trimethyl-5-Aminovalerate | Amino Acid | Lysine Metabolism | HMDB0240732 | 1.229 | 160.13295 | [M+H]+ | Positive | 20813.06 |
| N,N-Dimethyl-Proline-Proline Betaine | Amino Acid | Urea cycle; Arginine and Proline Metabolism |  | 1.443 | 241.15453 | [M+H]+ | Positive | 25.79 |
| N,N-Dimethyltetradecylamine | Unclassified | Unclassified |  | 7.681 | 242.28389 | [M+H]+ | Positive | 257643.4 |
| N-Acetyl-D-Glucosamine | Carbohydrate | Amino sugar and Nucleotide sugar Metabolism | HMDB0000215 | 1.263 | 222.09721 | [M+H]+ | Positive | 7.63 |
| N-Acetyl-D-Mannosamine | Carbohydrate | Amino sugar and Nucleotide sugar Metabolism | HMDB0001129 | 1.201 | 244.07892 | [M+Na]+ | Positive | 127326.9 |
| N-Acetyl-L-Aspartic Acid | Amino Acid | Alanine, Aspartate and Glutamate Metabolism | HMDB0000812 | 1.3 | 174.04051 | [M-H]- | Negative_FA | 158.41 |
| N-Acetyl-L-Tyrosine | Amino Acid | Tyrosine Metabolism | HMDB0000866 | 3.394 | 222.07657 | [M-H]- | Negative_FA | 39.38 |
| N-Acetylcadaverine | Amino Acid | Lysine Metabolism | HMDB0002284 | 1.283 | 145.13347 | [M+H]+ | Positive | 731.1 |
| N-Acetylcitrulline | Amino Acid | Urea cycle; Arginine and Proline Metabolism | HMDB0000856 | 0.995 | 218.11302 | [M+H]+ | Positive | 6737.35 |
| N-Acetylglucosamine | Carbohydrate | Amino sugar and Nucleotide sugar Metabolism | HMDB0000215 | 1.188 | 256.0589 | [M+Cl]- | Negative_FA | 749506.3 |
| N-Acetylglucosamine 1-phosphate | Carbohydrate | Amino sugar and Nucleotide sugar Metabolism | HMDB0001367 | 1.175 | 300.03915 | [M-H]- | Negative_FA | 117306.3 |
| N-Acetylglutamate | Amino Acid | Alanine, Aspartate and Glutamate Metabolism | HMDB0001138 | 1.444 | 188.05597 | [M-H]- | Negative_FA | 68.81 |
| N-Acetylhistamine | Amino Acid | Histidine Metabolism | HMDB0013253 | 1.232 | 154.09726 | [M+H]+ | Positive | 63.28 |
| N-Acetylhistidine | Amino Acid | Histidine Metabolism | HMDB0032055 | 1.157 | 196.07214 | [M-H]- | Negative_FA | 16.76 |
| N-Acetylisoleucine | Amino Acid | Leucine, Isoleucine and Valine Metabolism | HMDB0061684 | 3.301 | 172.09686 | [M-H]- | Negative_NH4HCO3 | 36.23 |
| N-Acetylleucine | Amino Acid | Leucine, Isoleucine and Valine Metabolism | HMDB0011756 | 3.559 | 172.09682 | [M-H]- | Negative_NH4HCO3 | 137.07 |
| N-Acetylneuraminic Acid | Carbohydrate | Amino sugar and Nucleotide sugar Metabolism | HMDB0000230 | 1.204 | 308.09872 | [M-H]- | Negative_FA | 202322.3 |
| N-Acetylphenylalanine | Amino Acid | Phenylalanine Metabolism | HMDB0000512 | 5.394 | 206.08224 | [M-H]- | Negative_FA | 496.22 |
| N-Acetylputrescine | Amino Acid | Polyamine Metabolism | HMDB0002064 | 1.207 | 131.11801 | [M+H]+ | Positive | 178.57 |
| N-Acetyltaurine | Amino Acid | Methionine, Cysteine, SAM and Taurine Metabolism | HMDB0240253 | 1.001 | 166.01718 | [M-H]- | Negative_NH4HCO3 | 88.4 |
| N-Acetyltryptophan | Amino Acid | Tryptophan Metabolism | HMDB0013713 | 5.484 | 245.09323 | [M-H]- | Negative_FA | 15025.22 |
| N-Dodecanoyl-N-methylglycine | Unclassified | Unclassified |  | 6.864 | 270.20715 | [M-H]- | Negative_FA | 589310.6 |
| N-Fructosyl pyroglutamate | Xenobiotics | Natural Product/Food/Plant |  | 1.255 | 290.08807 | [M-H]- | Negative_FA | 621971.8 |
| N-Phenylacetylphenylalanine | Peptide | Acetylated Peptides | HMDB0002372 | 5.741 | 284.12814 | [M+H]+ | Positive | 75185.92 |
| N-phenylcarbamate | Xenobiotics | Chemical |  | 6.282 | 180.10172 | [M+H]+ | Positive | 19.53 |
| N1-Acetylspermine | Amino Acid | Polyamine Metabolism | HMDB0001186 | 1.044 | 245.2334 | [M+H]+ | Positive | 484731.9 |
| N1-Methylguanosine | Nucleotide | Purine Metabolism, Guanine containing | HMDB0001563 | 1.552 | 296.10016 | [M-H]- | Negative_FA | 209179.2 |
| N1/N8-Acetylspermidine | Amino Acid | Polyamine Metabolism | HMDB0001276 | 1.117 | 188.17561 | [M+H]+ | Positive | 5722.42 |
| N6,N6,N6-Trimethyllysine | Amino Acid | Lysine Metabolism | HMDB0001325 | 1.089 | 189.1595 | [M+H]+ | Positive | 67267.68 |
| N6,N6-Dimethyllysine | Amino Acid | Lysine Metabolism | HMDB0013287 | 1.104 | 175.14392 | [M+H]+ | Positive | 40.35 |
| N6-Acetyllysine | Amino Acid | Lysine Metabolism | HMDB0000206 | 1.285 | 189.12328 | [M+H]+ | Positive | 2203.17 |
| N6-Methyllysine | Amino Acid | Lysine Metabolism | HMDB0002038 | 1.066 | 161.12833 | [M+H]+ | Positive | 24.83 |
| N6-Succinyladenosine | Nucleotide | Purine Metabolism, Adenine containing | HMDB0000912 | 2.208 | 384.11472 | [M+H]+ | Positive | 231247.1 |
| Naringenin | Xenobiotics | Natural Product/Food/Plant | HMDB0002670 | 6.061 | 271.06097 | [M-H]- | Negative_FA | 661554.3 |
| Nicotinamide | Cofactors and Vitamins | Nicotinate and Nicotinamide Metabolism | HMDB0001406 | 1.256 | 123.05535 | [M+H]+ | Positive | 67.03 |
| Nicotinic Acid | Cofactors and Vitamins | Nicotinate and Nicotinamide Metabolism | HMDB0001488 | 1.315 | 124.04749 | [M+H]+ | Positive | 52.18 |
| Nitrazepam | Unclassified | Unclassified |  | 1.269 | 282.08514 | [M+H]+ | Positive | 329241 |
| Nonanoic Acid (C9:0) | Lipid | Medium Chain Fatty Acid | HMDB0000847 | 7.425 | 157.12218 | [M-H]- | Negative_NH4HCO3 | 630.17 |
| OMEGA-HYDROXYDODECANOIC ACID | Lipid | Fatty Acid, Monohydroxy | HMDB0002059 | 6.609 | 215.16505 | [M-H]- | Negative_FA | 294324.5 |
| Octadecadienedioate (C18:2-DC) | Lipid | Fatty Acid, Dicarboxylate | HMDB0000782 | 6.571 | 309.20715 | [M-H]- | Negative_FA | 133157.8 |
| Octadecanedioate (C18-DC) | Lipid | Fatty Acid, Dicarboxylate | HMDB0000782 | 6.688 | 313.23892 | [M-H]- | Negative_FA | 1873867 |
| Octadecenedioate (C18:1-DC) | Lipid | Fatty Acid, Dicarboxylate | HMDB0304442 | 6.654 | 311.22287 | [M-H]- | Negative_FA | 104790.6 |
| Octanoic Acid (C8:0) | Lipid | Medium Chain Fatty Acid | HMDB0000482 | 7.193 | 143.10655 | [M-H]- | Negative_NH4HCO3 | 69.9 |
| Octenoylcarnitine | Lipid | Fatty Acid Metabolism (Acyl Carnitine) |  | 5.899 | 286.20084 | [M+H]+ | Positive | 117466.3 |
| Oleic Acid (C18:1) | Lipid | Long Chain Fatty Acid | HMDB0000207 | 8.681 | 281.24838 | [M-H]- | Negative_NH4HCO3 | 201.31 |
| Oleoyl ethanolamide | Lipid | Endocannabinoid (N-acylethanolamine) | HMDB0002088 | 7.413 | 326.30515 | [M+H]+ | Positive | 81246.23 |
| Ornithine | Amino Acid | Urea cycle; Arginine and Proline Metabolism | HMDB0000214 | 1.137 | 131.08168 | [M-H]- | Negative_FA | 39.71 |
| Oxalic Acid | Cofactors and Vitamins | Ascorbate and Aldarate Metabolism | HMDB0002329 | 0.884 | 88.98698 | [M-H]- | Negative_NH4HCO3 | 27.87 |
| Oxoglutaric Acid | Carbohydrate | Citrate cycle (TCA cycle) | HMDB0000208 | 0.882 | 145.01317 | [M-H]- | Negative_NH4HCO3 | 82.03 |
| P-Coumaraldehyde | Xenobiotics | Natural Product/Food/Plant | HMDB0037254 | 2.59 | 147.0442 | [M-H]- | Negative_NH4HCO3 | 14 |
| P-Coumaric Acid | Amino Acid | Tyrosine Metabolism | HMDB0002035 | 4.868 | 163.03926 | [M-H]- | Negative_FA | 40.93 |
| P-Cresol Glucuronide | Amino Acid | Tyrosine Metabolism | HMDB0011686 | 4.786 | 283.08282 | [M-H]- | Negative_NH4HCO3 | 14.24 |
| P-Cresol Sulfate | Xenobiotics | Benzoate Metabolism | HMDB0011635 | 5.022 | 187.00621 | [M-H]- | Negative_NH4HCO3 | 67.31 |
| P-Octopamine | Xenobiotics | Natural Product/Food/Plant | HMDB0004825 | 1.458 | 136.07568 | [M+H]+ | Positive | 311.42 |
| PC 32:0\|PC 16:0_16:0 | Lipid | Glycerophospholipid (phosphatidylcholine) | HMDB0000564 | 10.473 | 734.56879 | [M+H]+ | Positive | 249713.5 |
| PC 34:1\|PC 16:0_18:1 | Lipid | Glycerophospholipid (phosphatidylcholine) | HMDB0007972 | 10.451 | 760.58221 | [M+H]+ | Positive | 49642.18 |
| PC 34:2\|PC 16:1_18:1 | Lipid | Glycerophospholipid (phosphatidylcholine) | HMDB0008005 | 9.792 | 758.5694 | [M+H]+ | Positive | 550212.3 |
| PC 36:2\|PC 18:0_18:2 | Lipid | Glycerophospholipid (phosphatidylcholine) | HMDB0008039 | 10.611 | 786.59735 | [M+H]+ | Positive | 209549.9 |
| PC 36:3\|PC 16:0_20:3 | Lipid | Glycerophospholipid (phosphatidylcholine) | HMDB0007981 | 9.824 | 784.58508 | [M+H]+ | Positive | 70600.27 |
| PC 36:4\|PC 16:0_20:4 | Lipid | Glycerophospholipid (phosphatidylcholine) | HMDB0007982 | 9.61 | 782.5683 | [M+H]+ | Positive | 237485.3 |
| PE 34:2\|PE 16:0_18:2 | Lipid | Glycerophospholipid (phosphatidylethanolamine) | HMDB0008928 | 9.857 | 716.52094 | [M+H]+ | Positive | 10.51 |
| PIPECOLIC ACID | Unclassified | Unclassified |  | 1.306 | 128.07048 | [M-H]- | Positive | 105.95 |
| Palmitic Acid (C16:0) | Lipid | Long Chain Fatty Acid | HMDB0000220 | 8.57 | 255.23232 | [M-H]- | Negative_NH4HCO3 | 3039.61 |
| Palmitoleic Acid (C16:1) | Lipid | Long Chain Fatty Acid | HMDB0003229 | 8.276 | 253.21661 | [M-H]- | Negative_NH4HCO3 | 64.58 |
| Palmitoyl ethanolamide | Lipid | Endocannabinoid (N-acylethanolamine) | HMDB0002100 | 7.44 | 300.2894 | [M+H]+ | Positive | 120713.5 |
| Palmitoylcarnitine | Lipid | Fatty Acid Metabolism (Acyl Carnitine) | HMDB0000222 | 7.607 | 400.34262 | [M+H]+ | Positive | 325385.8 |
| Pantothenate | Cofactors and Vitamins | Pantothenate and CoA Metabolism | HMDB0000210 | 2.435 | 220.11774 | [M+H]+ | Positive | 134746.7 |
| Pentadecanoic Acid (C15:0) | Lipid | Long Chain Fatty Acid | HMDB0000826 | 8.364 | 241.21661 | [M-H]- | Negative_NH4HCO3 | 56.41 |
| Perfluorobutane Sulfonic Acid | Xenobiotics | Chemical | HMDB0246220 | 7.114 | 298.94281 | [M-H]- | Negative_NH4HCO3 | 1278.99 |
| Perfluorobutyric Acid | Xenobiotics | Chemical | HMDB0253102 | 5.316 | 212.97838 | [M-H]- | Negative_NH4HCO3 | 342.34 |
| Phe-Asn | Unclassified | Unclassified |  | 1.716 | 280.12906 | [M+H]+ | Positive | 18.71 |
| Phe-Glu | Peptide | Dipeptide | HMDB0028994 | 2.342 | 295.12863 | [M+H]+ | Positive | 62.16 |
| Phe-Gly | Peptide | Dipeptide | HMDB0028995 | 2.749 | 223.09384 | [M+H]+ | Positive | 81.43 |
| Phe-Ile | Peptide | Dipeptide | HMDB0028998 | 5.092 | 279.1702 | [M+H]+ | Positive | 96.13 |
| Phe-Leu | Peptide | Dipeptide | HMDB0253025 | 5.352 | 279.16998 | [M+H]+ | Positive | 164.37 |
| Phe-Trp | Peptide | Dipeptide | HMDB0029006 | 5.544 | 352.16528 | [M+H]+ | Positive | 42860.49 |
| Phenol Sulfate | Amino Acid | Tyrosine Metabolism | HMDB0060015 | 3.389 | 172.9908 | [M-H]- | Negative_FA | 301.5 |
| Phenylacetaldehyde | Amino Acid | Phenylalanine Metabolism | HMDB0006236 | 1.426 | 121.0649 | [M+H]+ | Positive | 127.46 |
| Phenylacetic Acid | Amino Acid | Phenylalanine Metabolism | HMDB0000209 | 3.297 | 135.04417 | [M-H]- | Negative_FA | 20.71 |
| Phenylephrine | Xenobiotics | Natural Product/Food/Plant | HMDB0002182 | 1.18 | 168.10164 | [M+H]+ | Positive | 85.63 |
| Phenylethanolamine | Xenobiotics | Chemical | HMDB0001065 | 2.295 | 120.08097 | [M-H2O+H]+ | Positive | 1031.87 |
| Phenyllactic Acid | Amino Acid | Phenylalanine Metabolism | HMDB0000779 | 5.345 | 165.05484 | [M-H]- | Negative_FA | 708.63 |
| Phosphate | Energy | Oxidative Phosphorylation | HMDB0001429 | 0.886 | 96.96824 | [M-H]- | Negative_NH4HCO3 | 12040.54 |
| Phosphoenolpyruvate | Carbohydrate | Glycolysis, Gluconeogenesis, and Pyruvate Metabolism | HMDB0000263 | 0.908 | 166.98218 | [M-H]- | Negative_NH4HCO3 | 9.26 |
| Phosphoethanolamine | Lipid | Glycerolipid and Phospholipid Metabolism | HMDB0000224 | 0.917 | 140.00945 | [M-H]- | Negative_NH4HCO3 | 72.92 |
| Phosphoserine | Amino Acid | Glycine, Serine and Threonine Metabolism | HMDB0000272 | 0.917 | 184.00121 | [M-H]- | Negative_NH4HCO3 | 39.37 |
| Phosphotyrosine | Amino Acid | Tyrosine Metabolism | HMDB0006049 | 1.063 | 260.02319 | [M-H]- | Negative_NH4HCO3 | 9875.77 |
| Phthalic acid | Unclassified | Unclassified |  | 6.35 | 149.02316 | [M-H2O+H]+ | Positive | 13.6 |
| Piperidine | Xenobiotics | Natural Product/Food/Plant | HMDB0034301 | 1.453 | 86.09694 | [M+H]+ | Positive | 3546.4 |
| Piperine | Xenobiotics | Natural Product/Food/Plant | HMDB0029377 | 6.484 | 286.14365 | [M+H]+ | Positive | 229249.9 |
| Pro-Gly-Pro | Unclassified | Unclassified |  | 1.578 | 270.14456 | [M+H]+ | Positive | 350767.7 |
| Pro-Hydroxy-Pro | Amino Acid | Urea cycle; Arginine and Proline Metabolism | HMDB0006695 | 0.943 | 229.11275 | [M+2H]2+ | Positive | 29.42 |
| Prolinamide | Xenobiotics | Natural Product/Food/Plant | HMDB0253910 | 1.07 | 115.08676 | [M+H]+ | Positive | 13.58 |
| Prolylproline | Unclassified | Unclassified |  | 1.466 | 213.1232 | [M+H]+ | Positive | 891.53 |
| Propane-1,2,3-Tricarboxylic Acid | Xenobiotics | Natural Product/Food/Plant | HMDB0031193 | 1.371 | 175.0242 | [M-H]- | Negative_FA | 58.5 |
| Propanoic Acid | Carbohydrate | Propanoate Metabolism | HMDB0000237 | 1.041 | 73.02831 | [M-H]- | Negative_NH4HCO3 | 4116.34 |
| Propionylcarnitine | Lipid | Fatty Acid Metabolism (Acyl Carnitine) | HMDB0000824 | 1.585 | 218.13841 | [M+H]+ | Positive | 52653.04 |
| Pseudouridine | Nucleotide | Pyrimidine Metabolism, Uracil containing | HMDB0000767 | 0.902 | 243.07516 | [M-H]- | Negative_NH4HCO3 | 38.97 |
| Putrescine | Amino Acid | Polyamine Metabolism | HMDB0001414 | 1.037 | 89.1079 | [M+H]+ | Positive | 360.61 |
| Pyridoxal | Cofactors and Vitamins | Vitamin B6 Metabolism | HMDB0001545 | 1.379 | 168.07307 | [M+H]+ | Positive | 38.24 |
| Pyridoxal 5'-Phosphate | Cofactors and Vitamins | Vitamin B6 Metabolism | HMDB0001491 | 1.165 | 248.03539 | [M+H]+ | Positive | 61272.68 |
| Pyridoxamine | Cofactors and Vitamins | Vitamin B6 Metabolism | HMDB0001431 | 1.08 | 169.09456 | [M+H]+ | Positive | 27.24 |
| Pyridoxate | Cofactors and Vitamins | Vitamin B6 Metabolism | HMDB0000017 | 3.159 | 182.04497 | [M-H]- | Negative_NH4HCO3 | 75.29 |
| Pyridoxine | Cofactors and Vitamins | Vitamin B6 Metabolism | HMDB0000239 | 1.43 | 170.08269 | [M+H]+ | Positive | 108.41 |
| Pyrocatechuic Acid | Amino Acid | Tyrosine Metabolism | HMDB0000397 | 4.189 | 153.01826 | [M-H]- | Negative_NH4HCO3 | 39.24 |
| Pyroglutamic Acid | Amino Acid | Glutathione Metabolism | HMDB0000267 | 1.444 | 130.04993 | [M+H]+ | Positive | 87.73 |
| Pyroglutamine | Amino Acid | Alanine, Aspartate and Glutamate Metabolism | HMDB0062558 | 1.208 | 129.06599 | [M-H]- | Positive | 25.21 |
| Pyroglutamylisoleucine | Peptide | Dipeptide | HMDB0341381 | 4.615 | 241.11909 | [M-H]- | Negative_FA | 1630.4 |
| Pyroglutamylleucine | Peptide | Dipeptide | HMDB0341382 | 3.234 | 241.1185 | [M-H]- | Negative_NH4HCO3 | 28.29 |
| Pyrrolidine | Xenobiotics | Natural Product/Food/Plant | HMDB0031641 | 1.066 | 72.08139 | [M+H]+ | Positive | 302.79 |
| Pyruvic Acid | Carbohydrate | Glycolysis, Gluconeogenesis, and Pyruvate Metabolism | HMDB0000243 | 1.251 | 87.00779 | [M-H]- | Negative_FA | 534.33 |
| Quinoline | Unclassified | Unclassified |  | 5.825 | 130.06508 | [M+H]+ | Positive | 94.41 |
| Riboflavin | Cofactors and Vitamins | Vitamin B2 Metabolism | HMDB0000244 | 4.475 | 377.14584 | [M+H]+ | Positive | 9.14 |
| SM 14:0;2O/28:2 | Unclassified | Unclassified |  | 9.947 | 813.68439 | [M+H]+ | Positive | 243689.8 |
| SM 34:0;O2 | Lipid | Sphingolipid Metabolism |  | 10.149 | 705.5907 | [M+H]+ | Positive | 223733.3 |
| SM 34:1;O2\|SM 18:1;O2/16:0 | Lipid | Sphingolipid Metabolism | HMDB0010169 | 9.836 | 703.57422 | [M+H]+ | Positive | 277646.6 |
| SM 34:2;O2\|SM 18:2;O2/16:0 | Lipid | Sphingolipid Metabolism | HMDB0240638 | 9.258 | 701.5589 | [M+H]+ | Positive | 123816.2 |
| Saccharin | Unclassified | Unclassified |  | 2.867 | 181.99133 | [M-H]- | Negative_FA | 2611.52 |
| Salicylic acid | Xenobiotics | Drug | HMDB0001895 | 5.87 | 137.02362 | [M-H]- | Negative_FA | 125.62 |
| Salsolinol | Xenobiotics | Natural Product/Food/Plant | HMDB0042012 | 1.235 | 180.10201 | [M+H]+ | Positive | 273.24 |
| Sarcosine | Amino Acid | Glycine, Serine and Threonine Metabolism | HMDB0000271 | 1.005 | 88.0392 | [M-H]- | Negative_NH4HCO3 | 38.55 |
| Sebacate (C10-DC) | Lipid | Fatty Acid, Dicarboxylate | HMDB0000792 | 6.074 | 201.11266 | [M-H]- | Negative_FA | 52.39 |
| Sedoheptulose 7-Phosphate | Carbohydrate | Pentose phosphate pathway | HMDB0001068 | 0.918 | 289.03687 | [M-H]- | Negative_NH4HCO3 | 137.39 |
| Ser-Ile | Peptide | Dipeptide | HMDB0029042 | 5.724 | 219.1338 | [M+H]+ | Positive | 15.25 |
| Ser-Leu | Peptide | Dipeptide | HMDB0029043 | 2.814 | 219.13382 | [M+H]+ | Positive | 106.1 |
| Ser-Phe | Peptide | Dipeptide | HMDB0029046 | 1.779 | 253.11588 | [M+H]+ | Positive | 24.15 |
| Ser-Pro | Unclassified | Unclassified |  | 1.278 | 203.1026 | [M+H]+ | Positive | 115.19 |
| Ser-Tyr | Peptide | Dipeptide | HMDB0029051 | 1.644 | 269.11224 | [M+H]+ | Positive | 27.82 |
| Ser-Val | Peptide | Dipeptide | HMDB0029052 | 1.542 | 205.11798 | [M+H]+ | Positive | 74.92 |
| Skatole | Unclassified | Unclassified |  | 5.751 | 130.06522 | [M-H]- | Negative_FA | 87.84 |
| Spermidine | Amino Acid | Polyamine Metabolism | HMDB0001257 | 1.013 | 146.16484 | [M+H]+ | Positive | 409543 |
| Sphinganine | Lipid | Sphingolipid Metabolism | HMDB0000269 | 7.576 | 302.30481 | [M+H]+ | Positive | 326750.1 |
| Stachydrine | Xenobiotics | Natural Product/Food/Plant | HMDB0004827 | 1.217 | 144.1019 | [M+H]+ | Positive | 174.72 |
| Stearic Acid (C18:0) | Lipid | Long Chain Fatty Acid | HMDB0000827 | 9.256 | 283.26392 | [M-H]- | Negative_NH4HCO3 | 2595.49 |
| Stearoylcholine | Lipid | Fatty Acid Metabolism (Acyl Choline) | HMDB0240648 | 9.162 | 370.36789 | [M+H]+ | Positive | 146101.7 |
| Suberate (C8-DC) | Lipid | Fatty Acid, Dicarboxylate | HMDB0000893 | 5.361 | 173.08124 | [M-H]- | Negative_FA | 34.1 |
| Succinic Acid | Carbohydrate | Citrate cycle (TCA cycle) | HMDB0000254 | 0.884 | 117.01817 | [M-H]- | Negative_NH4HCO3 | 1426.1 |
| Succinylcarnitine (C4-DC) | Carbohydrate | Citrate cycle (TCA cycle) | HMDB0061717 | 1.325 | 262.12845 | [M+H]+ | Positive | 24.47 |
| Sucrose | Carbohydrate | Polysaccharides and Oligosaccharides | HMDB0000258 | 1.021 | 341.10886 | [M-H]- | Negative_NH4HCO3 | 373.11 |
| Taurine | Amino Acid | Methionine, Cysteine, SAM and Taurine Metabolism | HMDB0000251 | 0.966 | 124.00626 | [M-H]- | Negative_NH4HCO3 | 415.26 |
| Taurocholic Acid | Lipid | Bile Acid, taurine conjungate | HMDB0000036 | 6.357 | 514.28473 | [M-H]- | Negative_FA | 107349.8 |
| Taurodeoxycholic Acid | Lipid | Bile Acid, taurine conjungate | HMDB0000896 | 6.522 | 498.29019 | [M-H]- | Negative_FA | 410624 |
| Tetradecanedioate (C14-DC) | Lipid | Fatty Acid, Dicarboxylate | HMDB0000872 | 6.487 | 257.17606 | [M-H]- | Negative_FA | 207047.2 |
| Tetradecyl diethanolamine | Lipid | Endocannabinoid (N-acylethanolamine) |  | 7.076 | 302.30487 | [M+H]+ | Positive | 560606.4 |
| Tetradecylsulfate | Xenobiotics | Chemical |  | 7.041 | 293.1785 | [M-H]- | Negative_FA | 32459.25 |
| Thr-Leu | Peptide | Dipeptide | HMDB0029065 | 3.041 | 233.14946 | [M+H]+ | Positive | 4755.51 |
| Thr-Phe | Peptide | Dipeptide | HMDB0029068 | 3.645 | 267.13361 | [M+H]+ | Positive | 126.17 |
| Threonic Acid | Cofactors and Vitamins | Ascorbate and Aldarate Metabolism | HMDB0000943 | 0.93 | 135.02898 | [M-H]- | Negative_NH4HCO3 | 157.45 |
| Thymidine | Nucleotide | Pyrimidine Metabolism, Thymine containing | HMDB0000273 | 1.822 | 241.08257 | [M-H]- | Negative_FA | 37.15 |
| Thymine | Nucleotide | Pyrimidine Metabolism, Thymine containing | HMDB0000262 | 1.605 | 127.05039 | [M+H]+ | Positive | 217.86 |
| Trans-Cinnamaldehyde | Xenobiotics | Natural Product/Food/Plant | HMDB0003441 | 6.582 | 133.06461 | [M+H]+ | Positive | 12.7 |
| Trigonelline (N'-Methylnicotinate) | Cofactors and Vitamins | Nicotinate and Nicotinamide Metabolism | HMDB0000875 | 1.258 | 138.04631 | [M+H]+ | Positive | 160.03 |
| Triptophenolide | Unclassified | Unclassified |  | 6.919 | 311.16754 | [M-H]- | Negative_FA | 220.97 |
| Trp-Glu | Peptide | Dipeptide | HMDB0029082 | 3.228 | 334.13882 | [M+H]+ | Positive | 145464.8 |
| Tryptamine | Amino Acid | Tryptophan Metabolism | HMDB0000303 | 3.938 | 161.10727 | [M+H]+ | Positive | 16.71 |
| Tyr-Gln | Unclassified | Unclassified |  | 1.408 | 310.13922 | [M+H]+ | Positive | 309562.1 |
| Tyr-Ile | Unclassified | Unclassified |  | 4.239 | 295.16498 | [M+H]+ | Positive | 68.45 |
| Tyrosine O-sulfate | Amino Acid | Tyrosine Metabolism | HMDB0155722 | 1.447 | 260.02341 | [M-H]- | Negative_FA | 197087.5 |
| Undecanedioate (C11-DC) | Lipid | Fatty Acid, Dicarboxylate | HMDB0000888 | 6.239 | 215.12805 | [M-H]- | Negative_FA | 57.52 |
| Undecanoic Acid (C11:0) | Lipid | Medium Chain Fatty Acid | HMDB0000947 | 7.731 | 185.15379 | [M-H]- | Negative_NH4HCO3 | 17.34 |
| Uracil | Nucleotide | Pyrimidine Metabolism, Uracil containing | HMDB0000300 | 1.447 | 113.03484 | [M+H]+ | Positive | 12.88 |
| Urate | Nucleotide | Purine Metabolism, (Hypo)Xanthine/Inosine containing | HMDB0000289 | 0.994 | 167.03426 | [M-H]- | Negative_NH4HCO3 | 1104.42 |
| Urea | Amino Acid | Urea cycle; Arginine and Proline Metabolism | HMDB0000294 | 1.225 | 61.04043 | [M+H]+ | Positive | 244.52 |
| Uridine | Nucleotide | Pyrimidine Metabolism, Uracil containing | HMDB0000296 | 1.293 | 243.06187 | [M-H]- | Negative_NH4HCO3 | 47.03 |
| Urocanate | Amino Acid | Histidine Metabolism | HMDB0062562 | 1.291 | 139.05006 | [M+H]+ | Positive | 209.12 |
| Val-Leu | Peptide | Dipeptide | HMDB0029131 | 3.66 | 231.1703 | [M+H]+ | Positive | 2222555 |
| Val-Met | Peptide | Dipeptide | HMDB0259742 | 2.46 | 249.12613 | [M+H]+ | Positive | 9.46 |
| Val-Phe | Peptide | Dipeptide | HMDB0029134 | 4.144 | 265.15475 | [M+H]+ | Positive | 62.03 |
| Val-Pro | Unclassified | Unclassified |  | 1.809 | 215.13867 | [M+H]+ | Positive | 45.21 |
| Val-Val | Peptide | Dipeptide | HMDB0029140 | 2.029 | 217.15427 | [M+H]+ | Positive | 27.89 |
| Valerophenone | Unclassified | Unclassified |  | 6.729 | 163.11127 | [M+H]+ | Positive | 32.24 |
| Vitamin K1 | Cofactors and Vitamins | Vitamin K Metabolism | HMDB0003555 | 6.289 | 226.17979 | [M+H]+ | Positive | 28828.49 |
| Xanthine | Nucleotide | Purine Metabolism, (Hypo)Xanthine/Inosine containing | HMDB0000292 | 1.016 | 153.04227 | [M+H]+ | Positive | 54.05 |
| Xanthosine | Nucleotide | Purine Metabolism, (Hypo)Xanthine/Inosine containing | HMDB0000299 | 1.54 | 285.08466 | [M+H]+ | Positive | 14.46 |
| cAMP | Nucleotide | Purine Metabolism, Adenine containing | HMDB0000058 | 1.29 | 330.05972 | [M+H]+ | Positive | 800500.8 |
| lithocholic acid | Lipid | Bile Acid | HMDB0000761 | 6.78 | 359.29819 | [M+H-H2O]+ | Positive | 298115.4 |
| nicotine | Xenobiotics | Tobaco Metabolite | HMDB0001934 | 1.323 | 163.12289 | [M+H]+ | Positive | 1013.98 |
| taxifolin | Unclassified | Unclassified |  | 5.183 | 303.05118 | [M-H]- | Negative_FA | 21358.91 |
