# Supplementary material for: Toxicological analysis of metabolites in ischemic stroke based on salivary metabolomics
Source: Front Mol Biosci. 2025 Aug 29;12:1609227. doi: 10.3389/fmolb.2025.1609227 (PMC12425714; doi:10.3389/fmolb.2025.1609227)
Supplement: Supplementary file 9 [file Supplementaryfile4.docx]

UMAP Results

| V1 | V2 | group |
| --- | --- | --- |
| -1.9634001 | 2.10019539 | CON |
| 0.10206926 | -0.3250546 | CON |
| 1.25781578 | -0.570588 | CON |
| -1.358187 | 1.76462794 | CON |
| -1.3356417 | 2.32196971 | CON |
| -1.5437942 | 0.81775551 | CON |
| -0.6845585 | 0.31738194 | CON |
| -1.7194699 | 1.69110807 | CON |
| -0.8267401 | 2.29520683 | CON |
| -1.0736536 | 0.65404548 | CON |
| -0.7161706 | 0.14508356 | CON |
| -0.3155103 | 0.33739831 | CON |
| 0.74804408 | -1.0959211 | CON |
| -1.6393806 | 2.27107196 | CON |
| -0.1578394 | -1.70809 | CON |
| -1.4869194 | 2.23781745 | CON |
| -1.8034676 | 1.3302574 | CON |
| 0.4248149 | -0.8034004 | CON |
| -1.3153105 | 1.11609282 | CON |
| 0.16994548 | -0.4769414 | CON |
| -0.7015779 | -0.235187 | CON |
| -1.688735 | 1.71881962 | CON |
| -1.1788293 | 2.01700926 | CON |
| -1.4656878 | 1.01148825 | CON |
| -1.016772 | 1.34926375 | CON |
| -0.8181853 | 2.4162031 | CON |
| -0.2746884 | 1.06466119 | CON |
| -2.4649967 | 2.14340896 | CON |
| -0.3677814 | -0.2526015 | CON |
| -2.2037078 | 1.96823507 | CON |
| 0.92802972 | -0.535649 | IS |
| 0.32422608 | -1.911463 | IS |
| 0.81025444 | 1.34890599 | IS |
| 0.92379353 | -2.0496217 | IS |
| 0.87455585 | -1.6114891 | IS |
| 0.5463246 | -1.8874832 | IS |
| 1.42683389 | 0.68284515 | IS |
| 1.37171622 | 0.26114856 | IS |
| 0.64730555 | -0.6003059 | IS |
| 0.72053126 | -2.0456662 | IS |
| 0.77649034 | 0.77197009 | IS |
| 0.12358166 | -2.6292165 | IS |
| 1.23222523 | 0.39758656 | IS |
| 0.63392186 | -1.1782907 | IS |
| -0.3025112 | -0.2456588 | IS |
| -0.1767907 | -1.2183618 | IS |
| 1.25829178 | -1.2649953 | IS |
| 0.47607237 | -2.5297654 | IS |
| 0.18177089 | 0.92426914 | IS |
| 0.13572495 | -1.353518 | IS |
| -0.0903864 | -0.792485 | IS |
| 0.00398573 | 0.00471157 | IS |
| 0.6806011 | -1.3054312 | IS |
| 1.0690583 | -0.6910194 | IS |
| 0.42258512 | 1.17407243 | IS |
| 0.62733446 | -2.6141282 | IS |
| -0.099184 | -1.9372929 | IS |
| -0.4759144 | -1.5158081 | IS |
| 1.55787563 | -0.8723826 | IS |
| 1.31189736 | -2.1504548 | IS |
| 1.5006873 | -0.2417443 | IS |
| -0.5232347 | 2.08268639 | IS |
| 1.29166096 | -1.5456014 | IS |
| 1.41112172 | 0.30545304 | IS |
| 1.62786894 | -0.0242423 | IS |
| 1.84228186 | -0.6653756 | IS |
| 0.77305763 | 1.50311185 | IS |
| 0.62575802 | 1.0087825 | IS |
| 0.34716034 | -2.7977927 | IS |
| 0.60175228 | 0.12838232 | IS |
